# Modern cemented total knee arthroplasty design shows a higher incidence of radiolucent lines compared to its predecessor

**DOI:** 10.1007/s00167-018-5130-0

**Published:** 2018-09-22

**Authors:** Kevin Staats, Torben Wannmacher, Valerie Weihs, Ulrich Koller, Bernd Kubista, Reinhard Windhager

**Affiliations:** Department of Orthopedics and Trauma Surgery, Medical University of Vienna, Waehringer Guertel 18-20, 1090 Vienna, Austria

**Keywords:** Primary TKA, Knee arthroplasty, Attune, Radiolucency, Radiolucent lines, Total knee replacement, TKA, TKR, Tibial loosening

## Abstract

**Purpose:**

To prevent early failure it is necessary to evaluate modern TKA system for possible shortcomings during implantation. The aim of this study was to evaluate the radiographic outcome and short-term survival of a modern cemented primary TKA system compared to its predecessor.

**Methods:**

The authors reviewed 529 primary cemented TKAs [276 Attune (ATT) and 253 PFC Sigma (PFC)], which were implanted between 2014 and 2017 concerning the radiographic outcome and short-term survival. Radiographs were taken before discharge, 6 weeks, 6 months and 12 months postoperatively. Radiographic analysis was performed by two independent assessors using the Modern Knee Society Radiographic Evaluation System.

**Results:**

The incidence of radiolucent lines was significantly higher in the ATT group compared with the PFC group 12 months postoperatively (35.1%; *n* = 97 TKAs vs. 7.5%; *n* = 19 TKAs; *p* < 0.001). Survival analysis could not show any differences in revision-free survival or revision rate.

**Conclusion:**

The modern primary TKA system shows an increased number of radiolucent lines, especially on the tibial component in this short-term analysis and may mostly be due to technique-related issues. Patients with those radiolucent lines even though they show no clinical evidence for loosening should be closely monitored at regular intervals. These findings are of vital clinical importance because surgeons should be aware of particular challenges in preparation and cementing technique once they are using this TKA-system.

**Level of evidence:**

Retrospective cohort study, Level III.

**Electronic supplementary material:**

The online version of this article (10.1007/s00167-018-5130-0) contains supplementary material, which is available to authorized users.

## Introduction

Even though total knee arthroplasty (TKA) leads to satisfactory results in the treatment of progressed osteoarthritis, TKA implants constantly undergo further design-adjustments. Some of the primary TKA systems, which were introduced in the 1980s or 1990s achieved excellent long-term survivorship rates also in large Arthroplasty registries [9, 14]. Further achievements in TKA designs together with the evolution of new polyethylene (PE) technologies have helped to decrease body wear and, therefore, minimize osteolysis [16, 20, 21].

Anyhow, whereas infection is the most common cause for revision in the first 2 years postoperatively the leading cause for long-term revisions is still represented by aseptic loosening [16, 20, 21]. And aseptic revision represents a devastating diagnosis for the patient with the result of poor functional outcome [12]. But the aetiology of loosening changes with time. Loosening observed in short-term analyses most likely reflects failure to gain fixation. Loosening reported in later years is often due to loss of fixation, secondary to bone resorption [7].

Also in contemporary TKA systems there have been reports of early tibial loosening [8]. The radiographic analysis of radiolucent lines represents an established modality for the prediction of component loosening [5, 6].

## Purpose

To avoid unexpected short- to midterm complications it is of vital importance to evaluate the results of a newly introduced TKA system at an early stage. Therefore, the aim of this study was the evaluation of radiographic short-term results and their influence on implant-survivorship. To our knowledge, this is the first study in which these results of this particular TKA system are directly compared with its predecessor. It was hypothesized that there are differences in the radiographic outcome in terms of an unequal higher incidence of radiolucent lines in the modern TKS system.

At this early stage after its introduction of this system the reader should obtain a closer look into possible challenges and pitfalls, especially in terms of preparation and cementing technique when using this system.

## Materials and methods

529 primary cemented TKAs (481 patients), performed between 2014 and 2017, using the Attune (DePuy Synthes, Warsaw, IN, USA) (*n* = 276) or PFC Sigma (DePuy Synthes, Warsaw, IN) (*n* = 253) system. were retrospectively reviewed. Patients between the Attune- (ATT group) and the PFC Sigma-group (PFC group) did not differ in preoperative patients’ basic demographics (Table 1). Follow-up period was inevitably longer in the PFC group (19 months ± 7 months vs. 25 months ± 11 months) due to the fact that implantation-rate of this system was at the peak at the beginning of the observational period and decreased after introduction of its successor. Preoperative templating was used in all included cases. All TKAs were performed by 7 surgeons with certified experience in total joint arthroplasty (TJA). After performing all necessary bone cuts the bone surface was irrigated with 0.9% saline with a pulsatile high-pressure lavage system (JetLavage, Endocon, Heidelberg, Germany) for at least 1 min (flow rate 1200 ml/min). After irrigation, preparation of bone cement was initialized according to the manufactures specifications. All TKAs were fully cemented using 80 g of high-viscosity bone cement (Palacos R + G, Heraeus Medical, Wehrheim, Germany) and the bone cement was prepared using a vacuum mixing system (Palamix, Heraeus Medical, Wehrheim, Germany). After applying vacuum for 10 s, the cement mass was mixed for 25–30 s. During the mixing process the bone surface was meticulously dryed. The mixed bone cement was applied on the tibial bone surface, the tibial keel canal and on the implant surface via cement gun pressurization. The tibial component was inserted and impacted with at least 10 mallet blows. Right after the femoral component was inserted and impacted in the same manner. Implantation of tibial and femoral component was performed in a single step. There were no differences in the cementing technique between the ATT and the PFC-group.

**Table 1:** Patients’ basic demographics from the group with the modern TKA system (ATT) and the group with implantation of its predecessor system (PFC)

Parameter	ATT-group	PFC-group	*p* value
Sex	Male: *n* = 103 (37.3%) Female: *n* = 173 (62.5%)	Male: *n* = 105 (41.5%) Female: *n* = 148 (58.5%)	n.s.
Age	Ø = 69 years SD ± 9 years	Ø = 68 years SD ± 10 years	n.s.
Body height	Ø = 168 cm SD ± 9.6 cm	Ø = 168 cm SD ± 10.3 cm	n.s.
Body weight	Ø = 86.4 kg SD ± 18.5 kg	Ø = 85.9 kg SD ± 17.1 kg	n.s.
Body Mass Index (BMI)	Ø = 30.4 SD ± 5.9	Ø = 30.7 SD ± 5.4	n.s.
Follow-up time	Ø = 19 months SD ± 7 months	Ø= 25 months SD ± 11 months	< 0.001
Mechanical axis			
Preoperative	− 6.8 ± 4.3	− 6.3 ± 3.9	n.s.
Postoperative	− 1.3 ± 1.5	− 1.2 ± 1.3	n.s.
Change	5.4 ± 4.7	5.1 ± 4.1	n.s.

*Ø* mean, *SD* standard deviation, *n.s*. non-significant

All patients received a standard of care protocol for postoperative appointments. This protocol includes a postoperative radiographic evaluation at the day of surgery, at the day of discharge, after 6 weeks, 6 months and 12 months post-implantation as well as annually thereafter. All radiographs are performed in our outpatient clinic in collaboration of the radiology department of our institution. The images together with the results of the clinical examination are stored electronically in our institutional patient database. Anterior–posterior (AP) and lateral standing radiographs of the knee joint were obtained, as well as full-length standing AP-radiographs to assess correction of alignment. To ensure correct positioning of all anatomic landmarks the patient was informed to keep the knee in full extension with his/her feet in slight internal rotation [6, 23]. Plain and oblique radiographs were performed using a Philips Optimus 80 generator. The radiographs were collected from our in our institutional picture archiving and communication system (PACS, Sectra Imtec AB, Linköping, Sweden).

The radiographs were then anonymized and blinded radiographs were saved in DICOM format to be read a second time 4 weeks after intial assessment. Radiological assessment was undertaken using IMPAX EE (AGFA Healthcare) software.

**Fig. 1:**
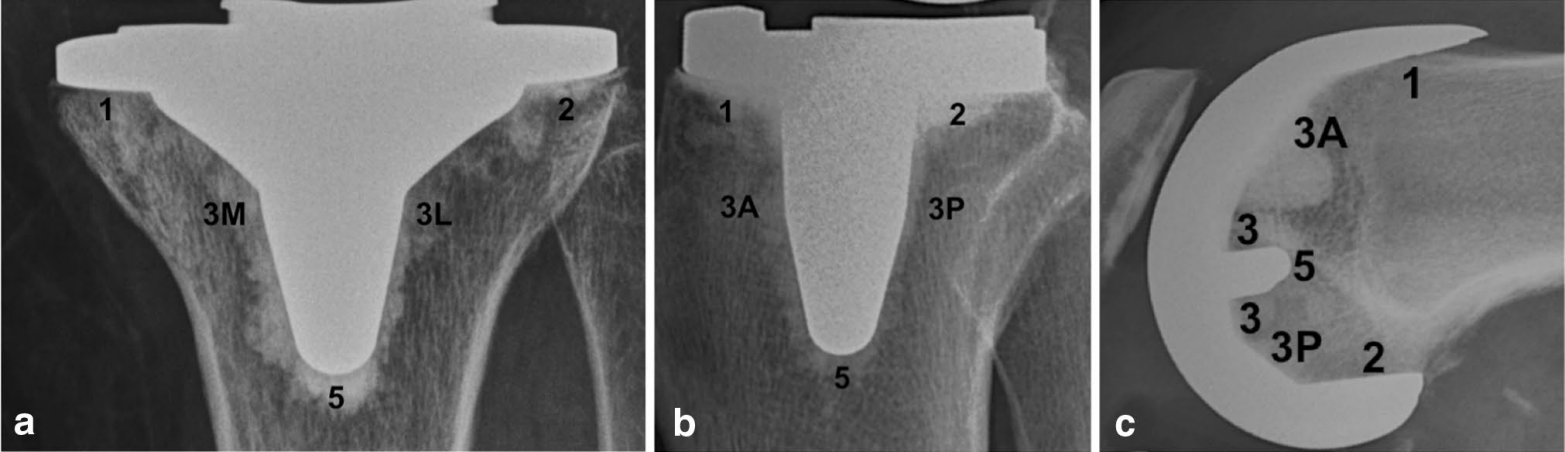
Radiographic evaluation of radiolucent lines according to the “Modern Knee Society Radiographic Evaluation System and Methodology for Total Knee Arthroplasty” [13]: **a** anterior–posterior radiograph of tibial component (3M: medial aspect of the keel; 3L: lateral aspect of the keel); **b** lateral radiograph of tibial component (3A: anterior aspect of the keel; 3P: posterior aspect of the keel); **c** lateral radiograph of femoral component

Two independent readers blinded to the patient’s medical history assessed all radiographs. The blinded assessors were trained to read all radiographs on the basis of the Modern Knee Society Radiographic Evaluation System [13]. Using this radiographic evaluation system the components were divided in different zones for a standardized documentation of radiolucent lines (Fig. 1).

The tibial component in the AP view was divided into 5 different zones (Fig. 1a): (1) medial baseplate, (2) lateral baseplate, (3M) medial aspect of central keel, (3L) lateral aspect of central keel, (5) inferior aspect of central keel.

The tibial component in the lateral view was again divided into 5 different zones (Fig. 1b): (1) anterior baseplate, (2) posterior baseplate, (3A) anterior aspect of central keel, (3P) posterior aspect of central keel, (5) inferior aspect of tibial keel.

The femoral component was evaluated solely in the lateral view and divided into 7 different zones (Fig. 1c): (1) anterior flange, (2) posterior flange, (3A) central box anterior chamfer, (3) base of the peg, (3P) central box posterior chamfer, (5) tip of the peg.

Radiolucent lines were defined as radiolucent intervals either between the implant–cement (I/C) or the cement–bone (C/B) interface. Radiolucencies were documented for each radiograph (Tibia ap, Tibia lateral, Femur lateral) separately. The readers had to document the zones and type of radiolucency (between implant and cement or between cement and bone). Therefore, for each radiograph the reader assigned a number between 0 and 10 (0 = no radiolucency, 1–5 = sections and radiolucency occured between implant and cement, 6–10 = sections and radiolucency occured between cement and bone). Radiolucent lines were only documented if they were detected in two different serial radiographs. Intra- and inter-reader variability was assessed by comparing the independent results of the two readers. Both readers were able to reproduce their own readings from all 529.4 weeks apart: the intraclass-correlation coefficients (ICC) ranged from 0.936 to 1. The agreement between the two readers was excellent to good, depending on examined component and imaging technique; ICC in the AP view was 0.812 (range 0.744–0.987), ICC in lateral tibial component view was 0.697 (range 0.673–0.841) and 0.848 (range 0.737–0.918) in lateral femoral component view. To avoid bias due to a possible memory effect a selection of 140 (70 ATT vs. 70 PFC) randomly chosen blinded cases was again assessed by both readers with a minimum interval of 12 weeks after initial evaluation. Intraclass correlation ranged from 0.733 to 0.941 and an ICC (between both readers) in the AP of 0.748 (range 0.592–0.883), ICC in lateral tibial component view was 0.672 (range 0.621–0.779) and 0.807 (range 0.712–0.873) in lateral femoral component view.

This study was approved by the Institutional Review Board of the Medical University of Vienna (EK-Nr. 2192/2017).

### Statistical analysis

In order to evaluate the differences in basic demographics, clinical and radiographic results between the study group (ATT) and the control group (PFC), an unpaired two-tailed *T* test (numerical variables) and the Chi-square test (for binary variables) were applied. To determine a connection between radiolucent lines and patient-specific parameters a Mann–Whitney *U* test was applied. Revision-free survival and cumulative survival were calculated using a Kaplan–Meier survival analysis. A log-rank-test was applied to detect differences between the observed groups. *p* values of < 0.05 were considered as statistically significant. Statistical analysis was performed using SPSS software, version 23.0 (SPSS Inc., Chicago, USA).

Post-hoc sample size calculation for the assessment of the whole cohort with an effect size of 0.5 and alpha of 0.05 showed a power of 0.99. Post-hoc sample size calculation for the third assessment (70 ATT vs. 70 PFC) with an effect size of 0.5 and alpha of 0.05 showed a power of 0.84.

## Results

**Fig. 2:**
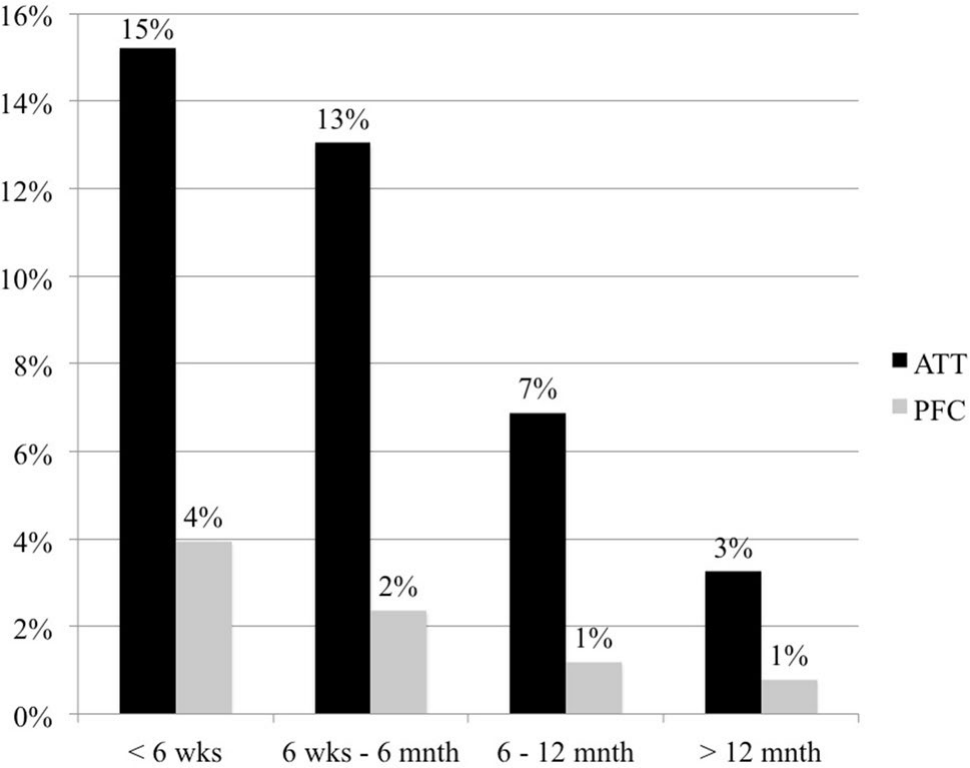
Incidence of radiolucent lines depending on the date of first detection. In the ATT-group 40.8% (*n* = 42) of all radiolucent lines were detectable in the immediate postoperative radiograph (< 6 weeks postoperatively), whereas only 19% (*n* = 4) of all radiolucencies in the PFC-group were visible directly after surgery (*p* = 0.048). In total radiolucent lines were detectable in 37.3% (*n* = 103) of the ATT-group and in 8.3% (*n* = 21) of the PFC-group (*p* < 0.001)

**Fig. 3:**
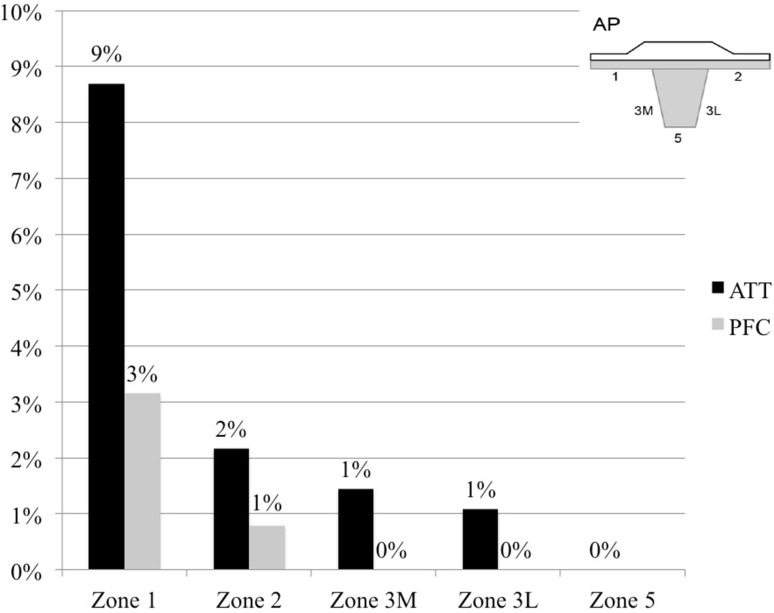
Incidence of radiolucent lines in the anterior–posterior (AP) tibial radiograph measured according to the “Modern Knee Society Radiographic Evaluation System and Methodology for Total Knee Arthroplasty” [13]

**Fig. 4:**
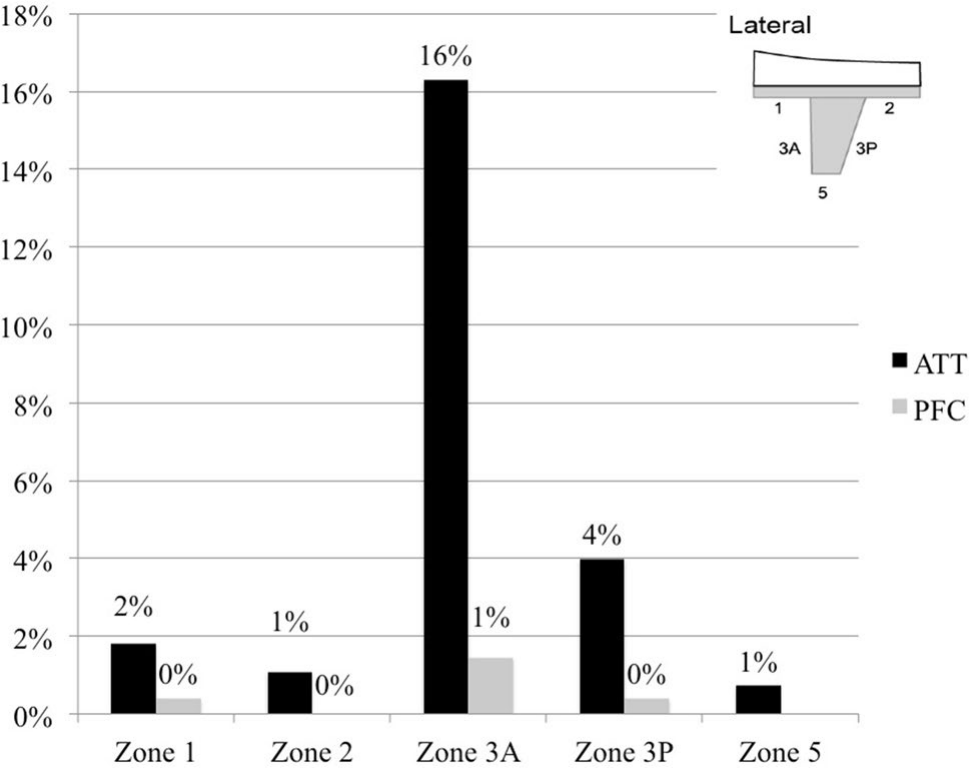
Incidence of radiolucent lines in the lateral tibial radiograph measured according to the “Modern Knee Society Radiographic Evaluation System and Methodology for Total Knee Arthroplasty” [13]

**Fig. 5:**
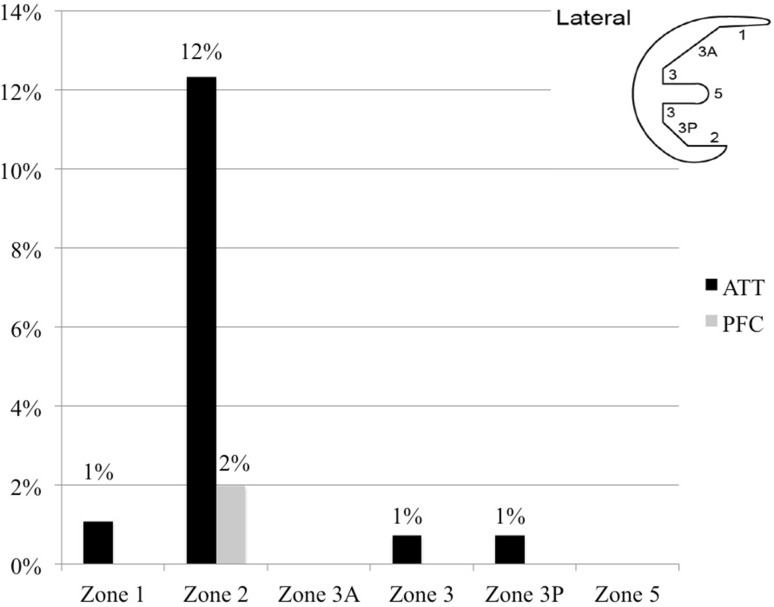
Incidence of radiolucent lines in the lateral femoral radiograph measured according to the “Modern Knee Society Radiographic Evaluation System and Methodology for Total Knee Arthroplasty” [13]

The incidence of radiolucent lines was significantly higher in the ATT group compared with the PFC group after 12 months of evaluation (35.1%; *n* = 97 TKAs vs. 7.5%; *n* = 19 TKAs; *p* < 0.001) (Fig. 2). Figures 3, 4 and 5 display the incidence in dependence on the location of detected radiolucent lines. Table 2 summarizes the differences in the occurrence of radiolucent lines per TKA between the ATT- and PFC-group. No connection between patient-specific parameters (height, weight, body-mass-index, preoperative mechanical axis, postoperative mechanical axis, change in mechanical axis) could be determined (Table 3).

**Table 2:** Summary of all radiolucent lines in the anterior–posterior (AP) tibial, lateral tibial and lateral femoral radiograph in patients with the modern primary TKA system (ATT) and its predecessor system (PFC) after 12 months of follow up

Location, radiolucency	ATT, *n* = 276	PFC, *n* = 253	*p* value
Tibia AP	13.4% (*n* = 37; I/C: *n* = 34)	4% (*n* = 10; I/C: *n* = 5)	0.001
Tibia Lateral	20.3% (*n* = 56; I/C: *n* = 53)	2.4% (*n* = 6; I/C: *n* = 6)	< 0.001
Femur lateral	14.5% (*n* = 40; I/C: *n* = 40)	2% (*n* = 5; I/C: *n* = 5)	< 0.001

*I/C* implant/cement interface, *n.s*. non-significant

**Table 3:** Patient-specific parameters in relation to the incidence of radiolucent lines in the ATT-group

Parameter	Radiolucency	No radiolucency	*p* value
Body height	Ø = 167 cm SD ± 9.5 cm	Ø = 168 cm SD ± 9.9 cm	n.s.
Body weight	Ø = 86.0 kg SD ± 18.9 kg	Ø = 86.6 kg SD ± 17.7 kg	n.s.
Body Mass Index (BMI)	Ø = 30.5 SD ± 5.4	Ø = 30.3 SD ± 6.4	n.s.
Mechanical axis			
Preoperative	− 7.1 ± 4.0	− 6.6 ± 4.5	n.s.
Postoperative	− 1.2 ± 1.5	− 1.3 ± 1.5	n.s.
Change	5.7 ± 4.6	5.1 ± 4.8	n.s.

*n.s*. non-significant

**Fig. 6:**
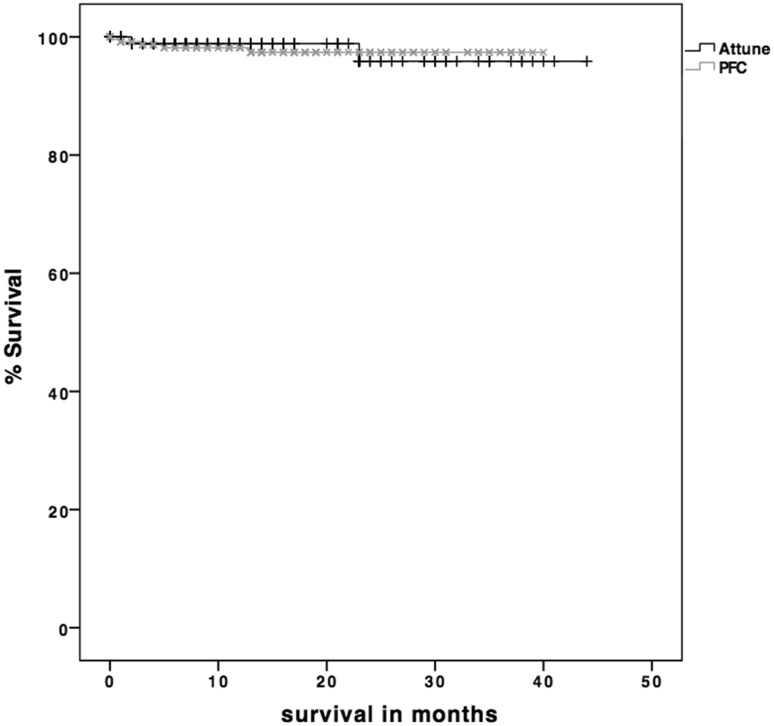
Kaplan–Meier analysis displays the survival of the Attune- and PFC Sigma-cohort. No difference in the revision-free survival (*p* = 0.711) or in the revision rate (*p* = 0.402) was detected between both groups

**Fig. 7:**
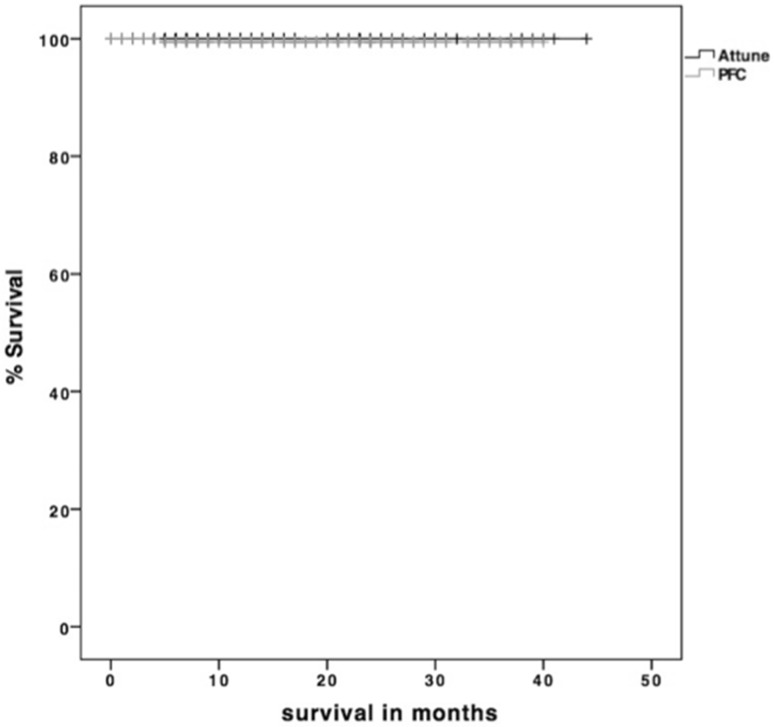
Kaplan–Meier survival analysis did not show any difference concerning aseptic loosening between Attune and PFC Sigma (*p* = 0.388)

In the ATT-group three patients (1.1%) had to undergo revision surgery, but none due to aseptic loosening, whereas, in the PFC-group one out of five patients (2%) with revision surgery had to be revised due to aseptic loosening (Figs. 6, 7).

## Discussion

The most important finding of this study is the highly significant differences in the occurrence of radiolucent lines between the ATT and PFC group.

**Fig. 8:**
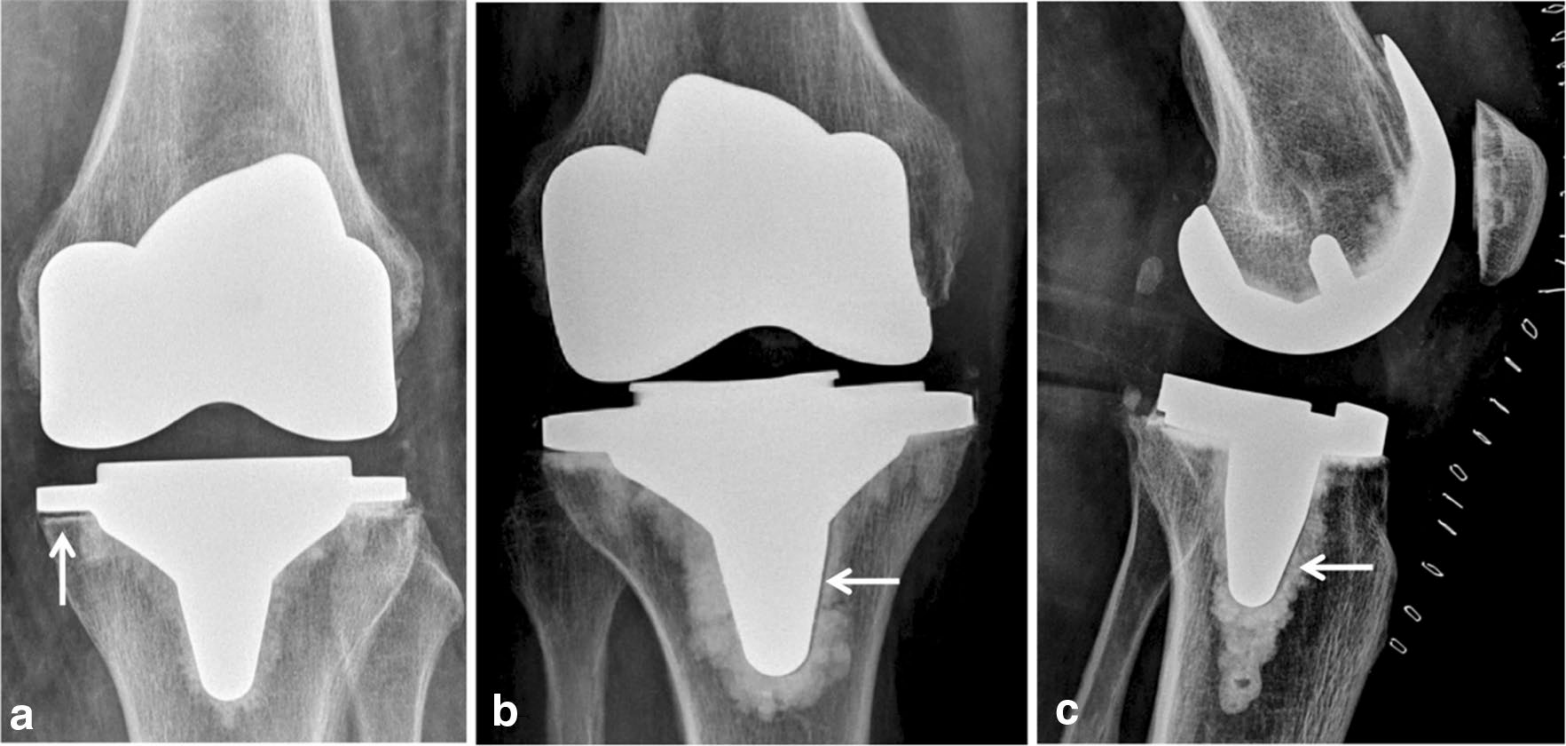
Examples of radiolucent lines in the investigated Attune-group: **a** anterior–posterior (AP) radiograph of a left Attune TKA shows a radiolucent line at the medial tibial baseplate (zone 1, white arrow); **b** AP radiograph of a right Attune TKA displays radiolucency medial aspect of the central keel (zone 3M, white arrow); **c** lateral radiograph of a left Attune-TKA with radiolucency at the anterior aspect of the central keel (zone 3A, white arrow)

Especially the medial tibial baseplate (Fig. 8a) and the anterior aspect of central tibial keel (Fig. 8c) represent predisposed regions for a high rate of radiolucencies in the ATT-group. The majority of radiolucencies occurred at the cement–implant interface, hence concern should be raised since tibial debonding from the cement–implant interface leads to higher revision rate due to aseptic loosening [1].

Reasons for the high incidence of radiolucencies in the ATT-group remain to a certain extent a matter of speculation. Possible influencial factors may be reduced to shortcomings in surgical technique or implant-related issues. Cementation technique still remains a matter of discussion in primary TKA [3, 18, 19, 24]. Kopinski et al. found a connection between the use of high-viscosity cement and early tibial debonding [11]. Anyhow, since high-viscosity cement was used in both groups we do not think that the cement itself had any negative influence concerning decreased attachment on the tibial component. An in-vitro study by Skwara et al. revealed a higher number of failures with fully cemented tibial component compared to a surface cemented component [21]. Rossi et al. found comparable results between surface and fully cementation [18]. All TKAs in our cohorts were fully cemented; therefore, it is doubtful that full cementation may have caused the higher incidence of radiolucencies. However, one influencial factor might be the tibial keel preparation itself. For the modern TKA system the surgeon’s only option was to use a keel-punch, which prepared the tibia for a cement mantle with at least 1 mm around the keel. A combination of high-viscosity cement, the additional preparation for the cement mantle and movement during the interlocking-process (e.g. extension or even hyperextension) may lead to an increased force on the anterior aspect of the tibial baseplate causing the keel shifting dorsally. This mechanism may lead to radiolucent lines, especially around the keel. Kolisek et al. have found excellent results using cemented tibial tray with press-fit cementless keel preparation after 10 years of follow-up [10]. There has been an extensive experience in using a cementless keel punch for the predecessor system at our institution, which supports those findings from Kolisek et al. Meanwhile a line-to-line keel-punch for press-fit tibial preparation is also available for the modern system, which is now used in our institution and needs to be proven for benefits concerning this issue in the future. The tibial surface preparation itself may also contribute to the occurrence of radiolucent lines. Since the majority of radiolucencies in the ap-view have been detected on the medial side of the tibial baseplate, one can argue that this may be due to an insufficient bone cuts. Nevertheless, postoperative analysis of full length standing ap radiographs revealed no higher incidence in malalignment in the ATT-group compared to the PFC group. Partial inaccurate bone cuts (e.g., grooves, bumps) could have occurred during re-cutting the tibial surface. However, those irregularities should be compensated by the viscous bone cement. Therefore, it can be assumed that the implant itself may allow too much movement during the cement interlocking-phase. This may especially be a problem when both components are cemented in a single step. This is the reason why we now tend to perform the cementation of the tibial and femoral component in two separated steps.

Differences at the posterior flange were also detected, which we think are negligible due to the fact that most of those radiolucencies mainly occurred at the most posterior point of the posterior flange. One explanation may be that due to its rather shallow cement pockets we assume that during introduction of the femoral component the cement on the posterior flanges sheared off. Therefore, care should be taken by placing a sufficient amount of cement on the pockets and that the posterior flanges are introduced initially to reduced shear-forces.

This study certainly shows several limitations.

First,only standard radiographs were observed in this study. Fluoroscopically assisted radiographs show superior results compared to standard radiographs [4]. Anyhow, also with standard radiographs a high inter- and intrarater reliability could be obtained. Additionally, radiolucent lines were only considered as positive if they were detectable in two serial radiographs. Second, in this present study we did not include any form of functional assessment and, therefore, cannot provide any information about the level of satisfaction.

Literature about the outcome of the Attune-system is scarce due to its recent availability. Anyhow, Ranawat et al. showed comparable results between both systems with less anterior knee pain and less crepitation in Attune-patients after 2 years’ follow-up [17]. Additionally, Pfitzner et al. could show improved kinematics in terms of an increase of lateral roll-back and therefore decrease of potential patellofemoral pressure [15]. According to our findings we can confirm that this system achieves excellent results regarding the revision rate at very short-term follow-up. Currently, a comparable revision-free survival with its predecessor (Fig. 7) was detected in this present study, which seems encouraging considering the longer experience and somehow completed learning-curve with the PFC system. It is for these reasons why we continue the use in our institution with the aforementioned adaptions in tibial preparation. The revision rates of the Attune in this study are congruent with the results from the Australian Arthroplasty register with a revision rate of 0.4 and 0.6 after 1 year and 1.1 and 2.1 after 3 years [2].

However, the radiographic results of the presented study together with other findings in the literature [22] should raise concern that the design of the tibial component may have its disadvantages even though no evidence for a higher complication- or revision rate could be detected in our study. In the meantime, the company has launched a revised tibial component with additional cement pockets and optimized surface conditions on the tibial base, which should be proven for beneficial influence concerning the occurrence of radiolucent lines in the further future.

## Conclusion

Due to the higher incidence of radiolucent lines, those patients should be closely monitored at regular intervals even though they show no clinical evidence for loosening. Further investigations are needed to evaluate the experience from other users and maybe improve the application of this modern TKA-system.

## Electronic supplementary material

Below is the link to the electronic supplementary material.

Supplementary material 1 (PDF 21961 KB)

Supplementary material 2 (DOCX 21 KB)
