# Supplementary material for: Modern cemented total knee arthroplasty design shows a higher incidence of radiolucent lines compared to its predecessor
Source: Knee Surg Sports Traumatol Arthrosc. 2018 Sep 22;27(4):1148–55. doi: 10.1007/s00167-018-5130-0 (PMC6435629; doi:10.1007/s00167-018-5130-0)

Case 1

preoperative

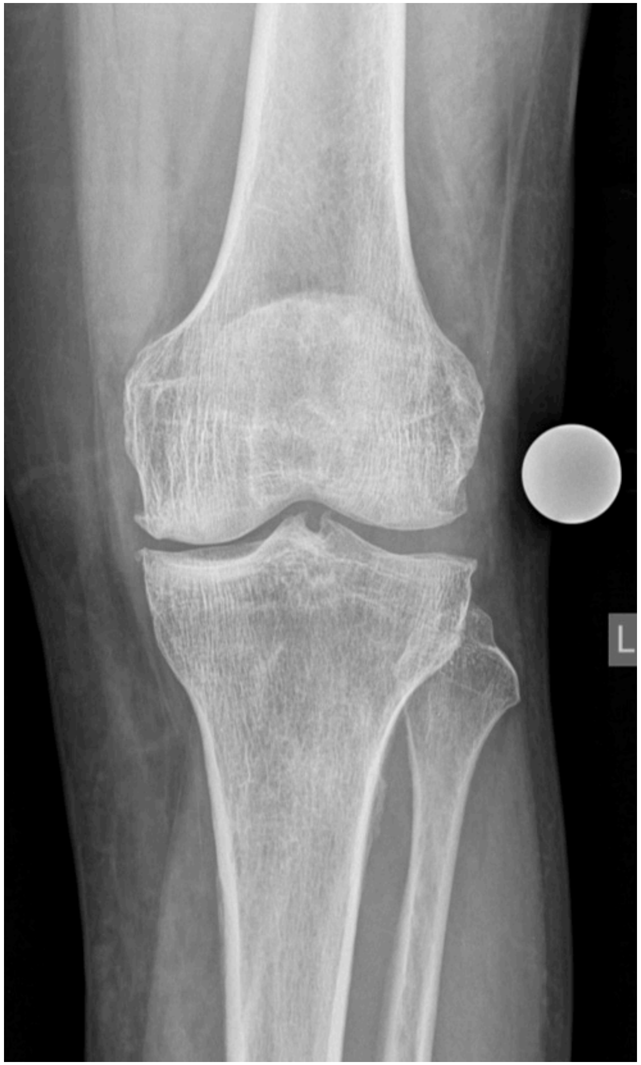

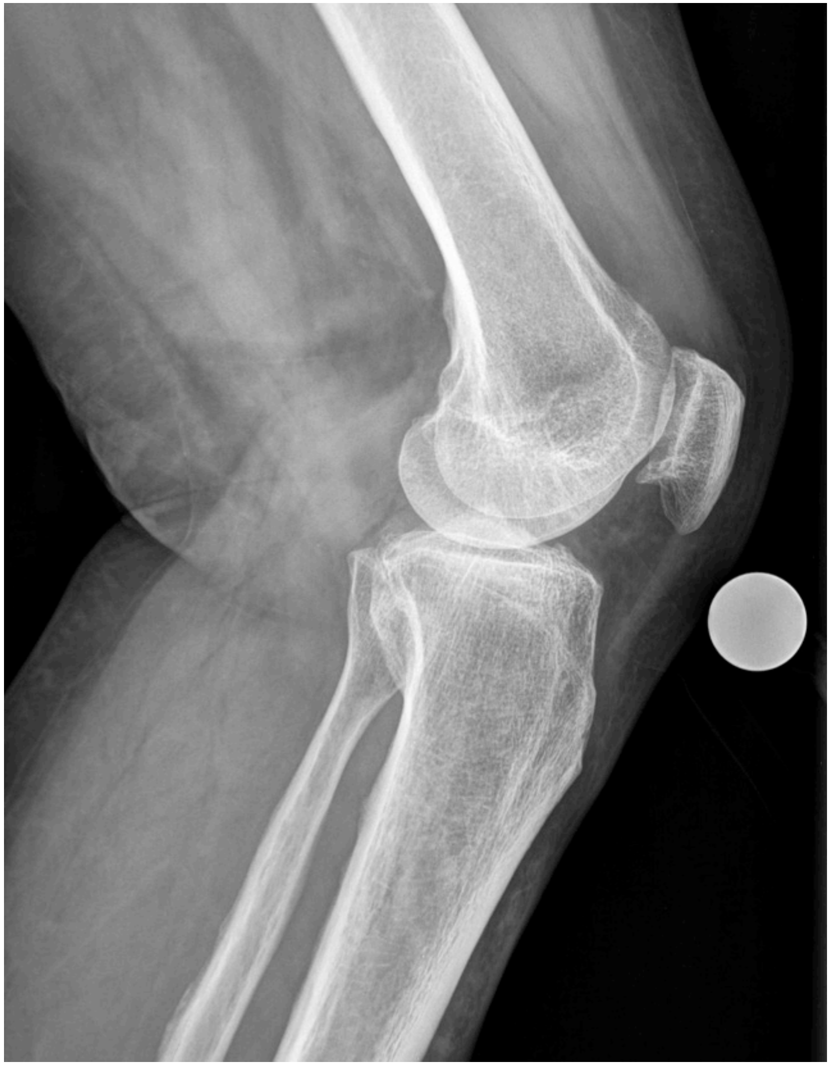

6 weeks postop

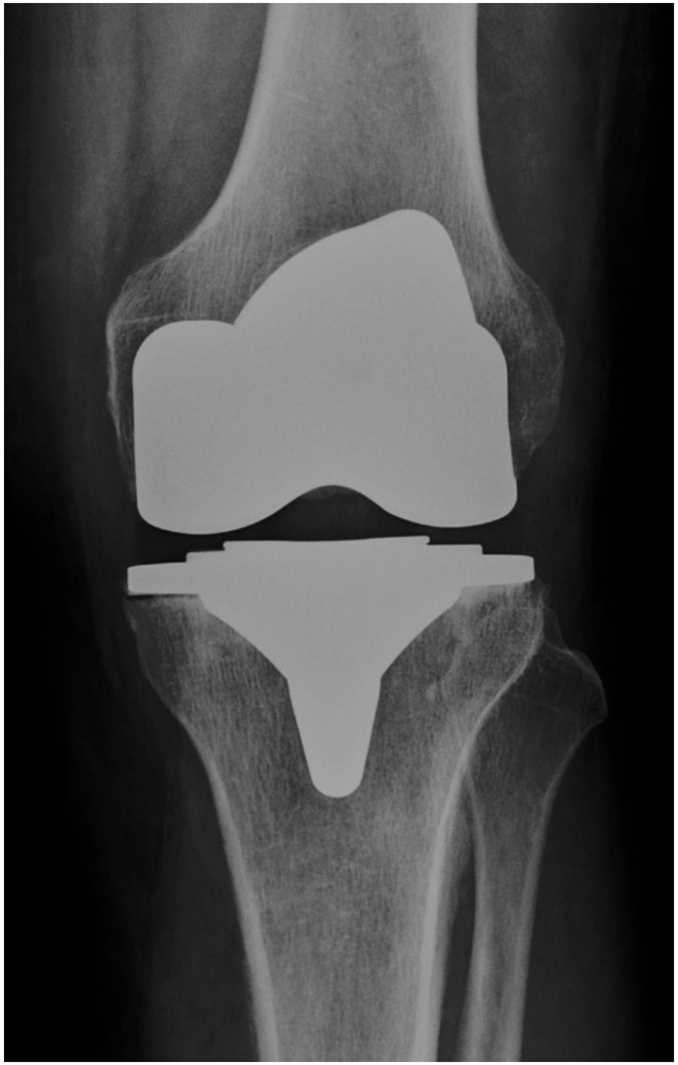

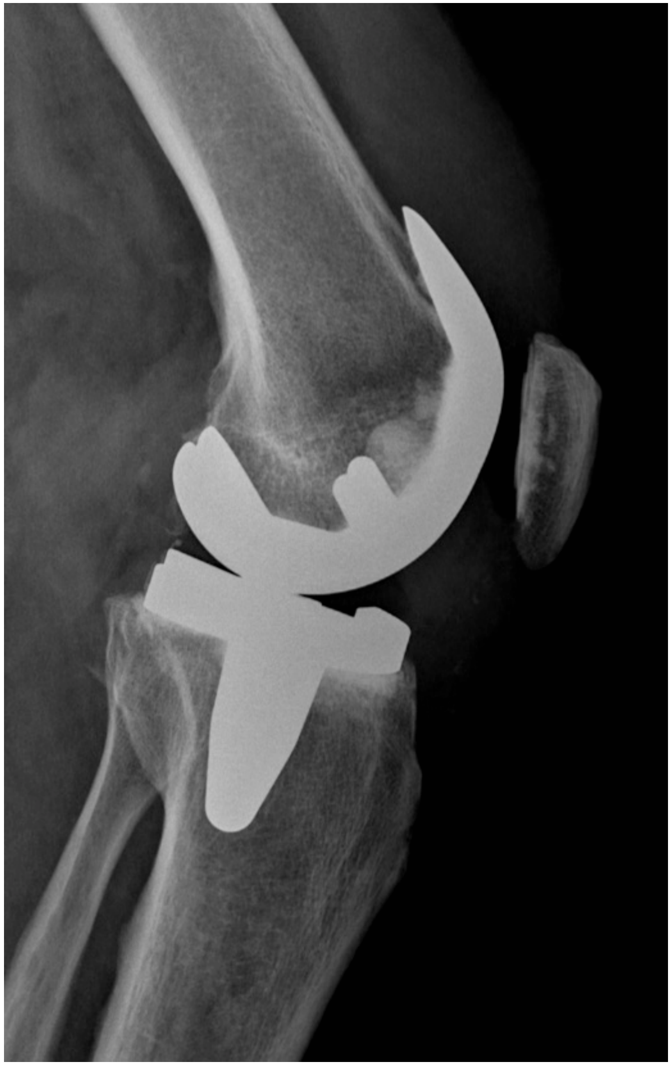

6 months postop

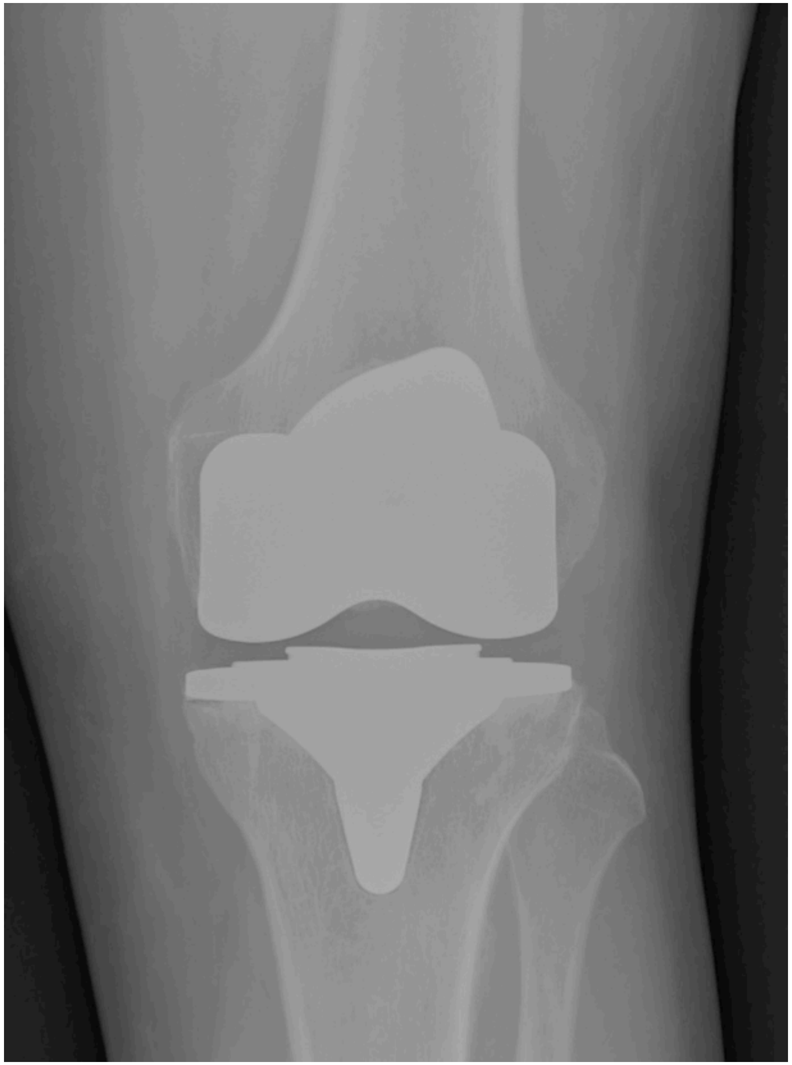

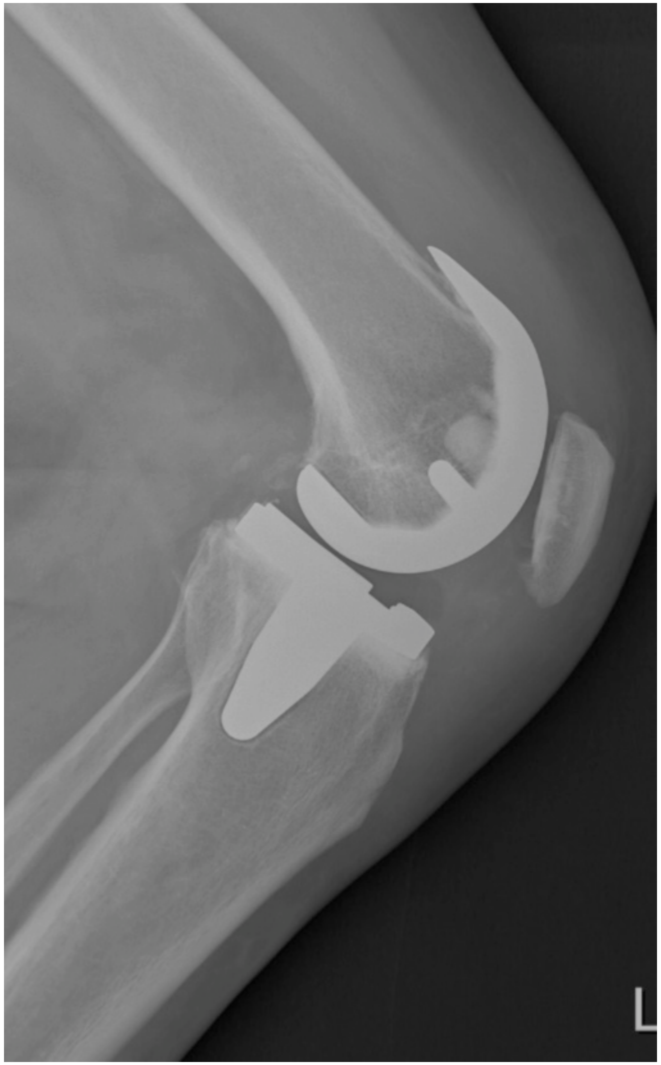

12 months postop

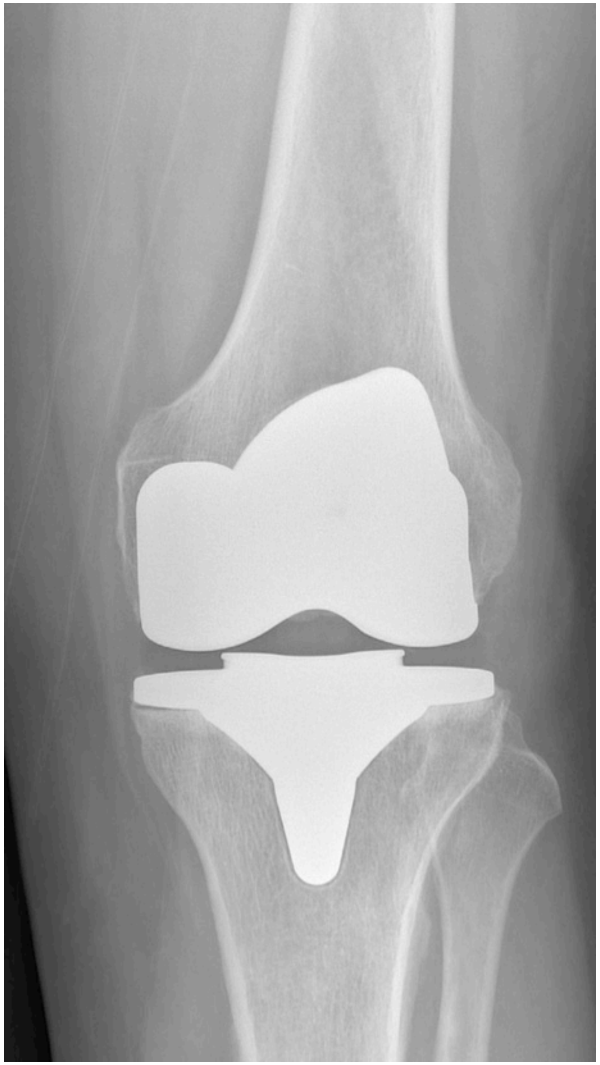

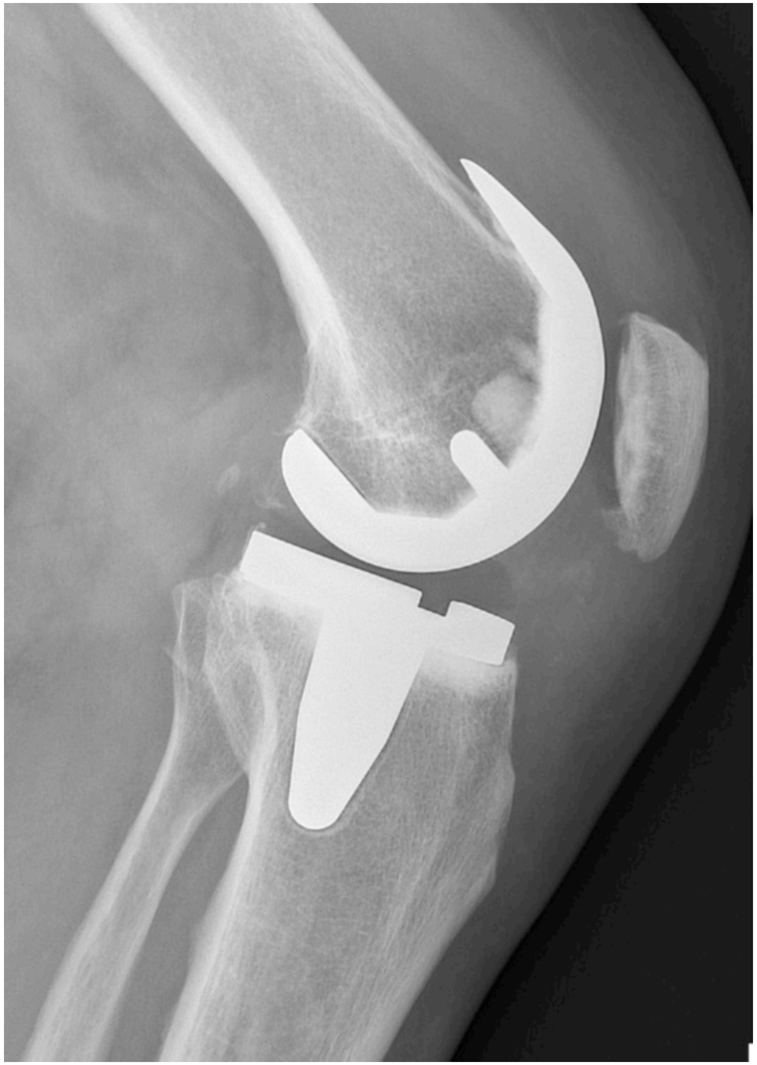

Case 2

preoperative

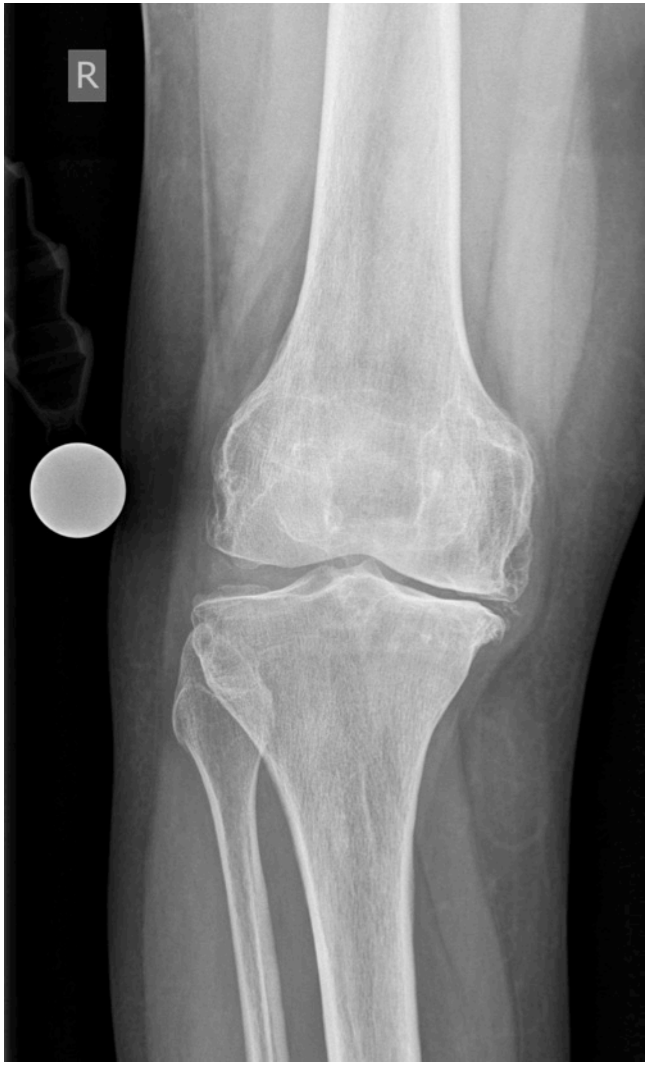

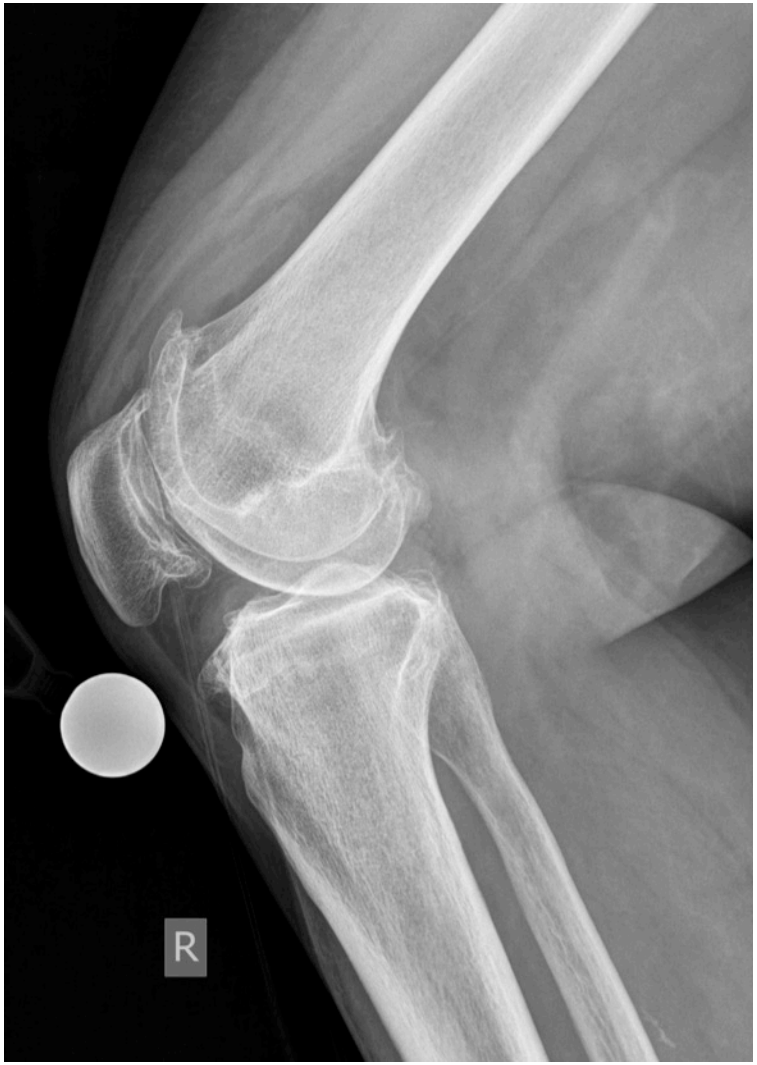

6 weeks postop

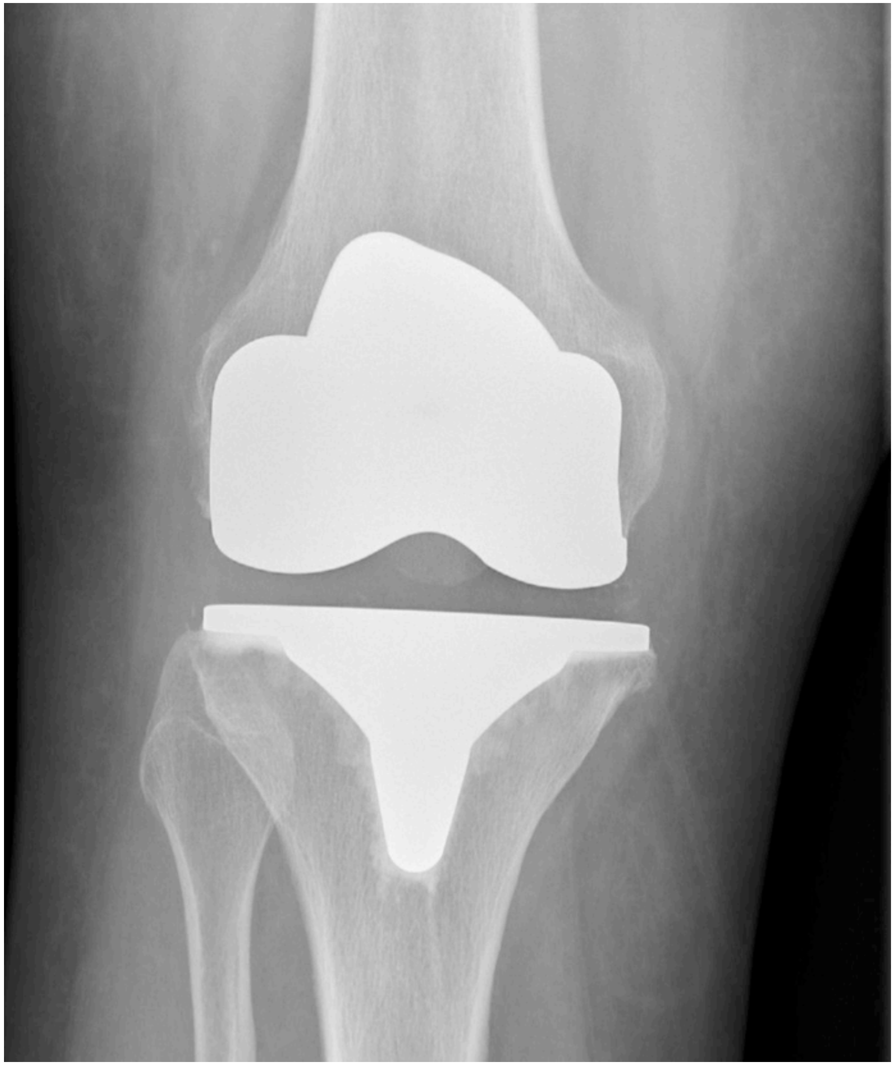

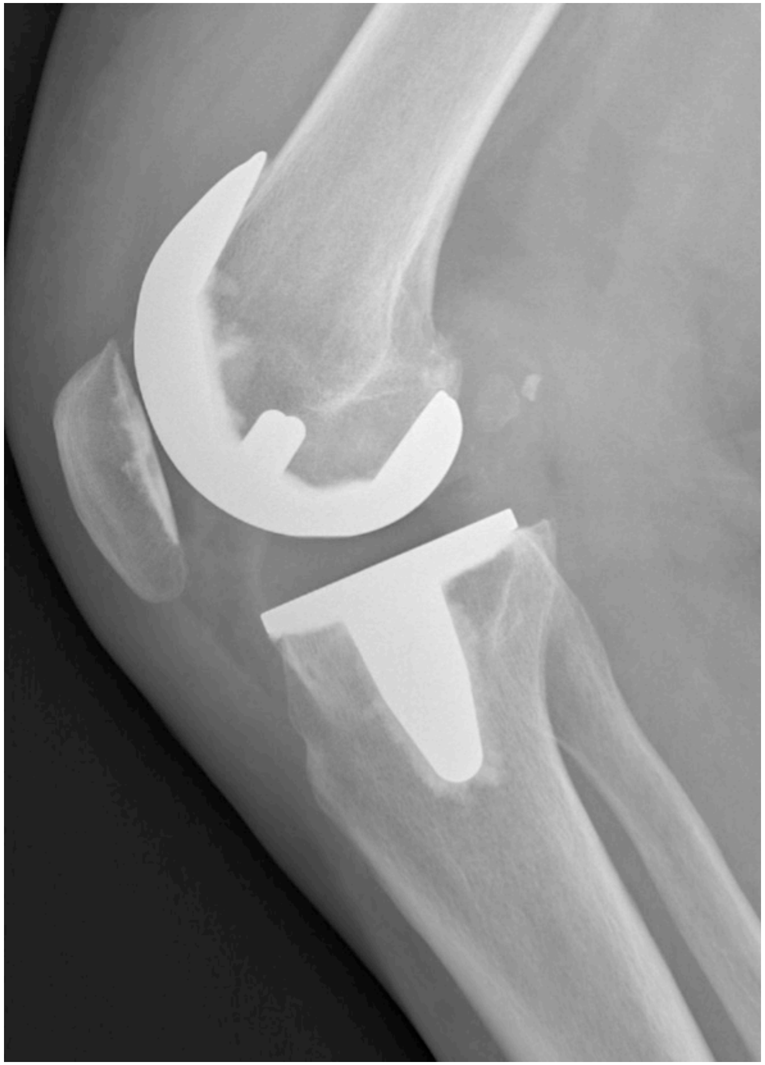

6 months postop

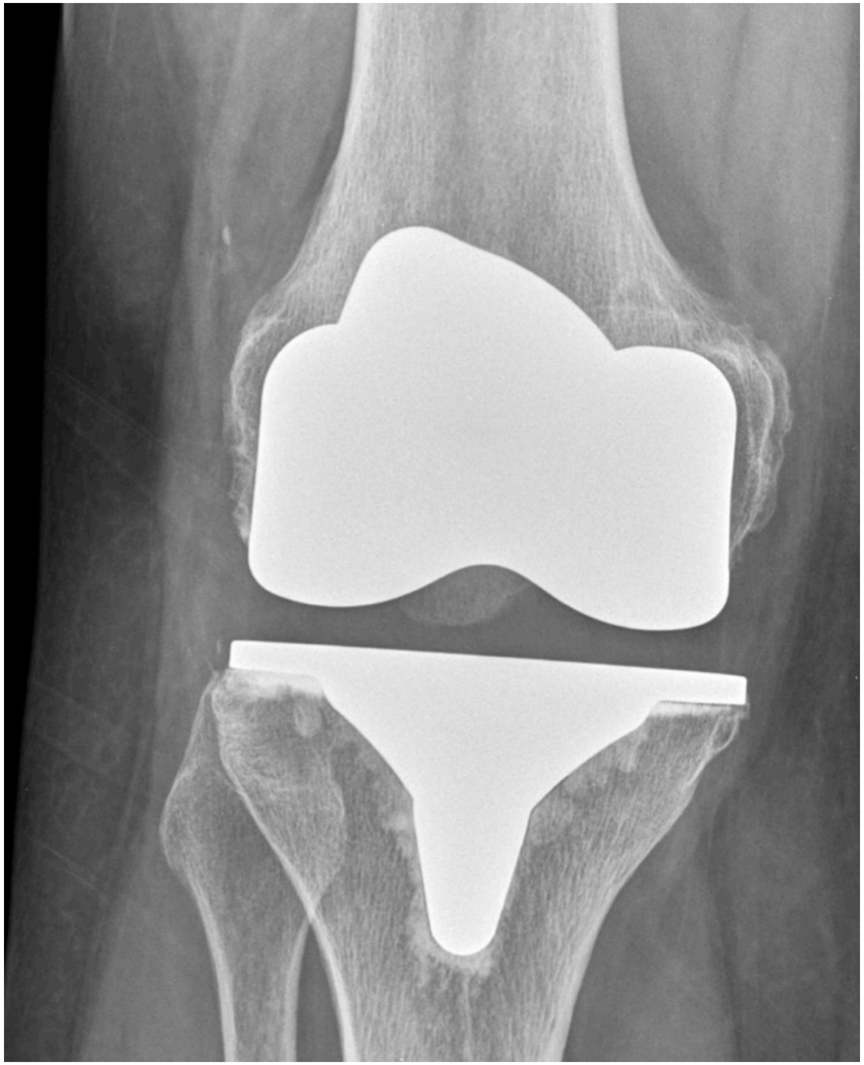

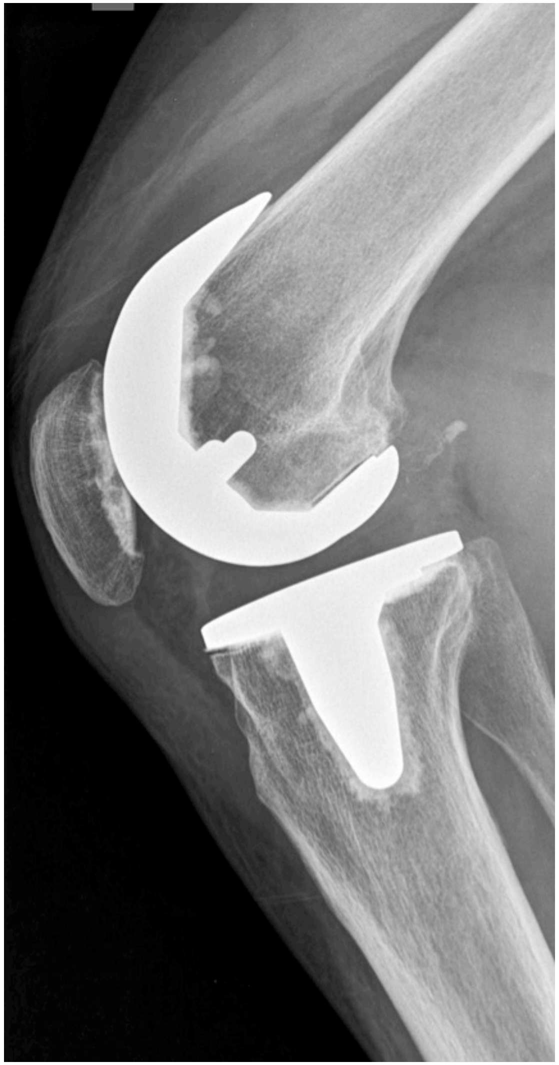

12 months postop

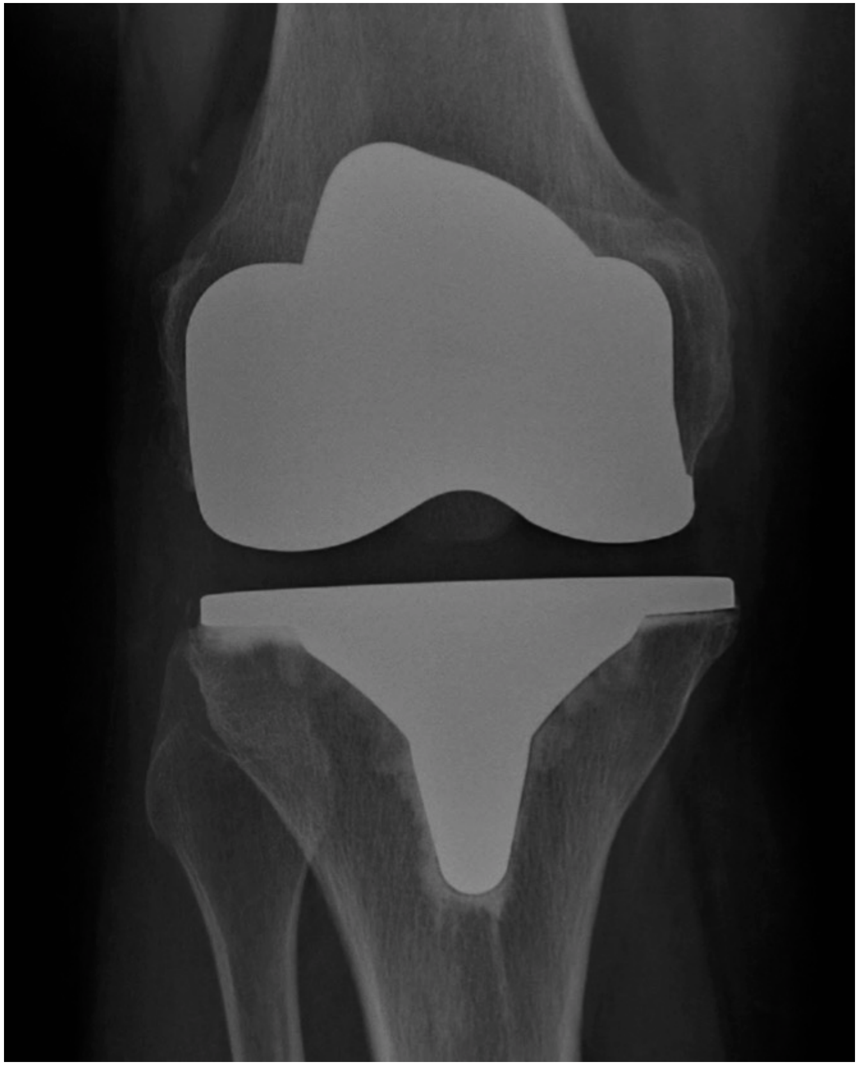

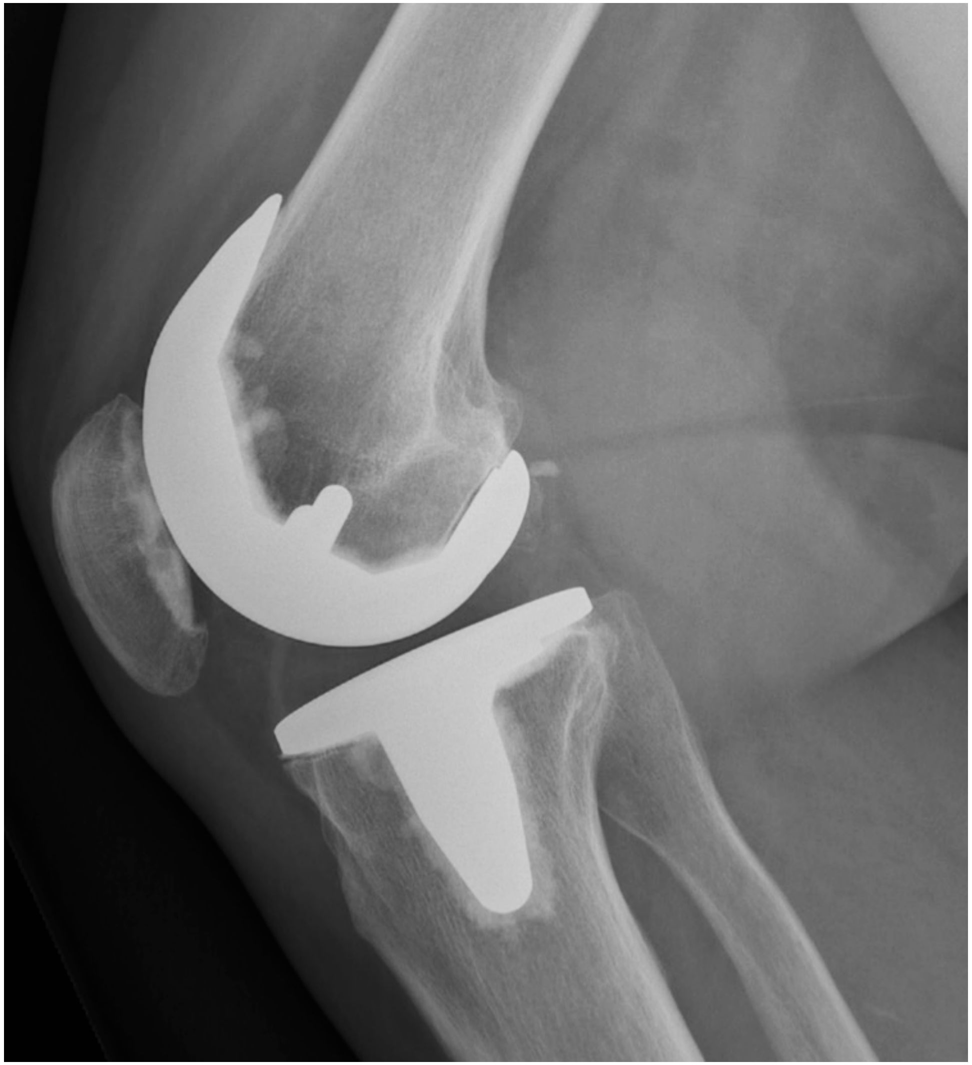

Case 3

preoperative

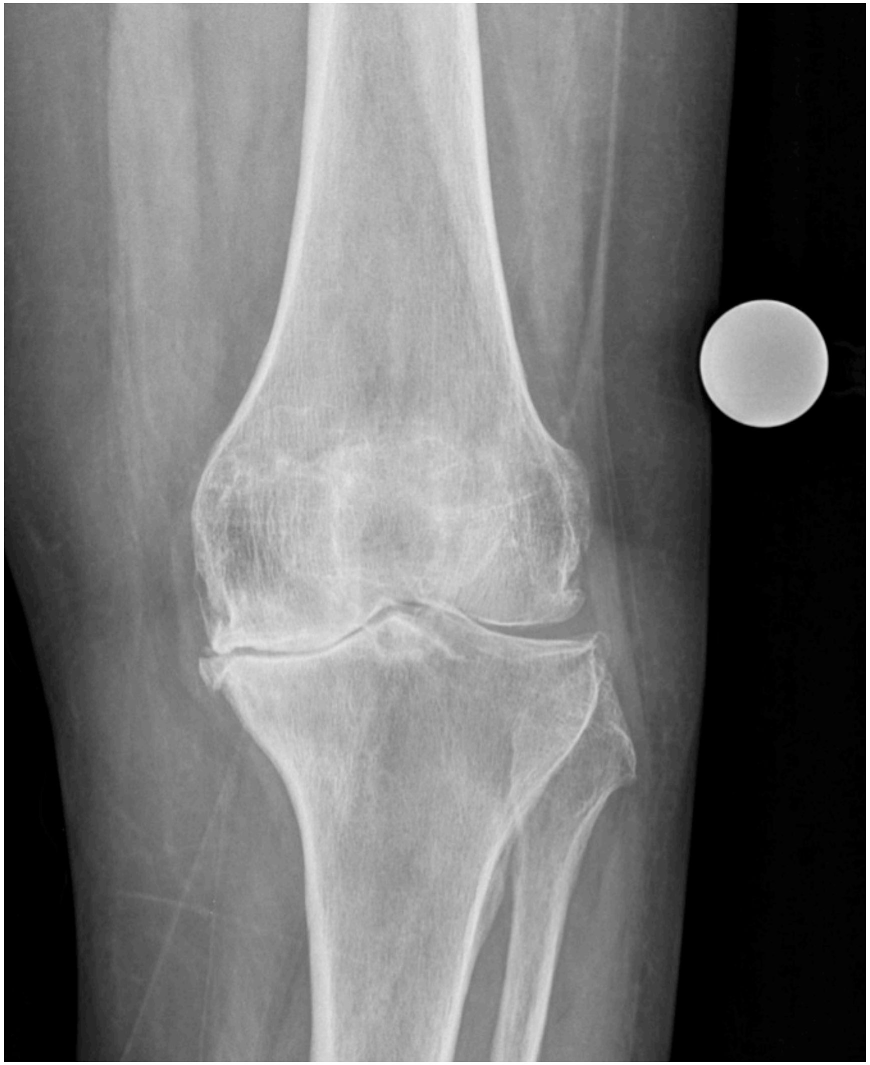

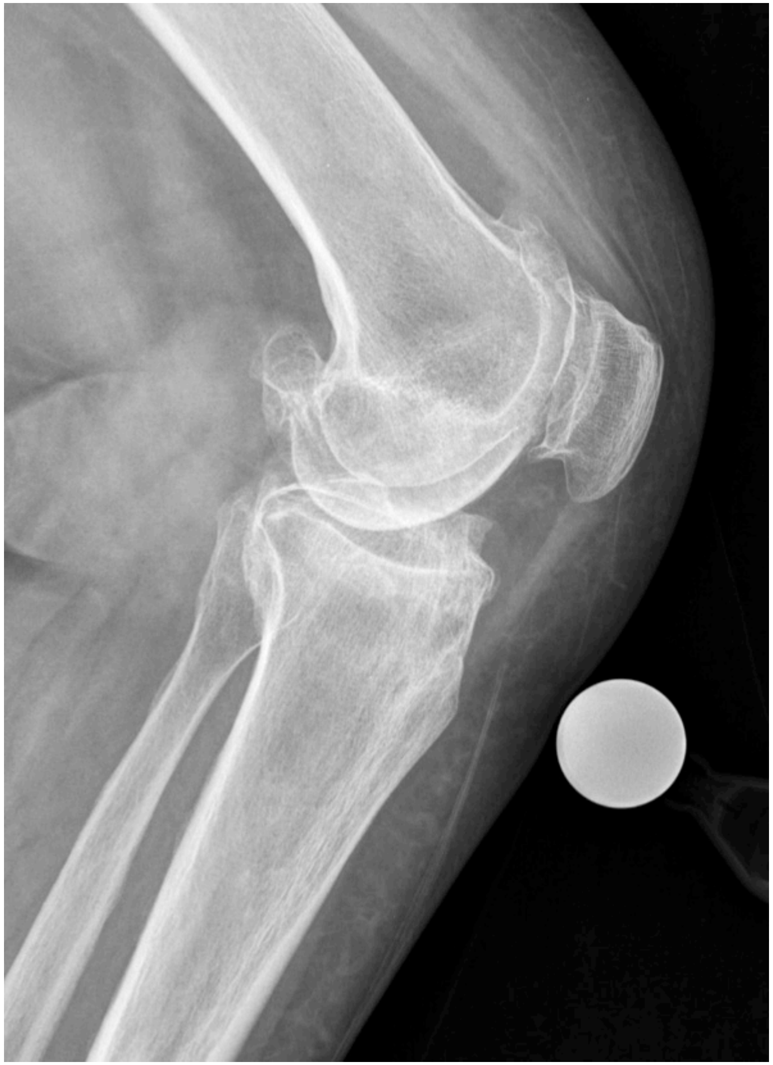

6 weeks postop

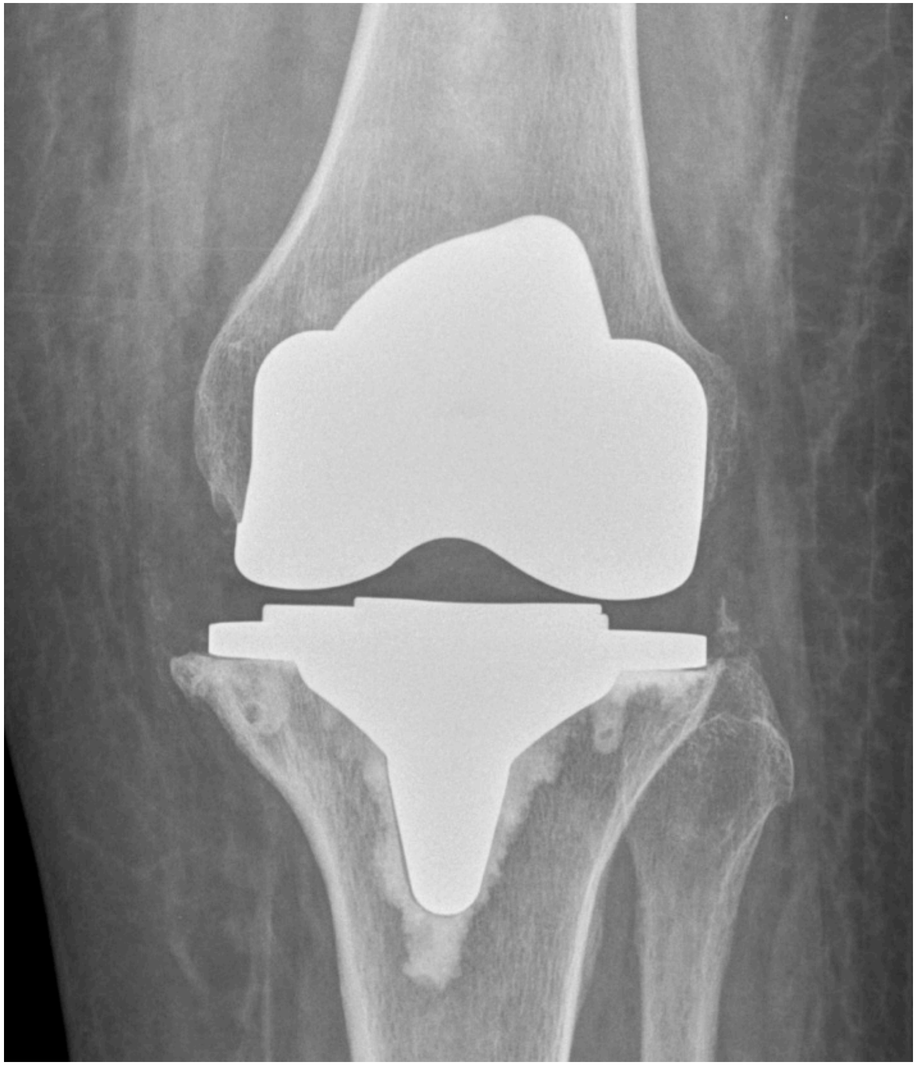

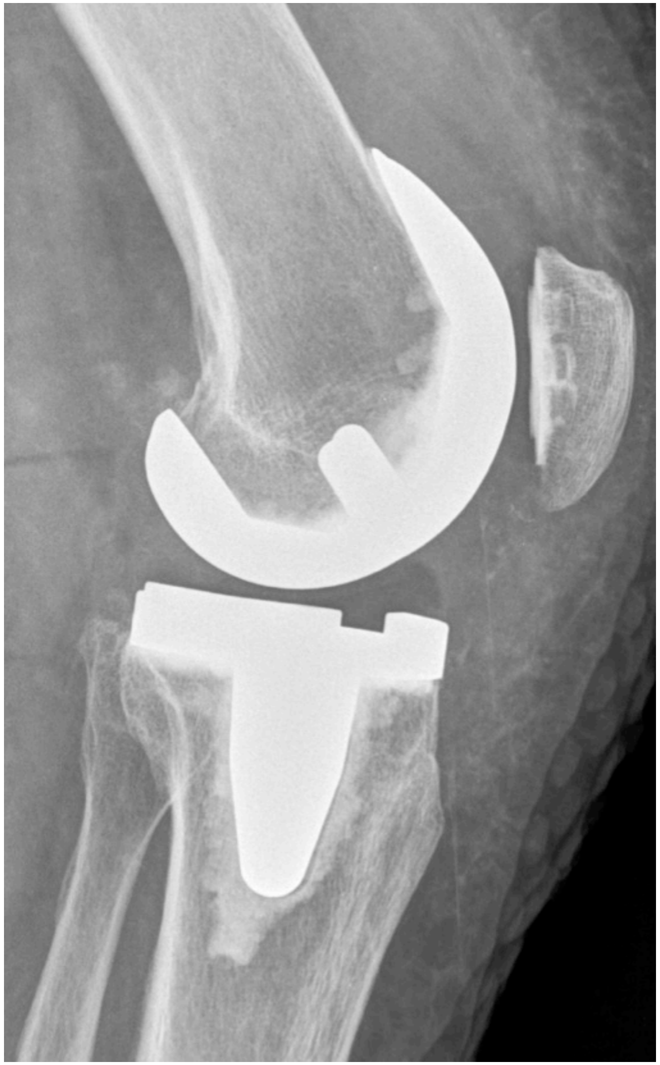

6 months postop

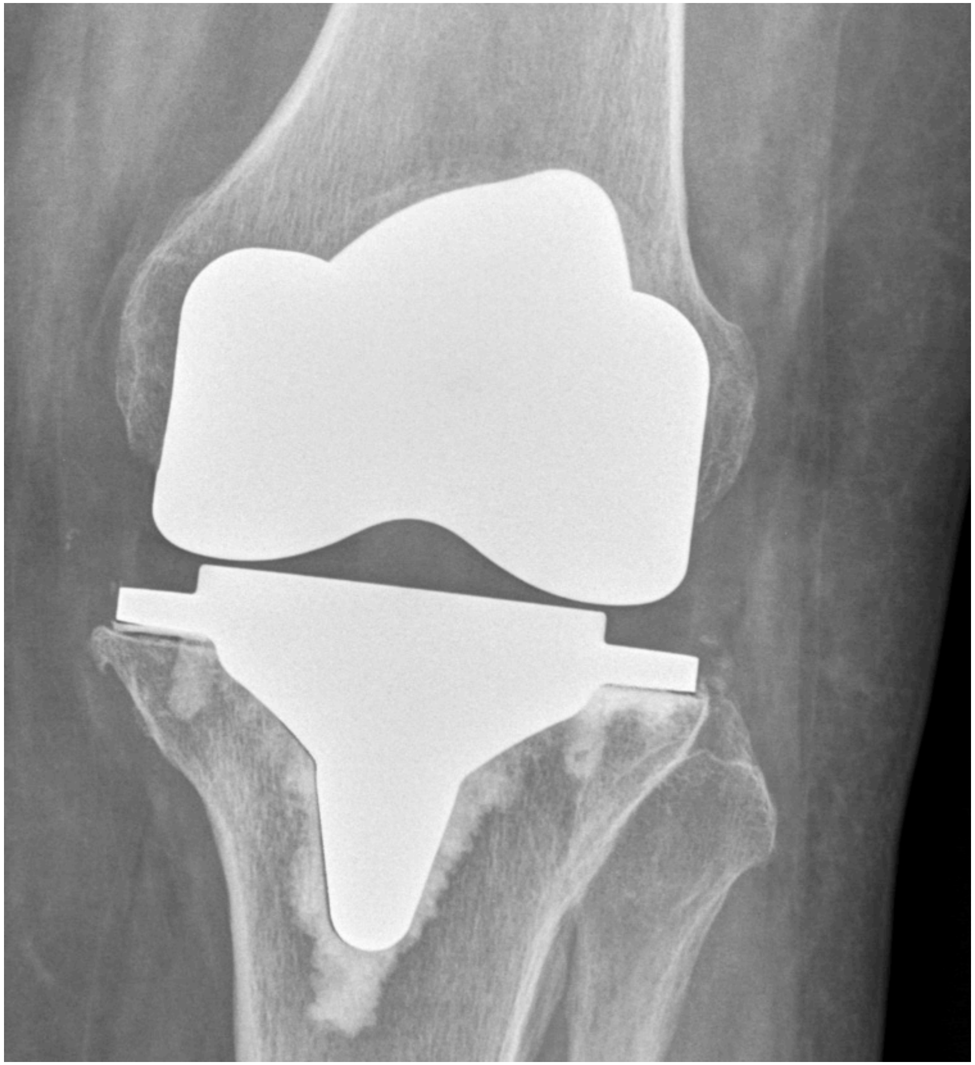

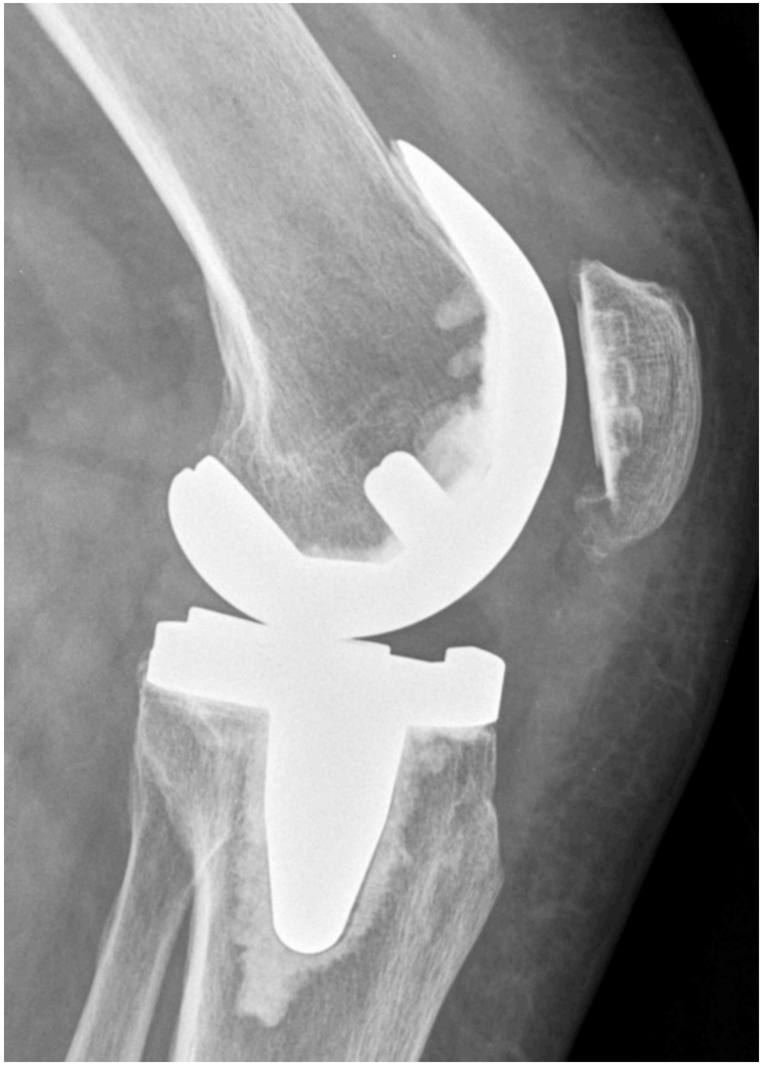

12 months postop

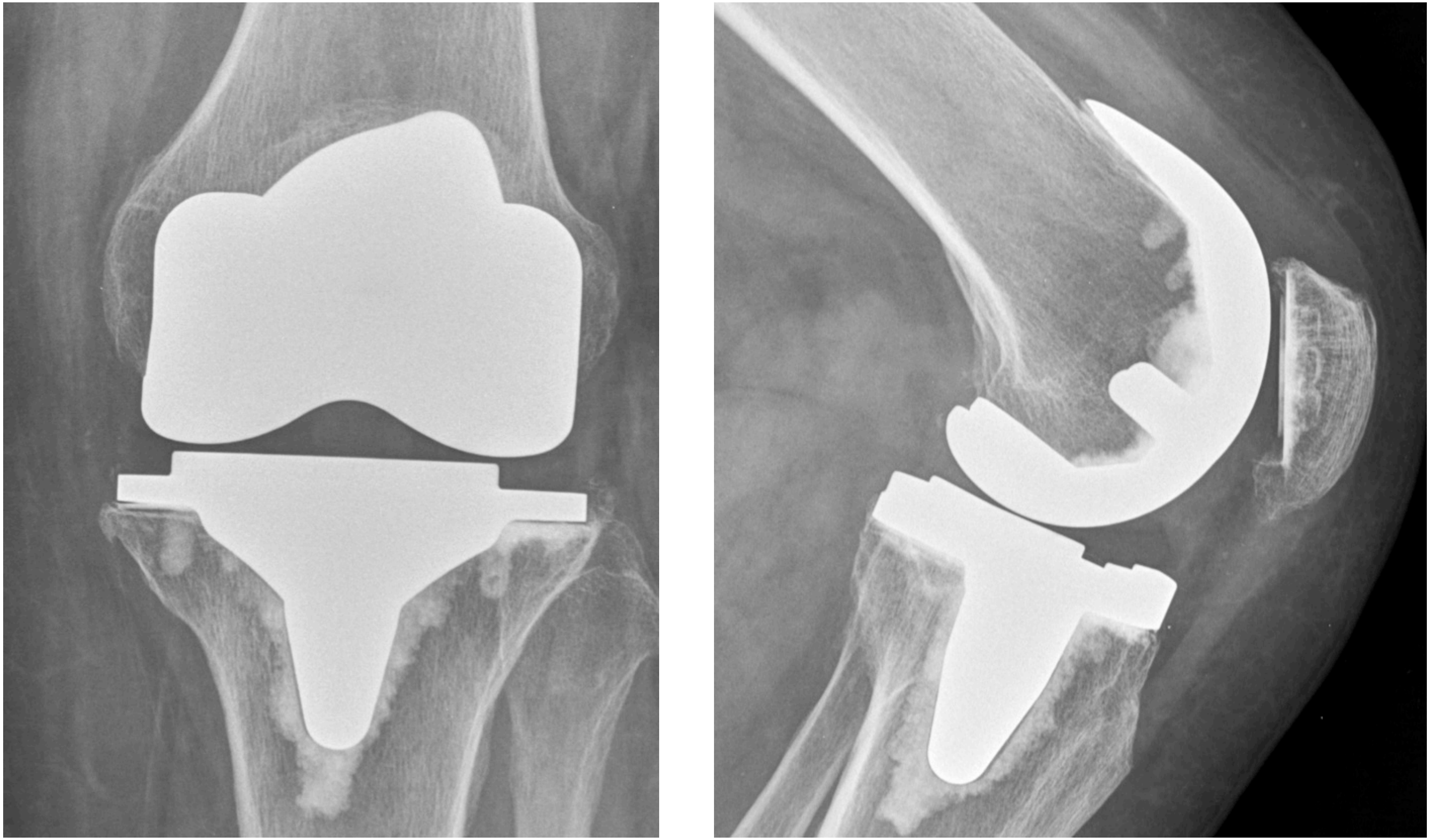

Case 4

preoperative

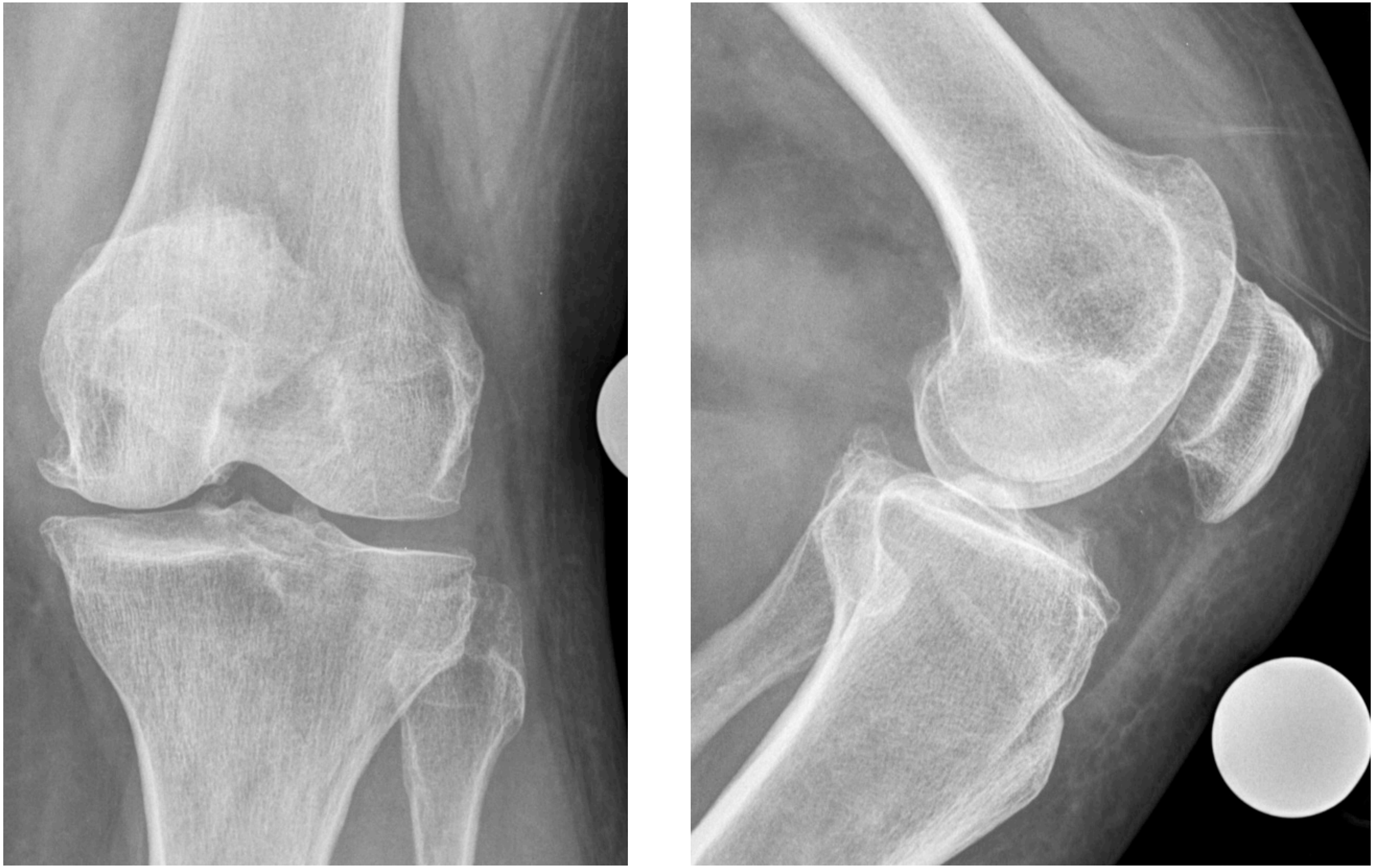

6 weeks postop

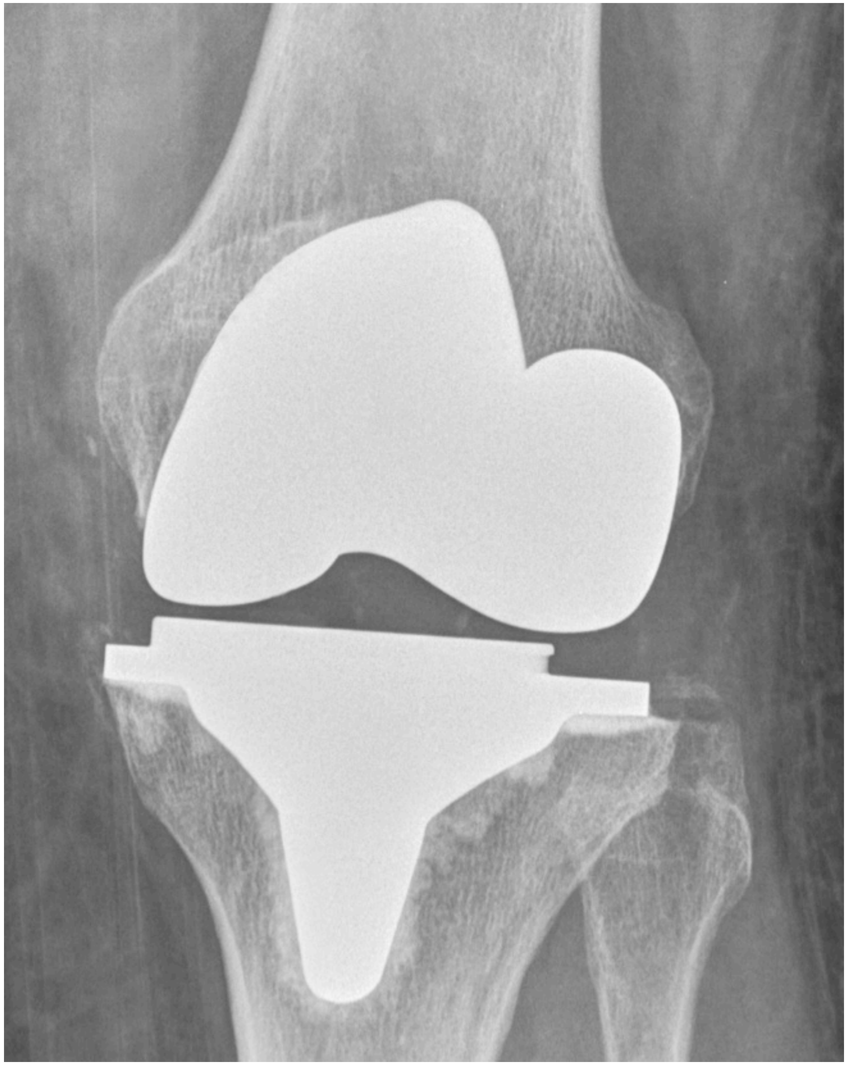

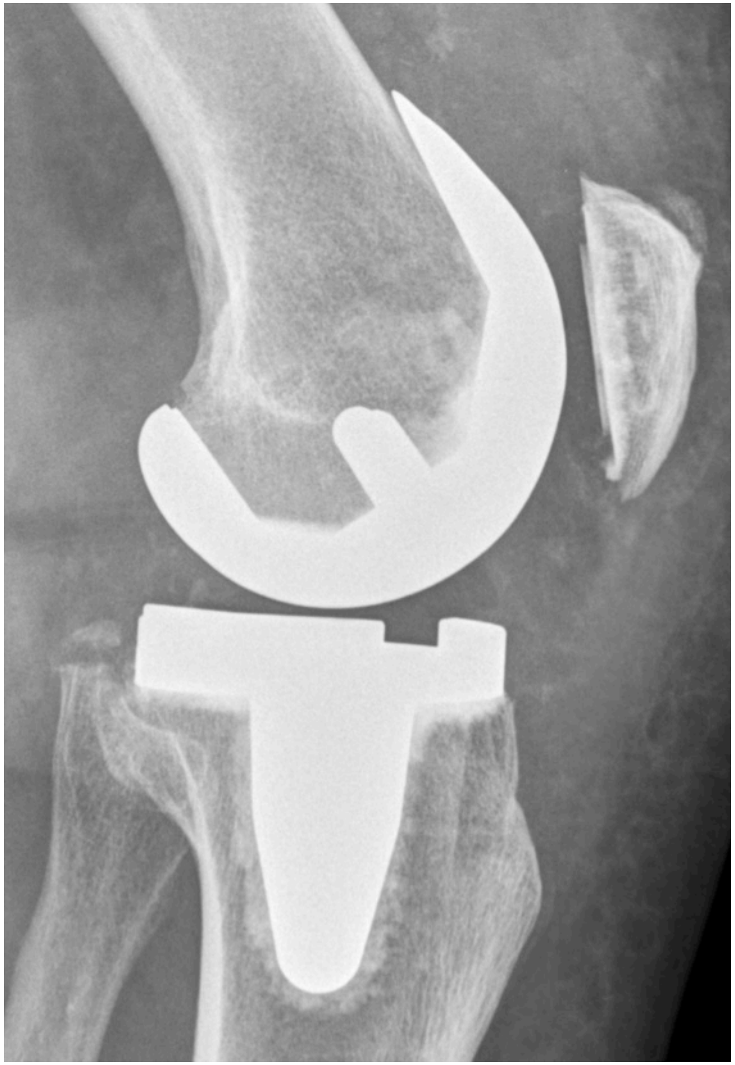

6 months postop

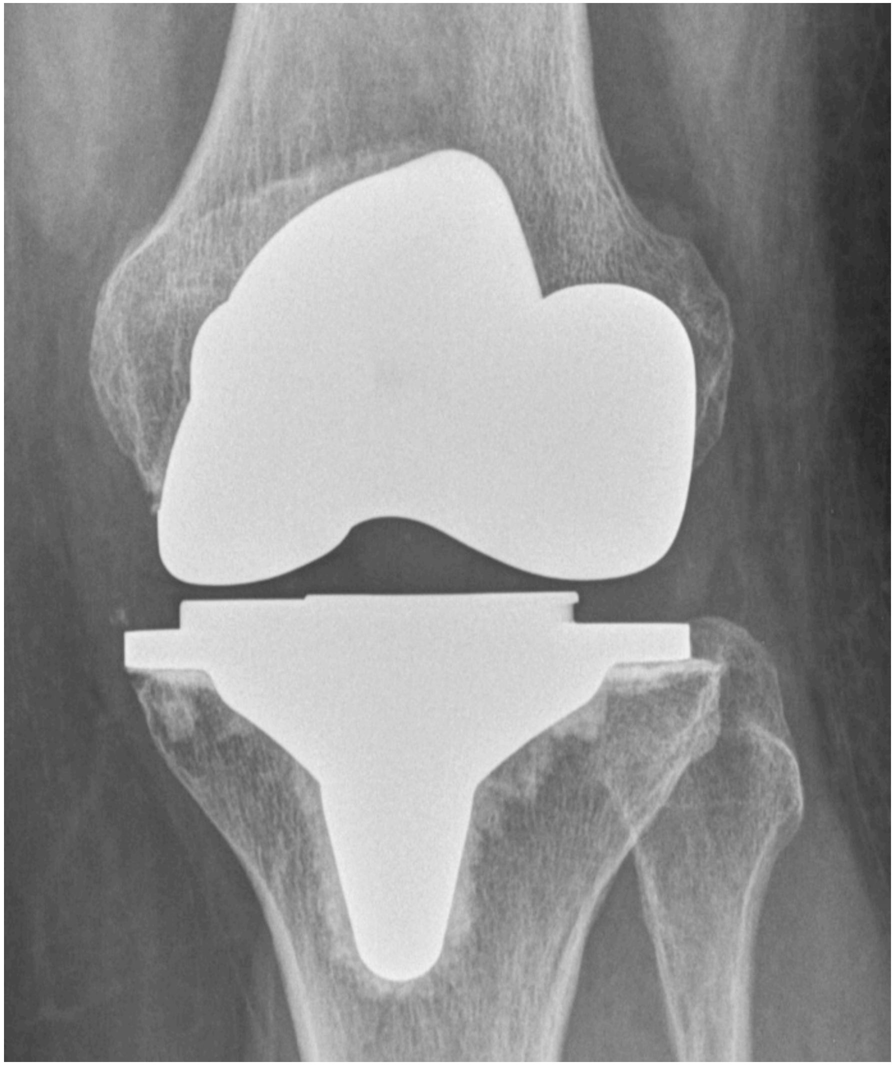

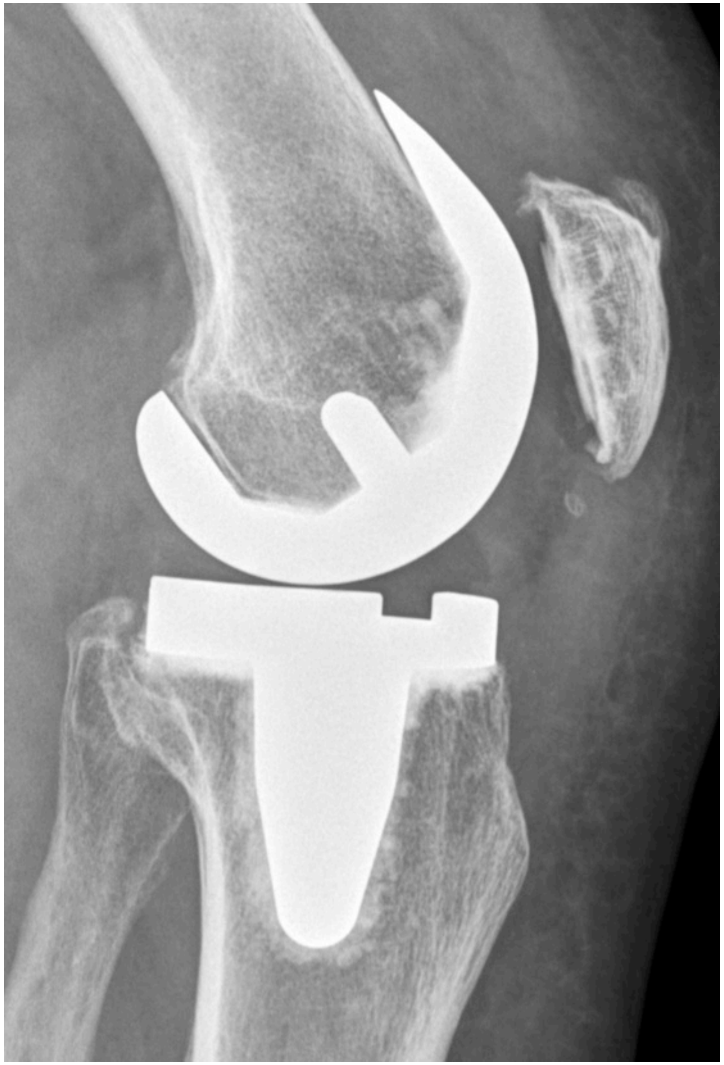

12 months postop

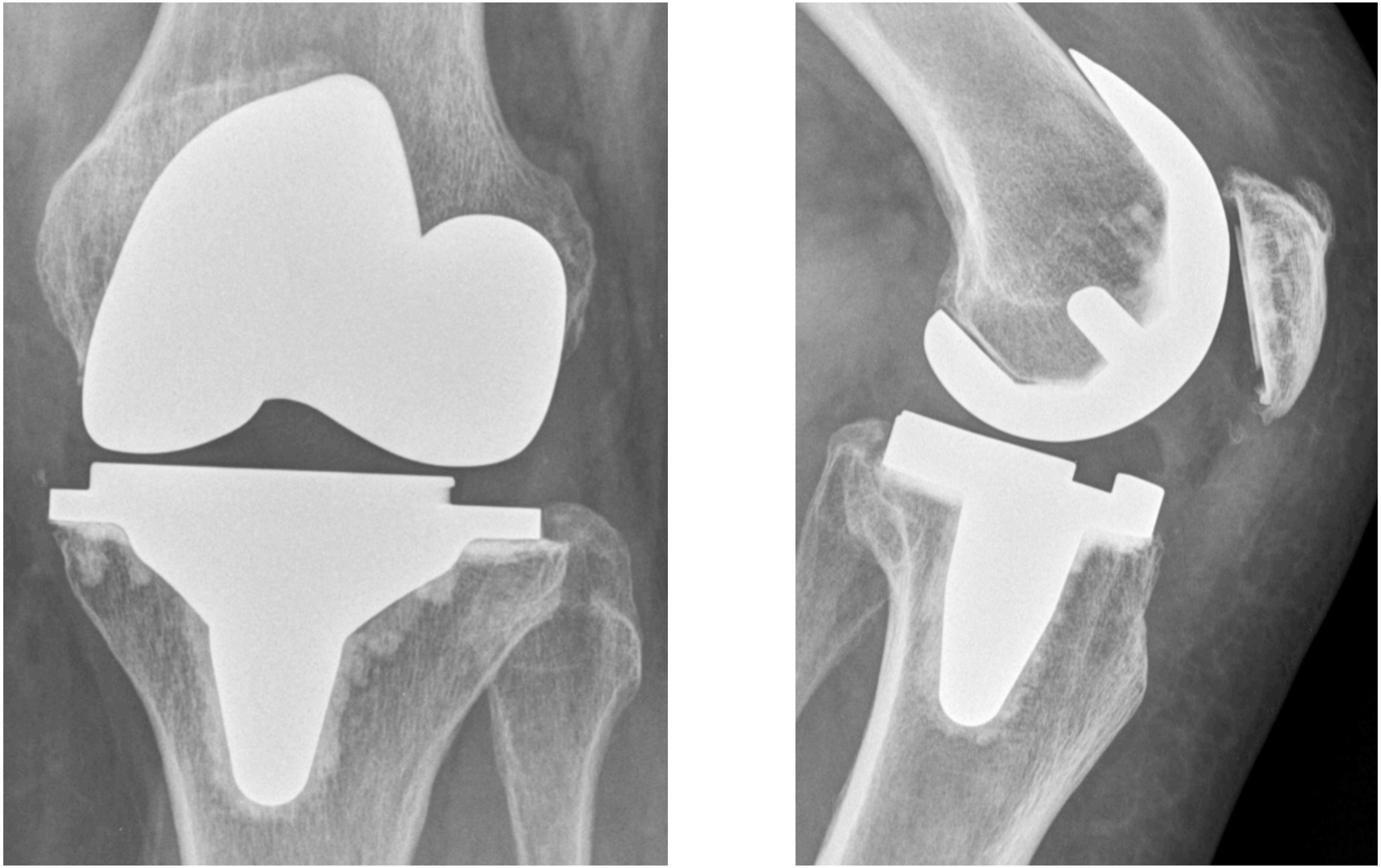

Case 5

preoperative

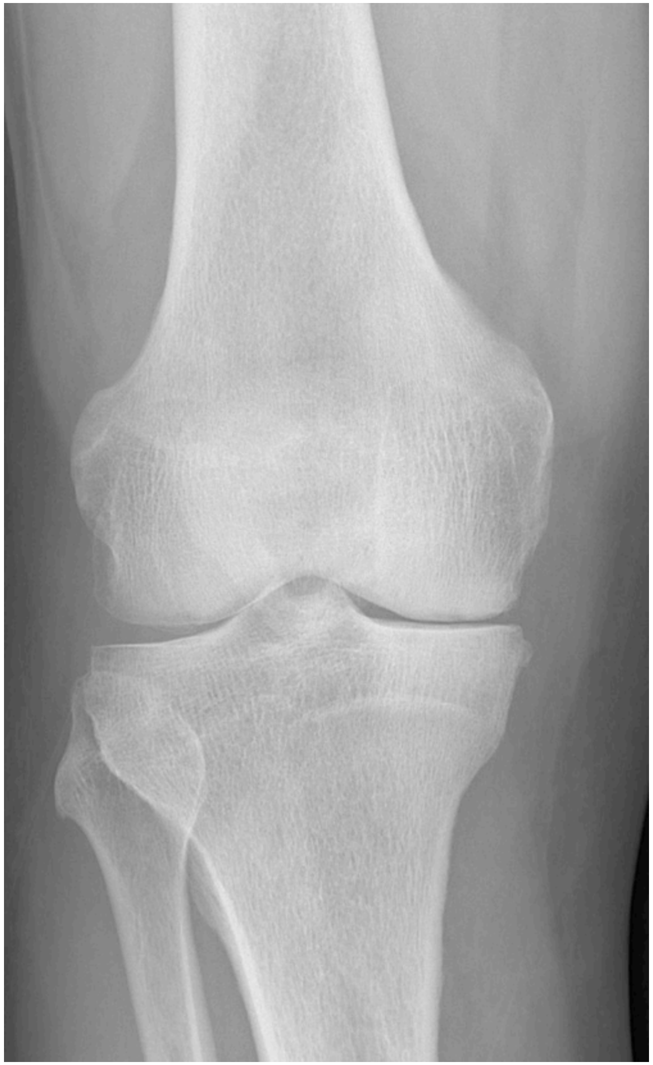

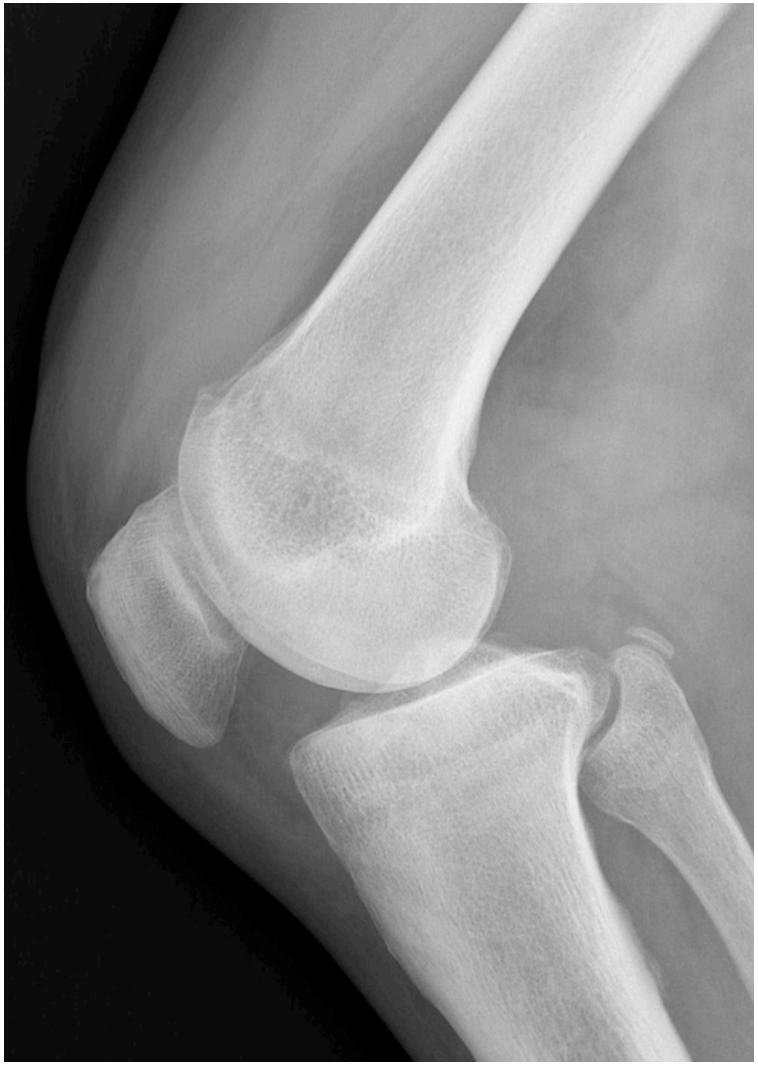

6 weeks postop

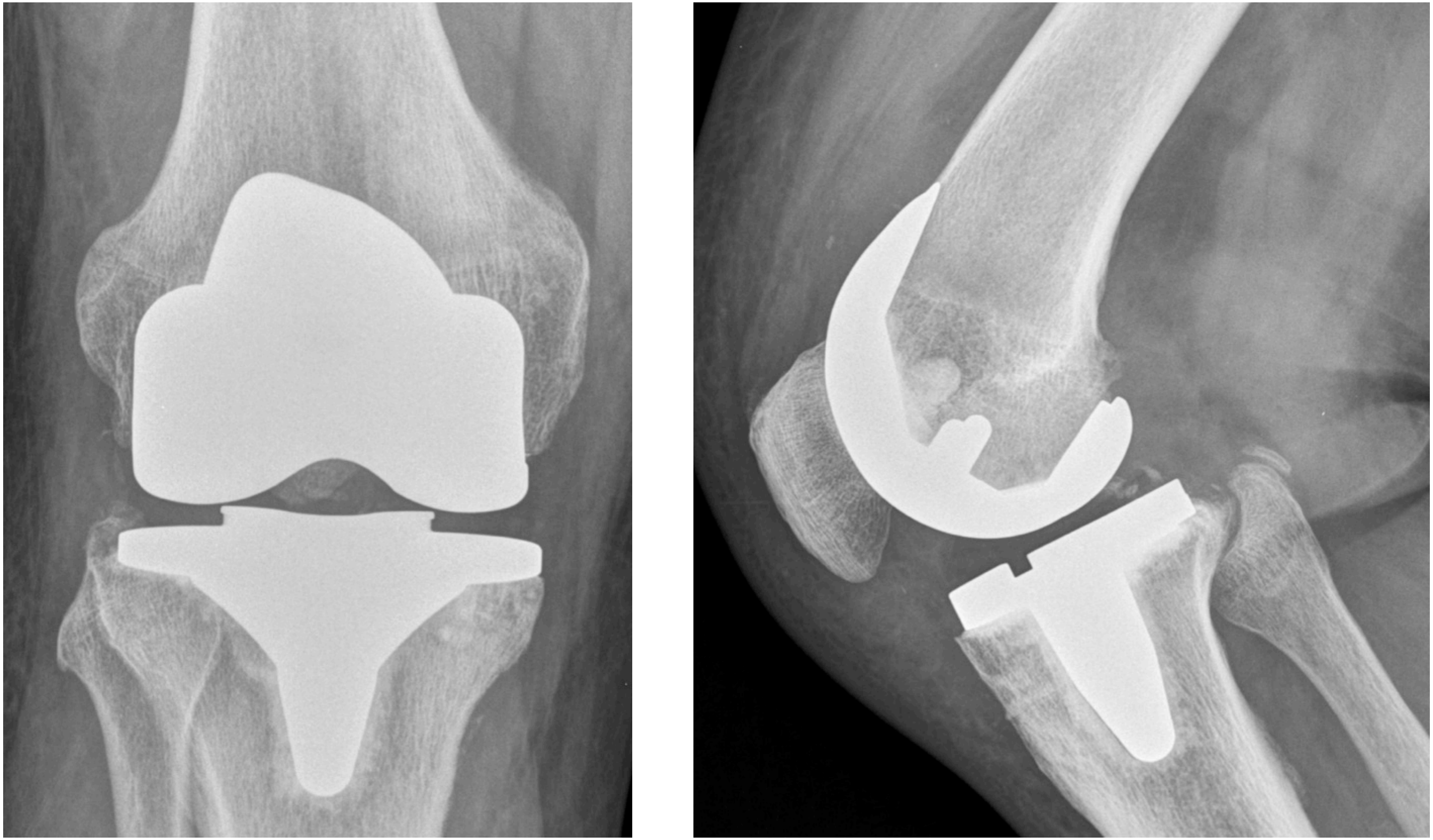

6 months postop

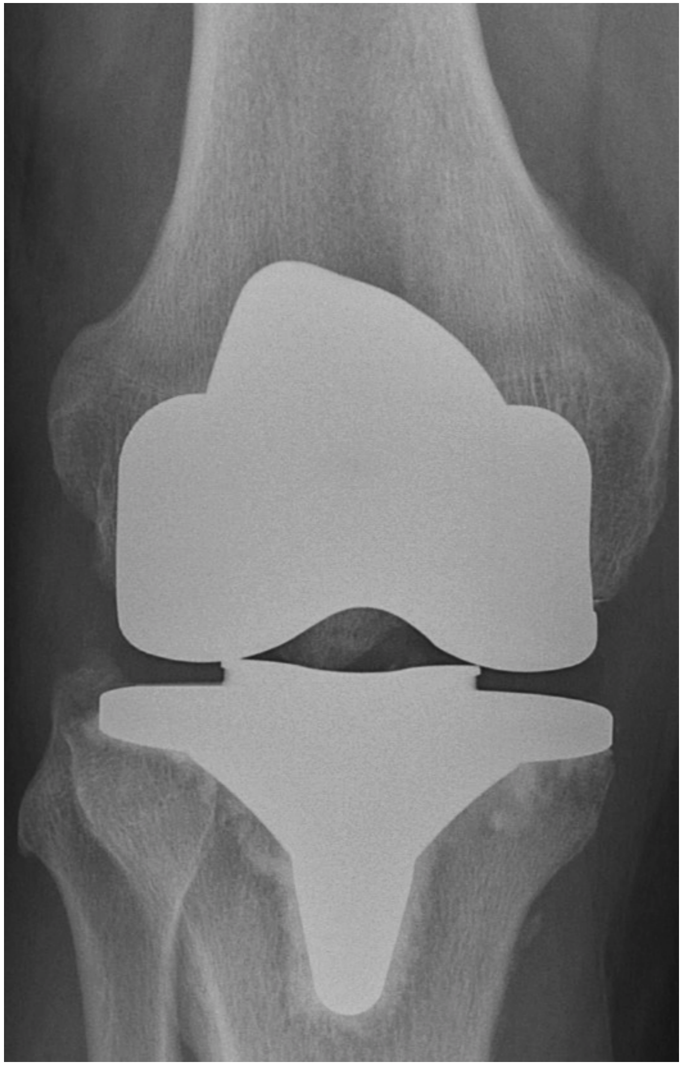

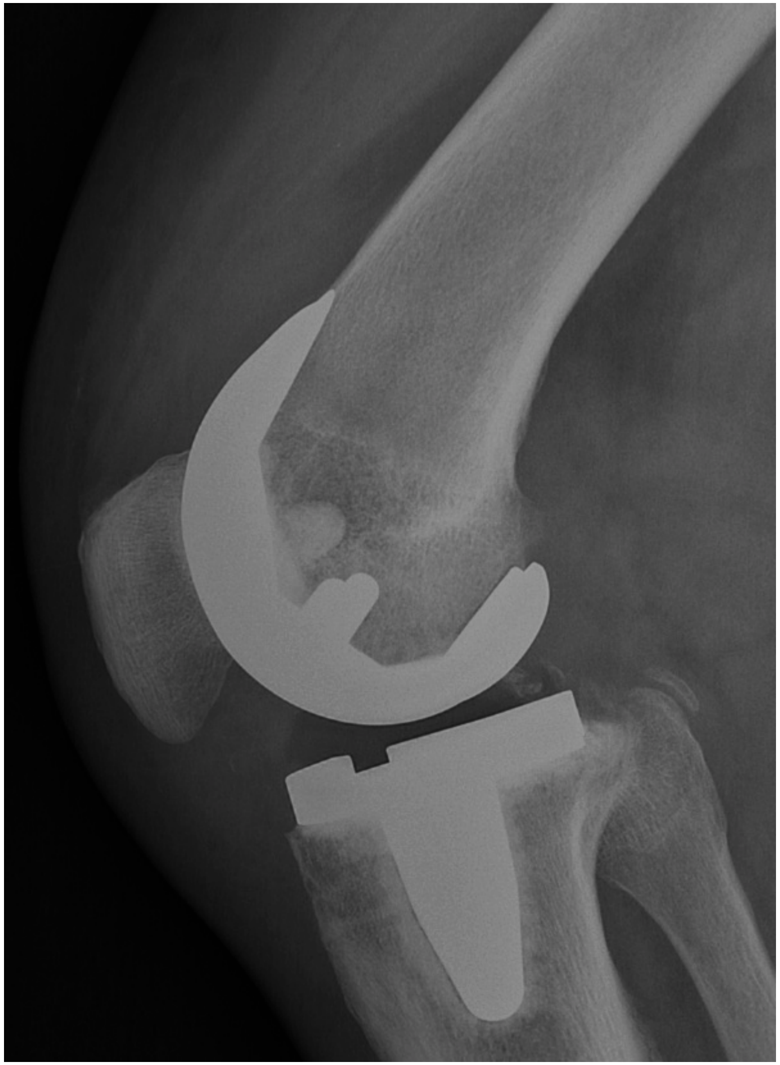

12 months postop

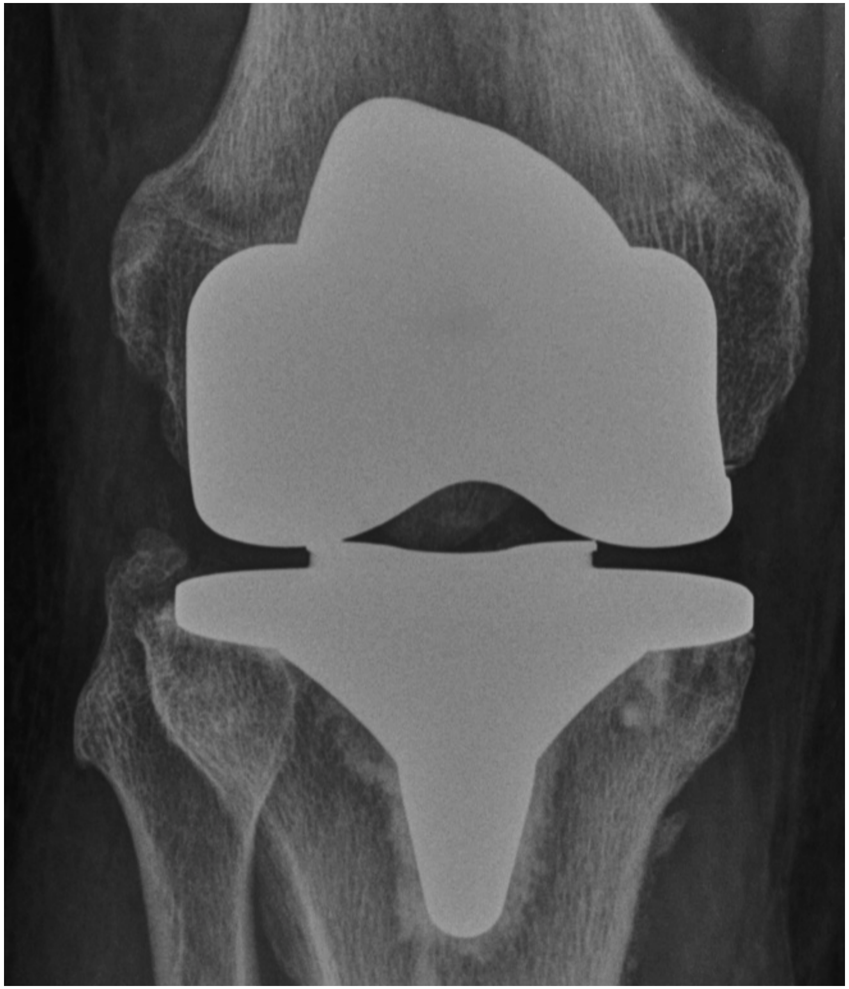

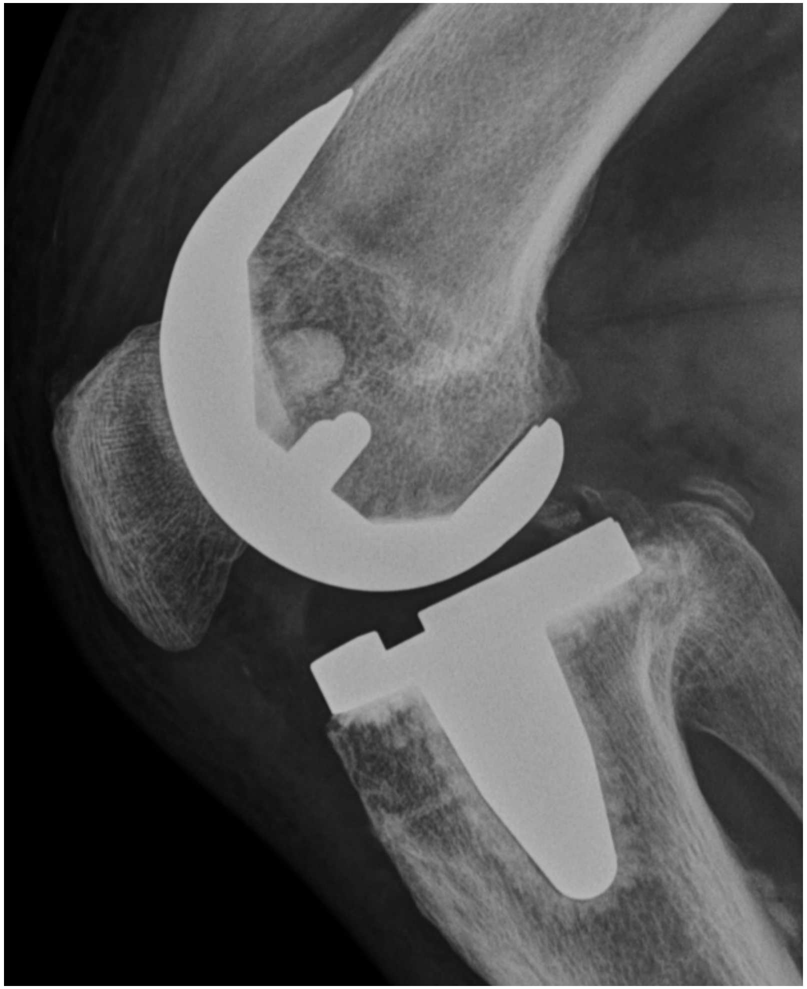

Case 6

preoperative

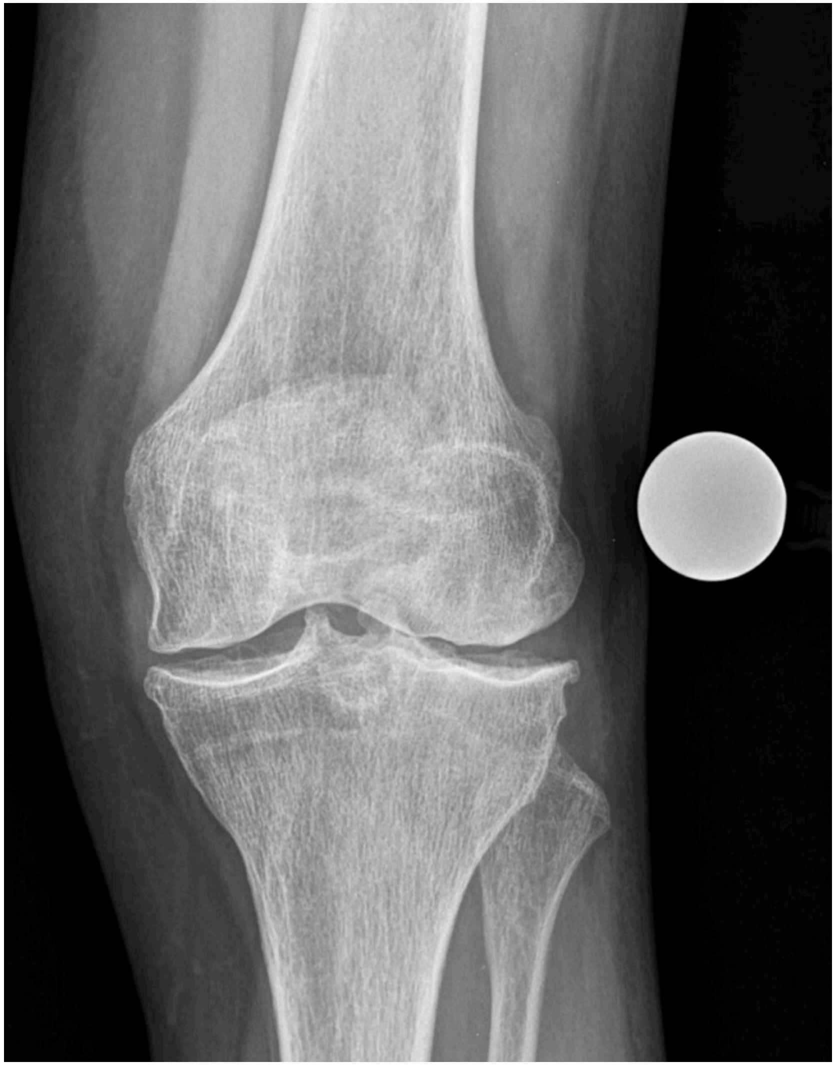

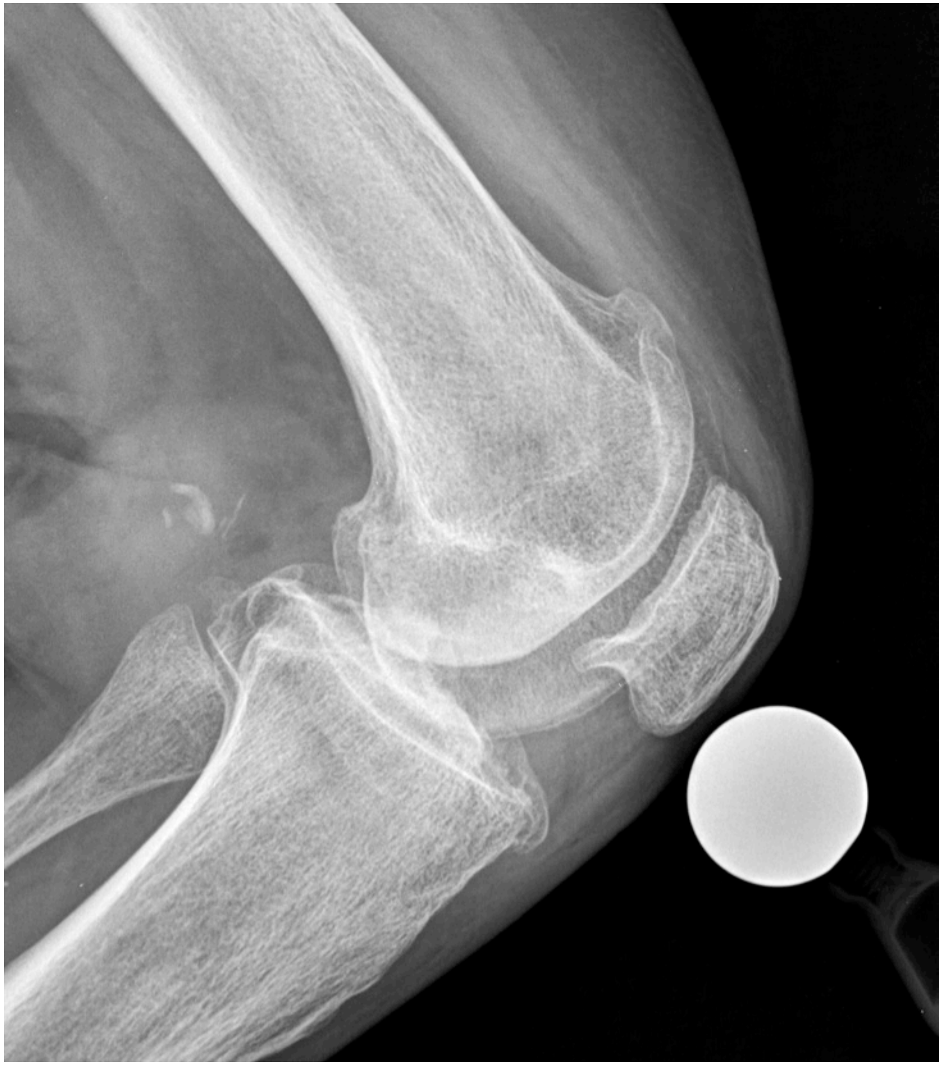

6 weeks postop

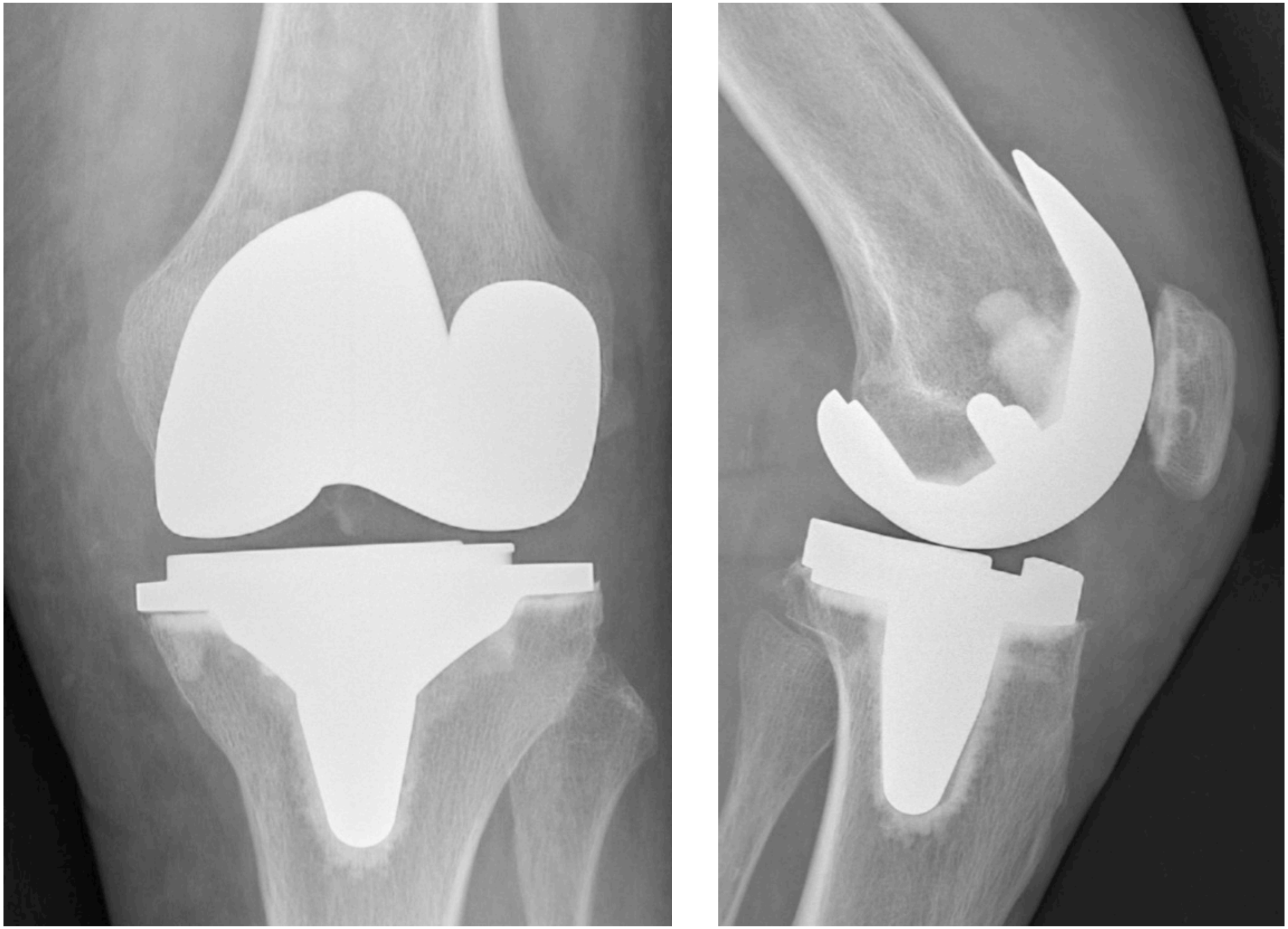

6 months postop

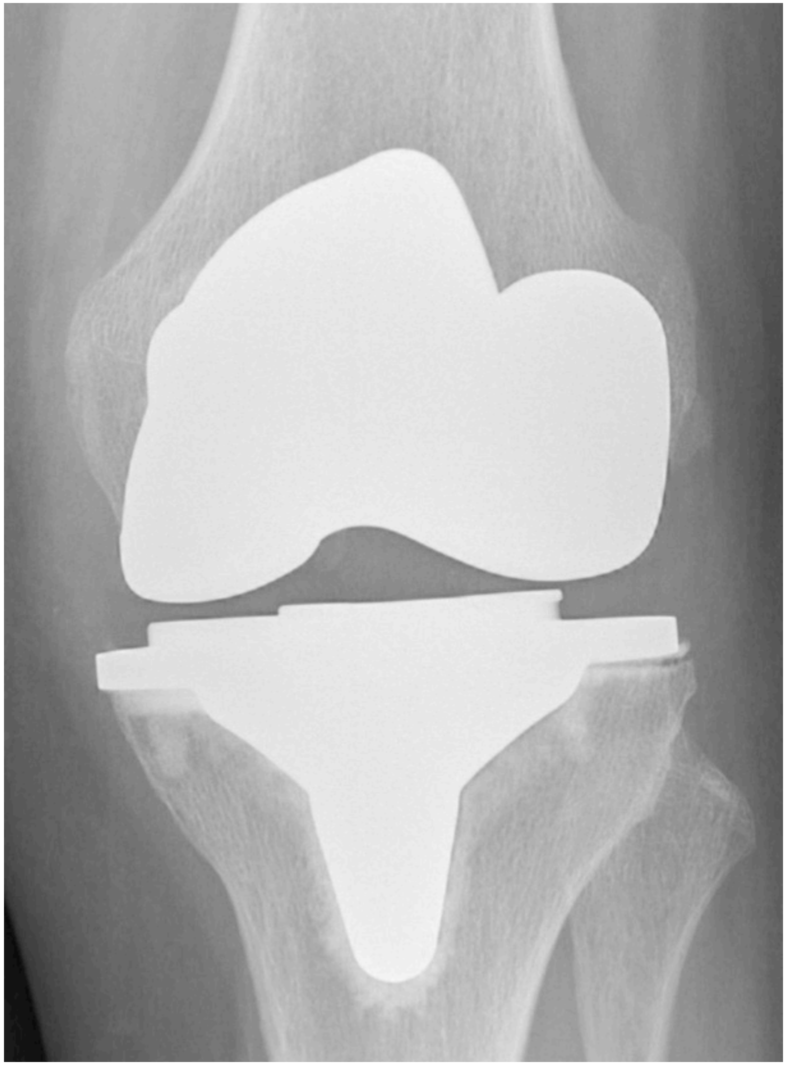

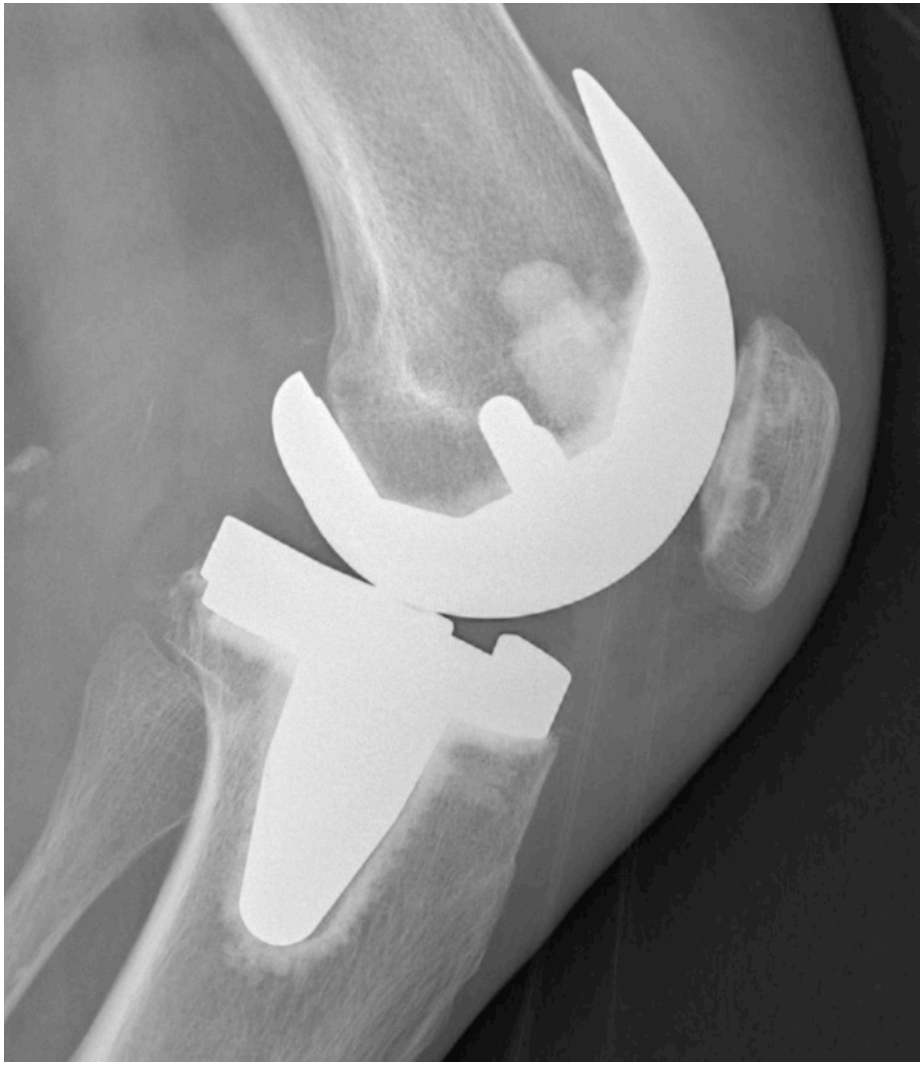

12 months postop

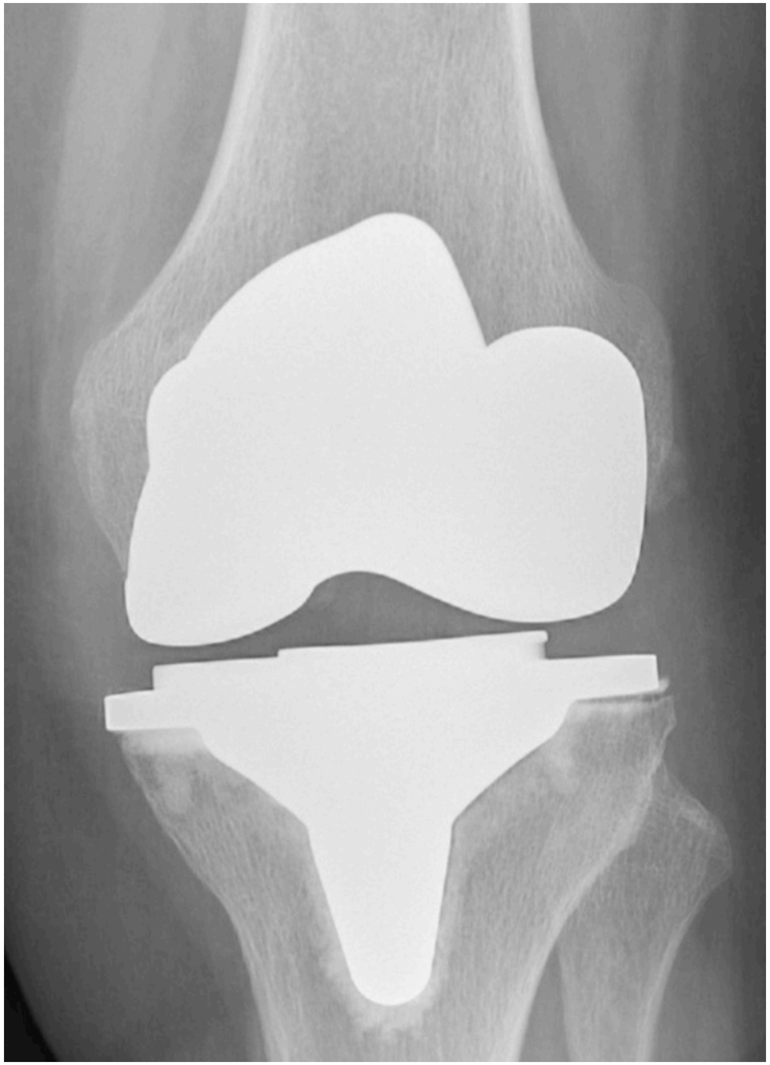

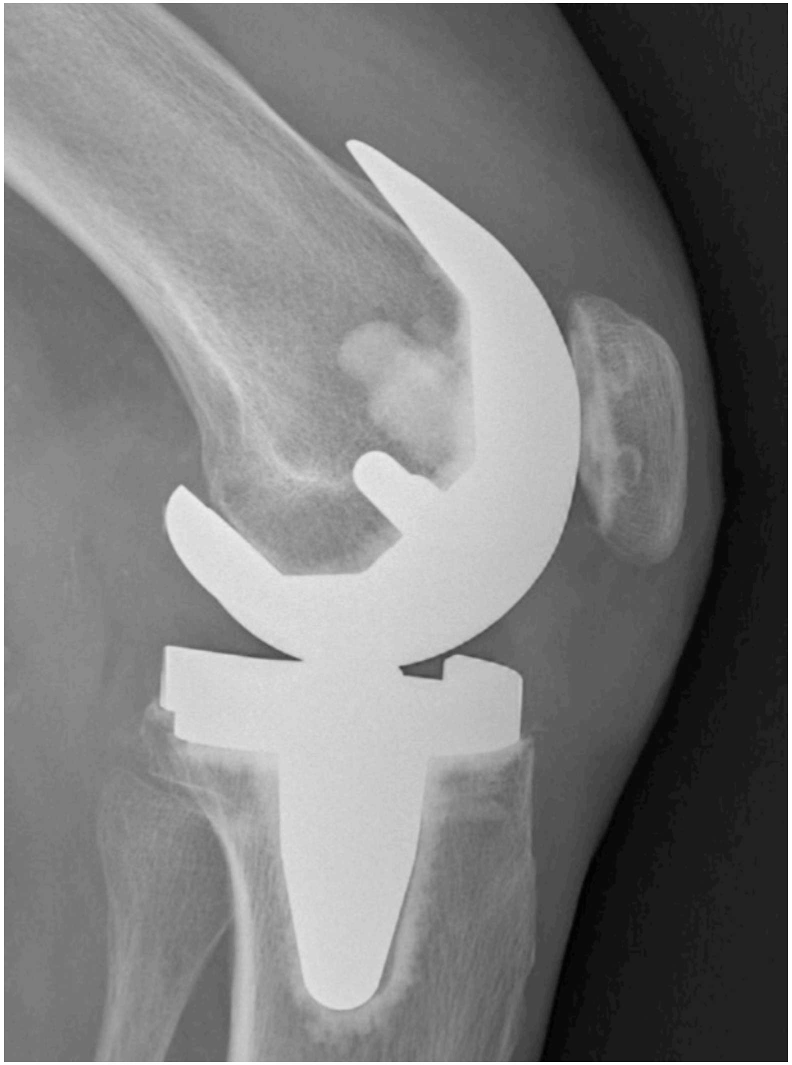

Case 7

preoperative

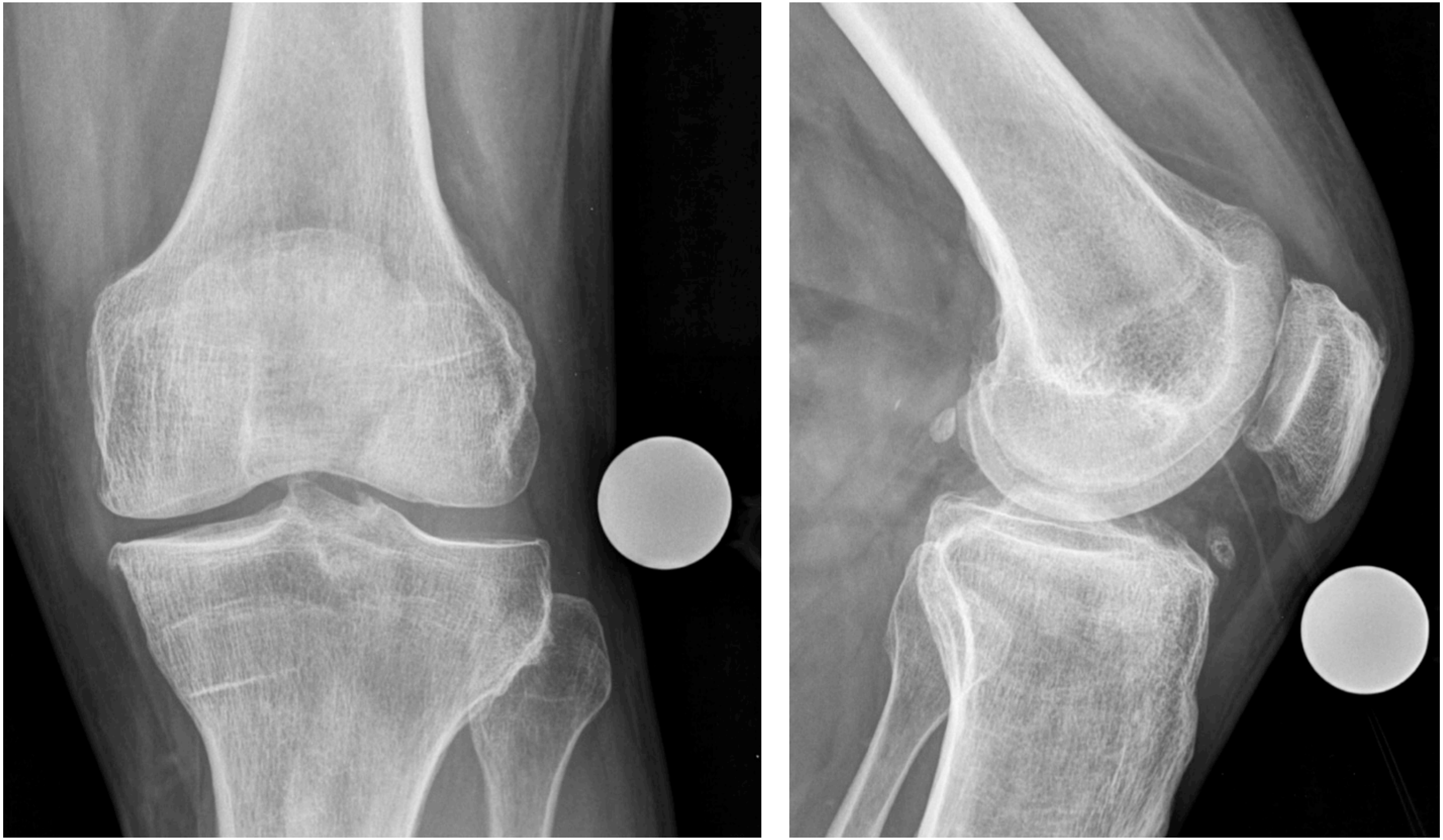

6 weeks postop

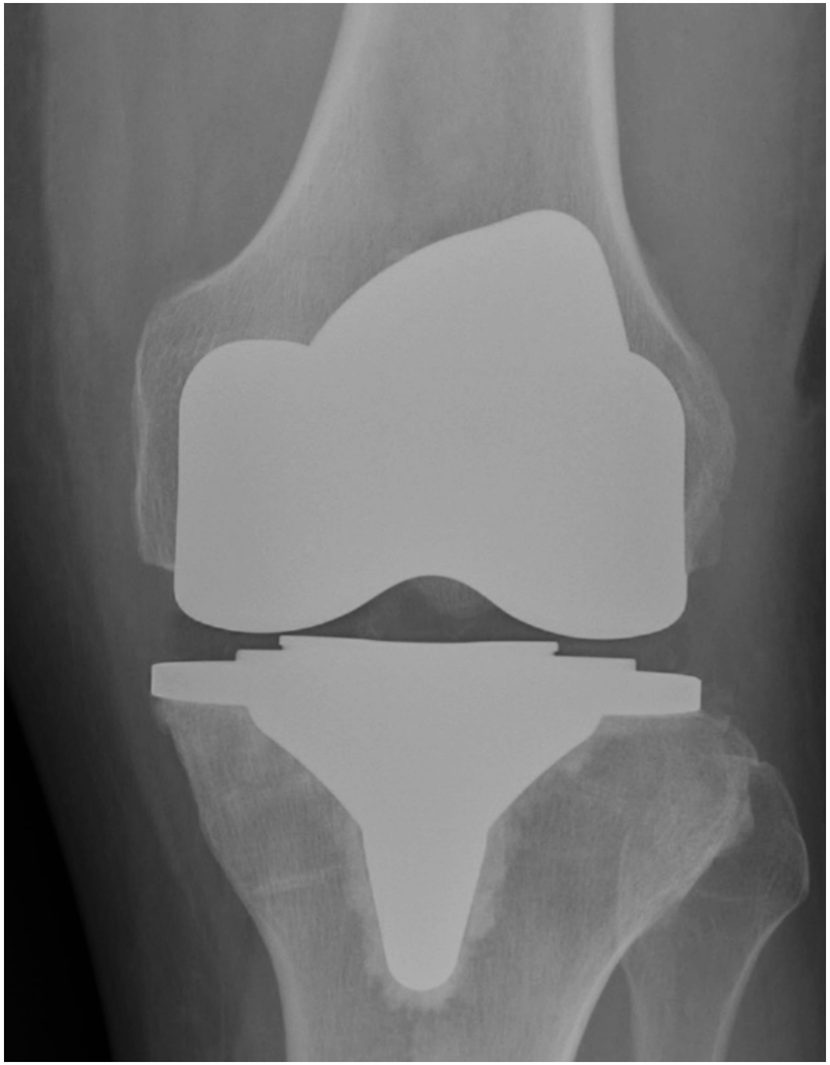

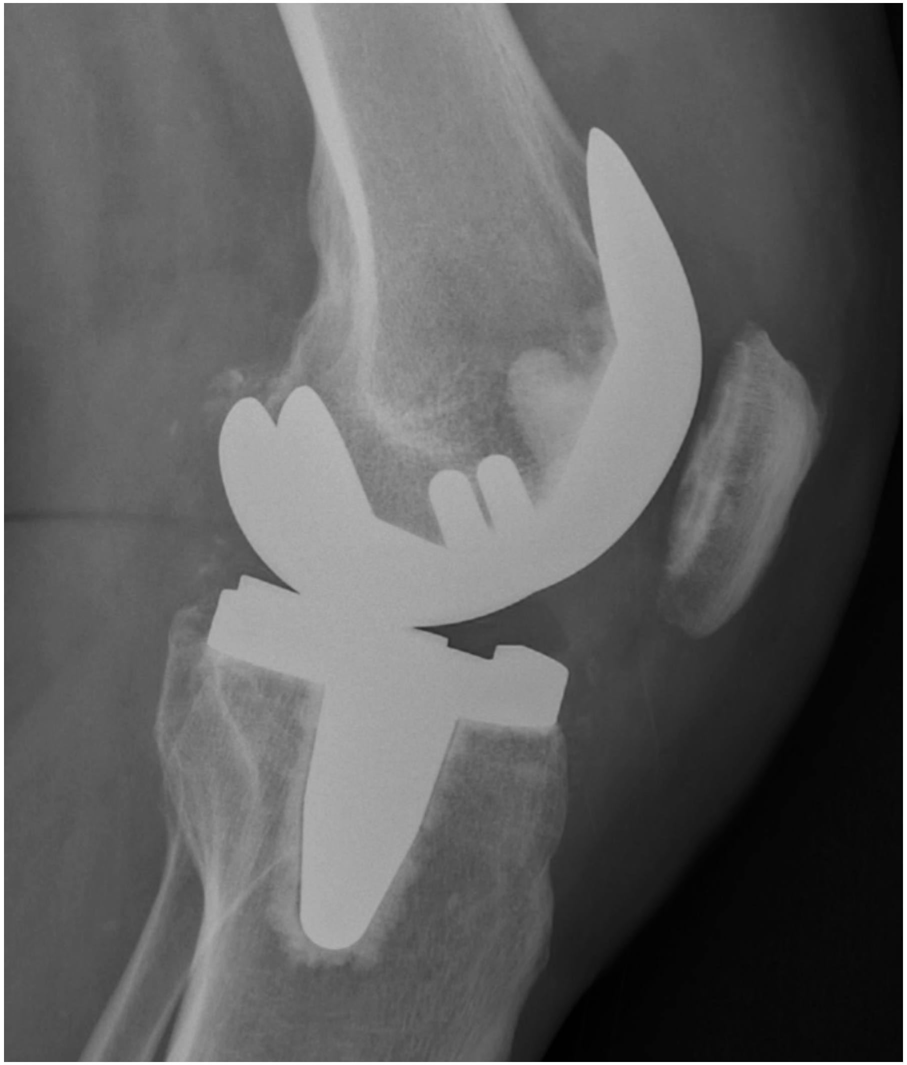

6 months postop

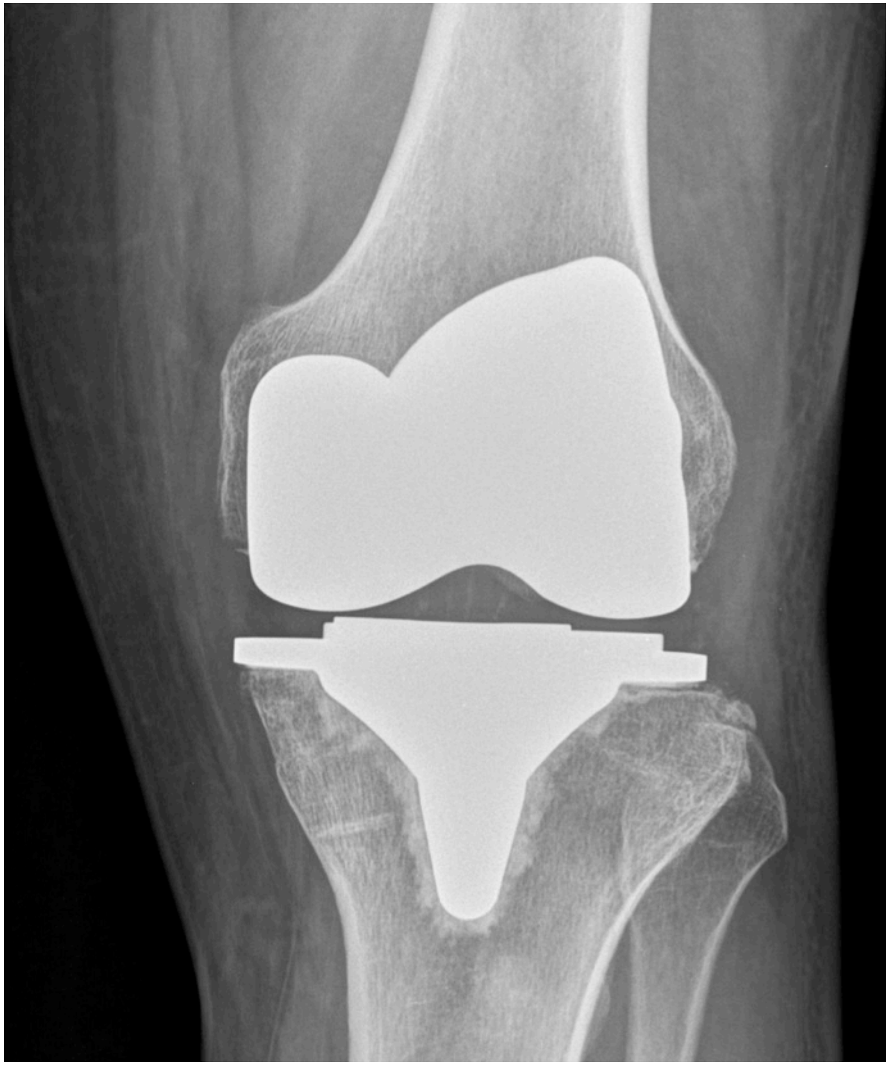

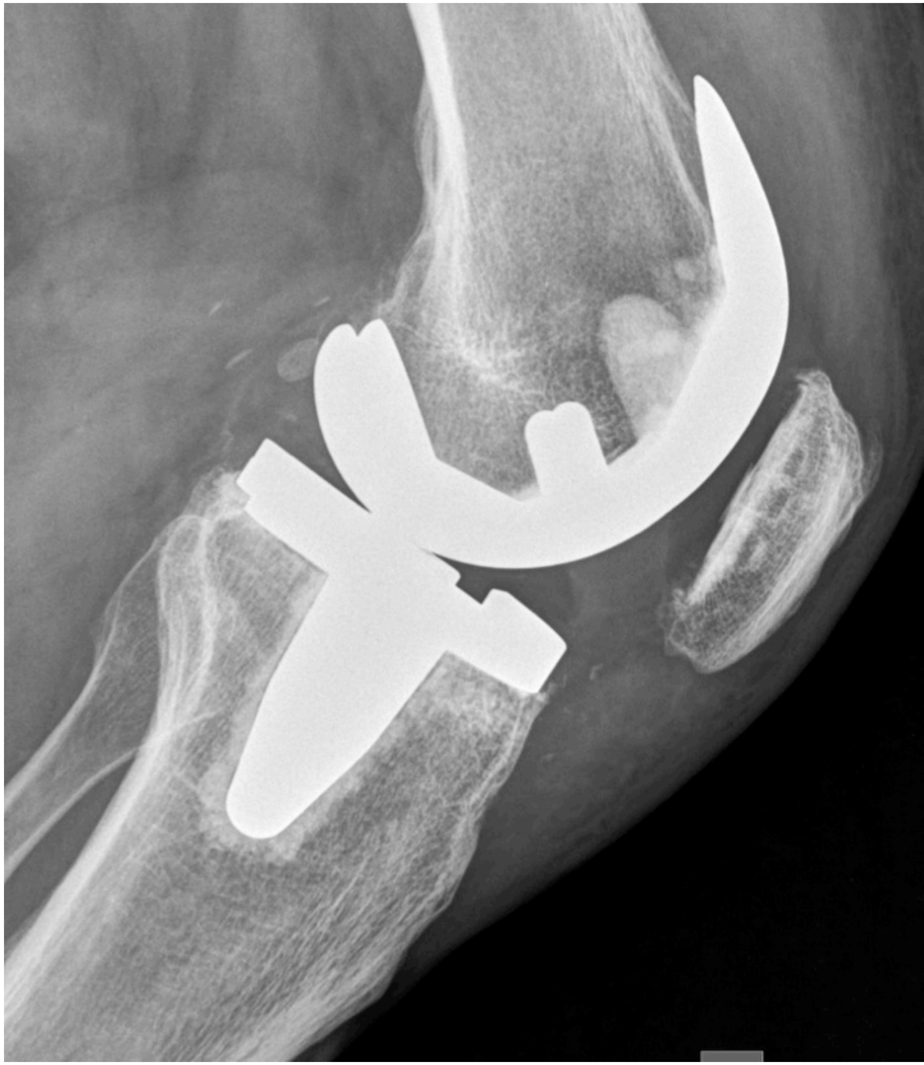

12 months postop

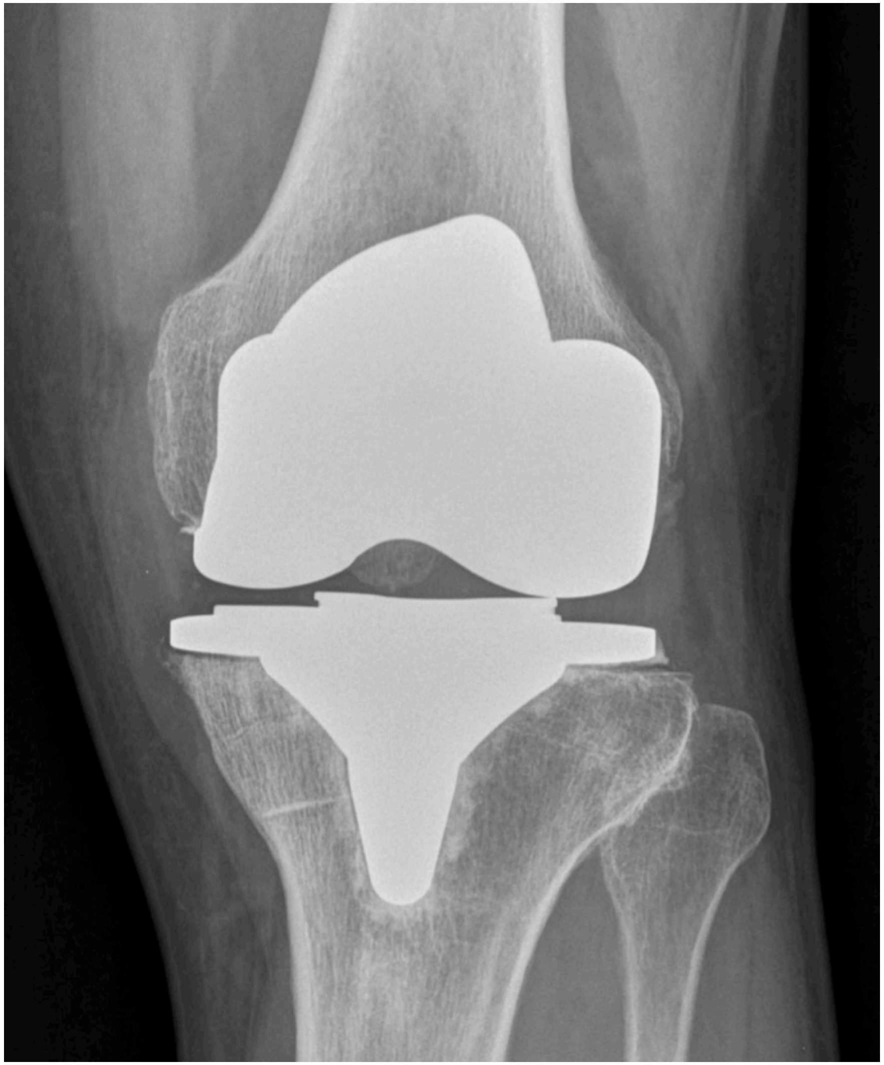

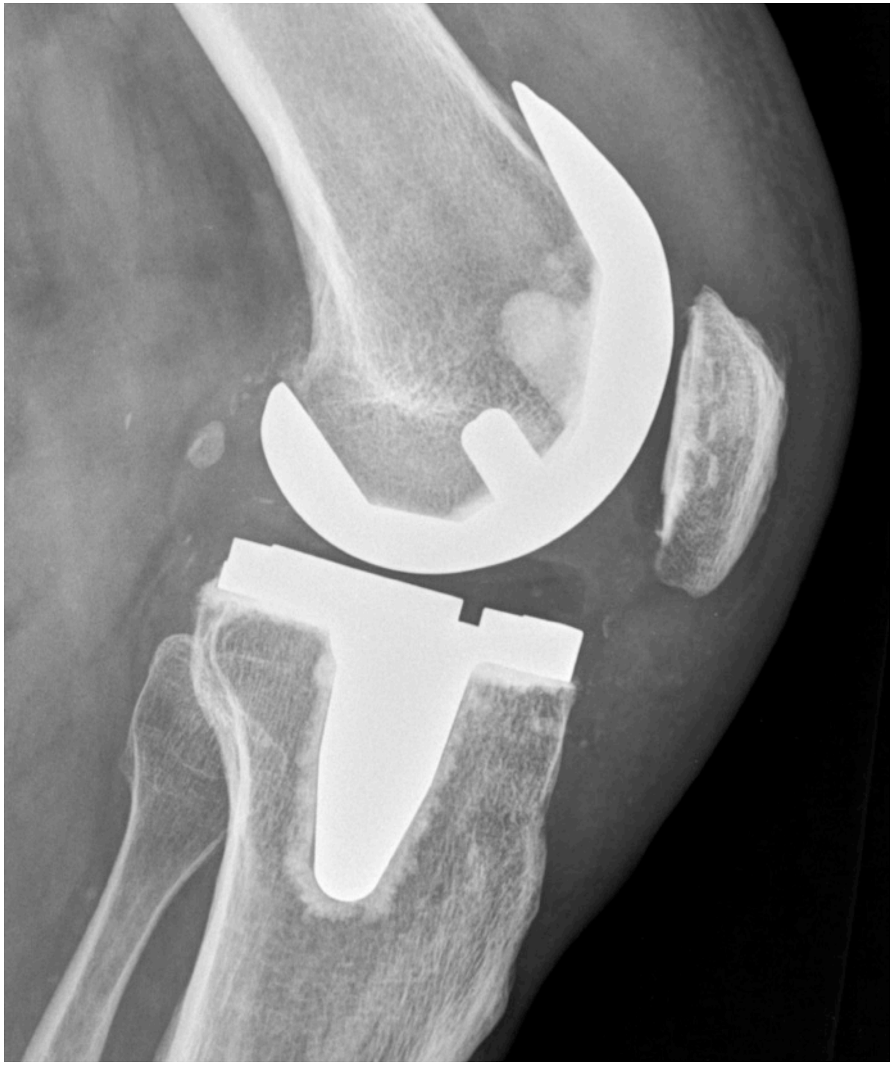

Case 8

preoperative

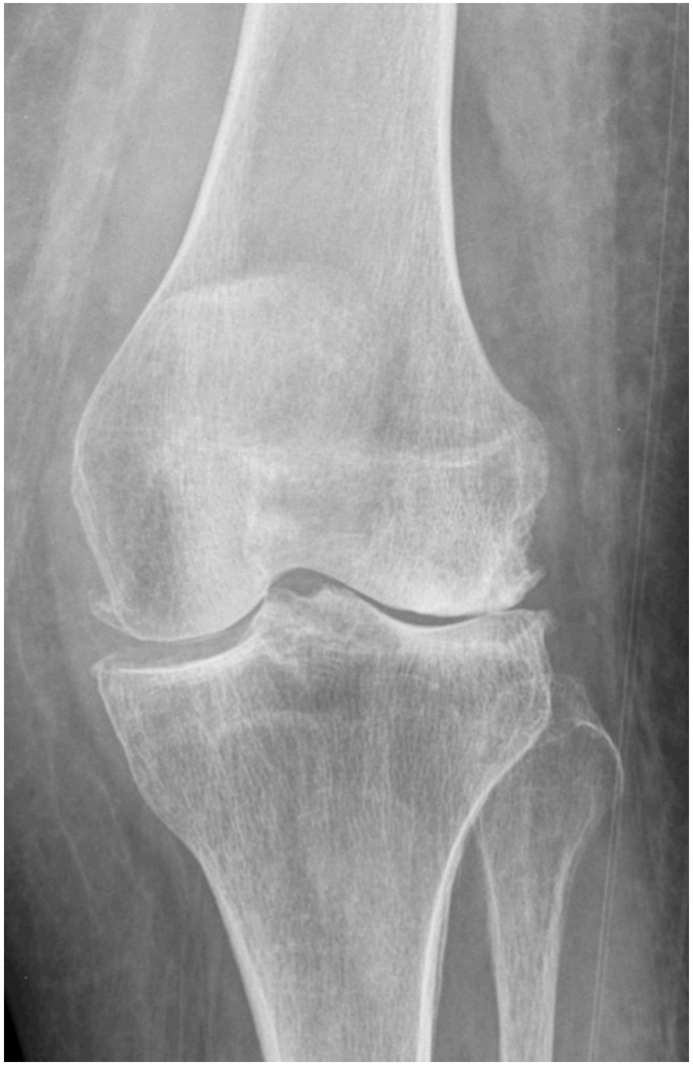

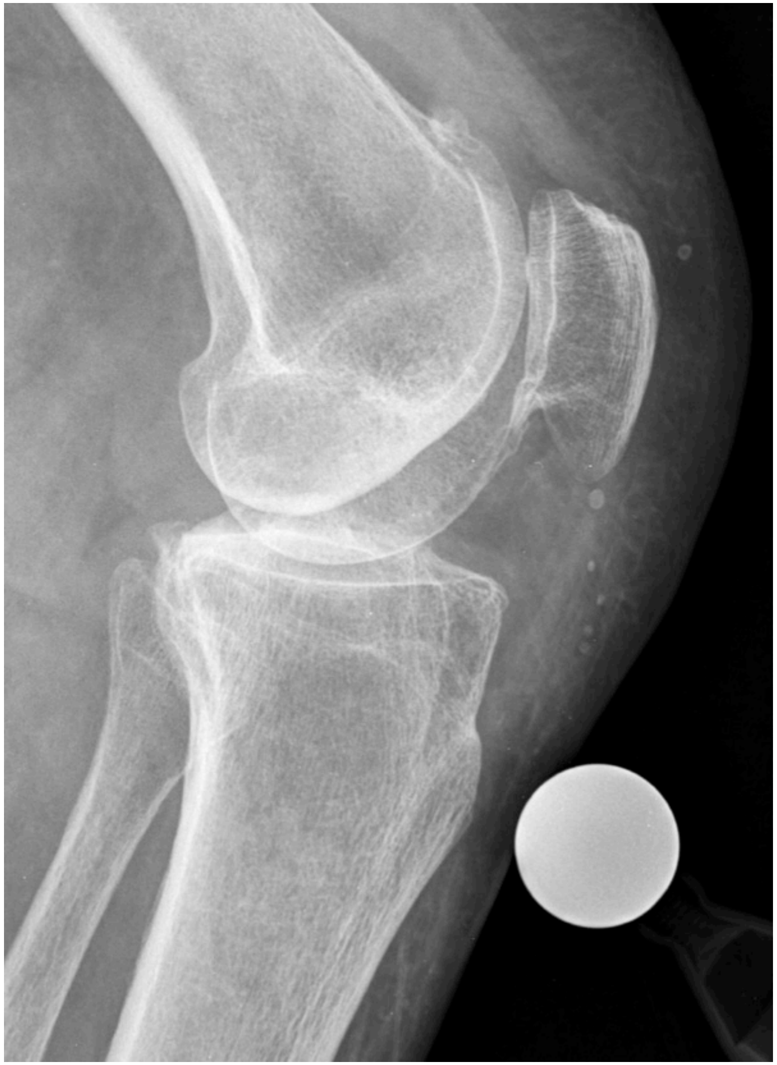

6 weeks postop

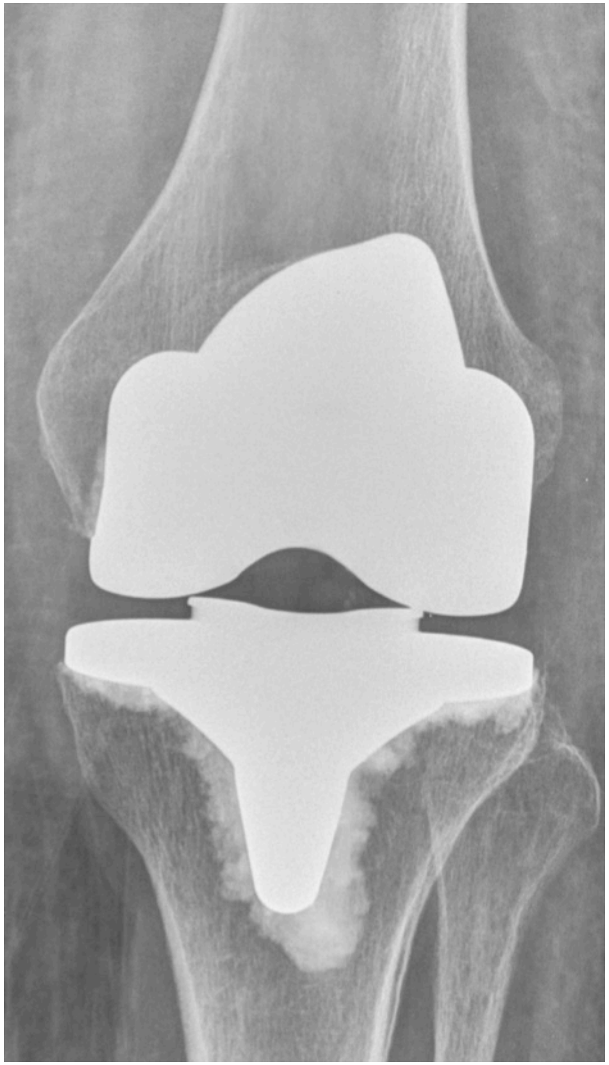

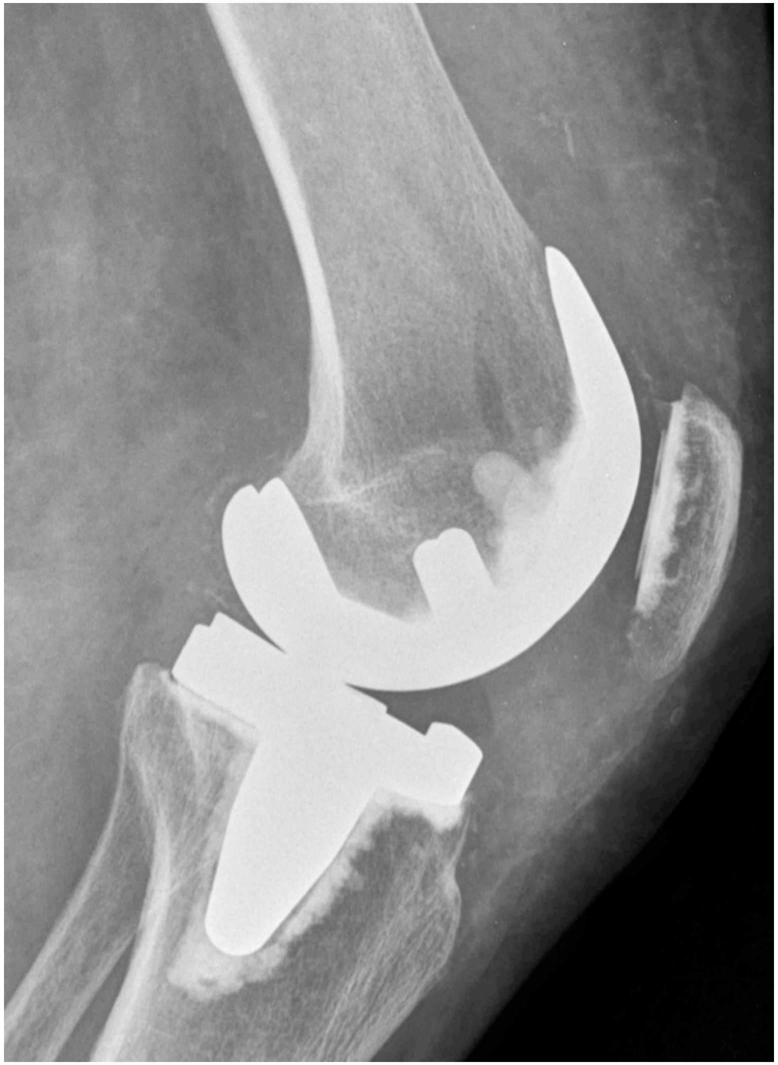

6 months postop

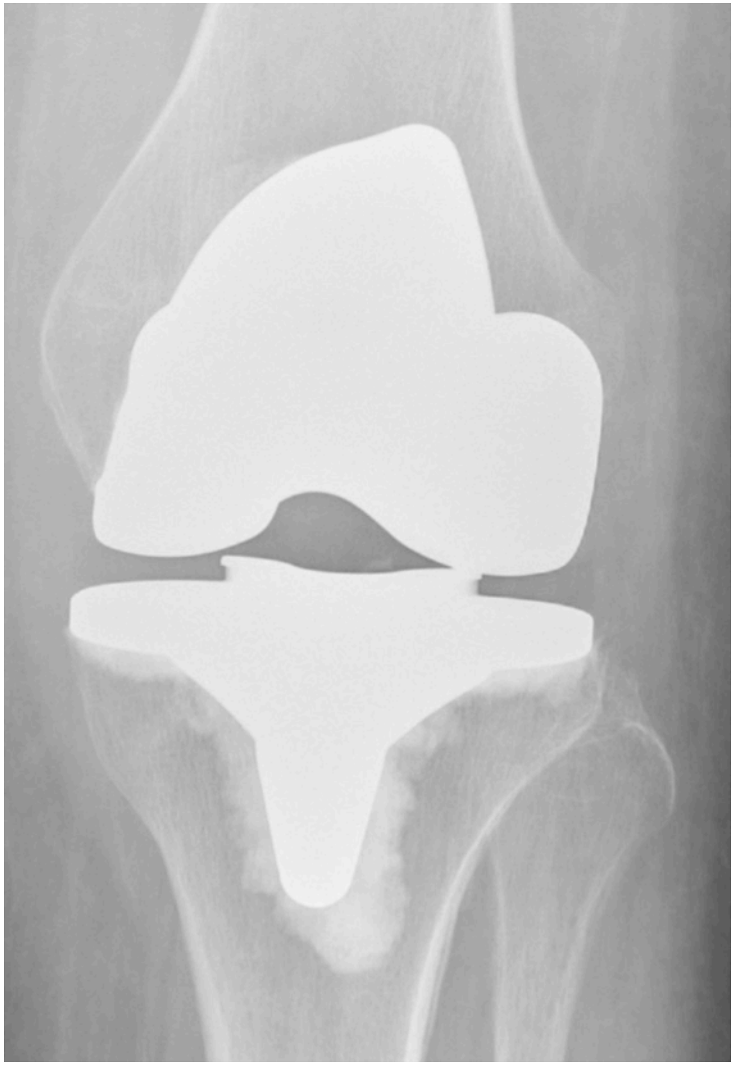

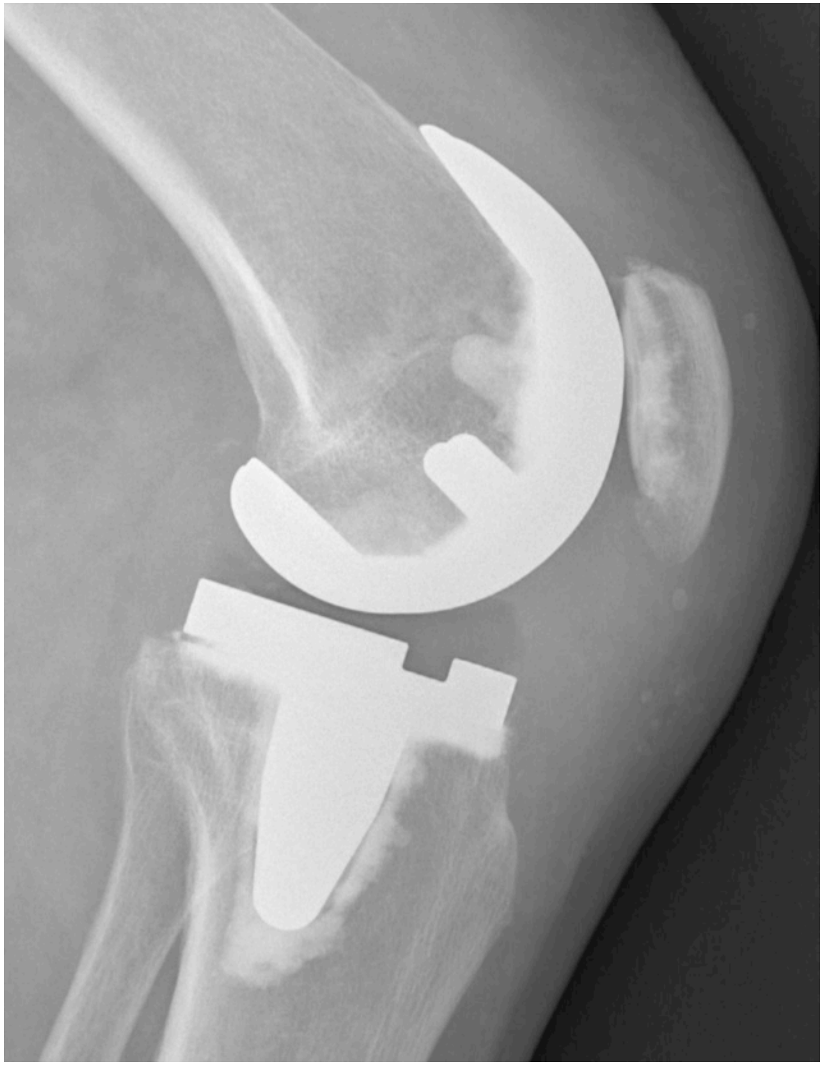

12 months postop

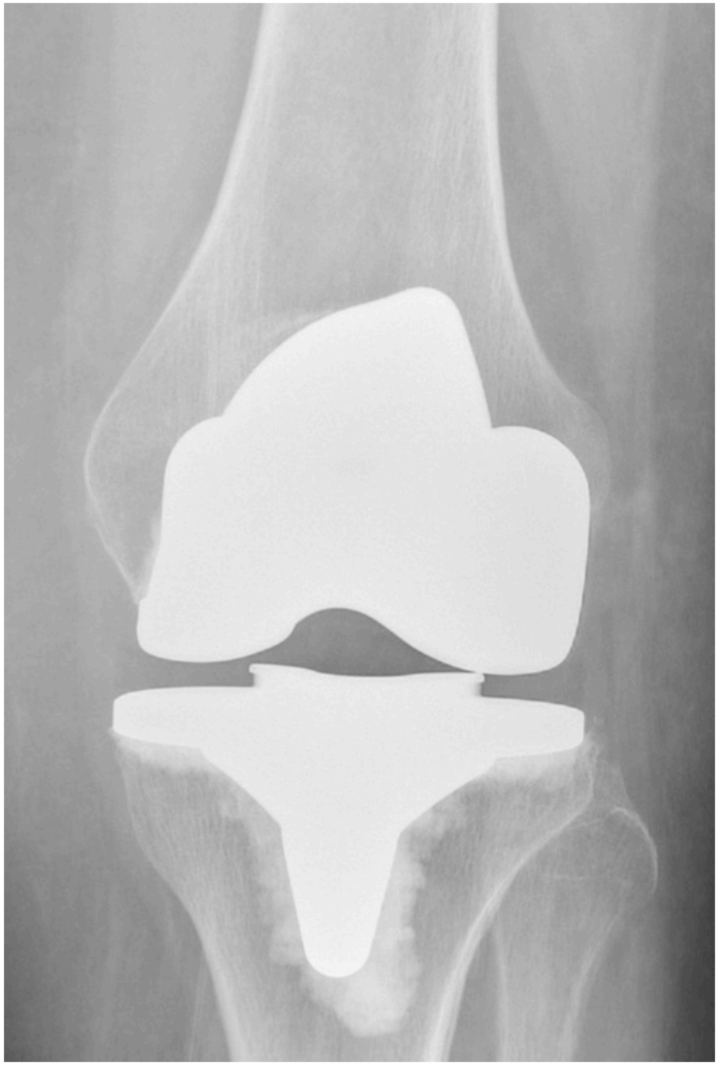

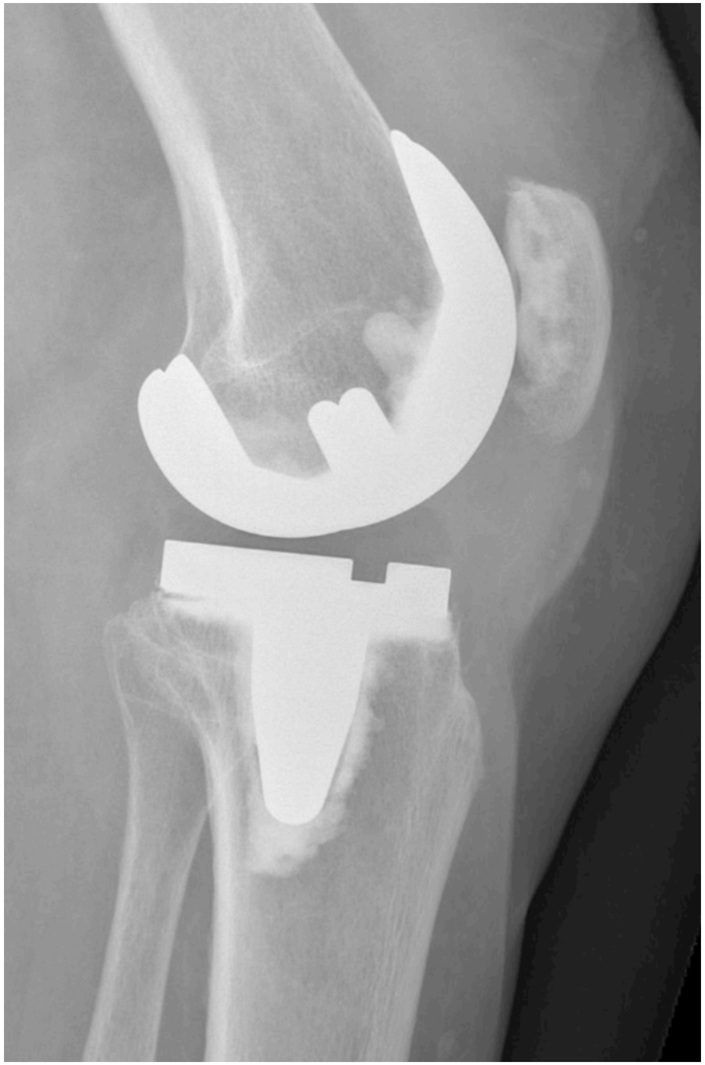

Case 9

preoperative

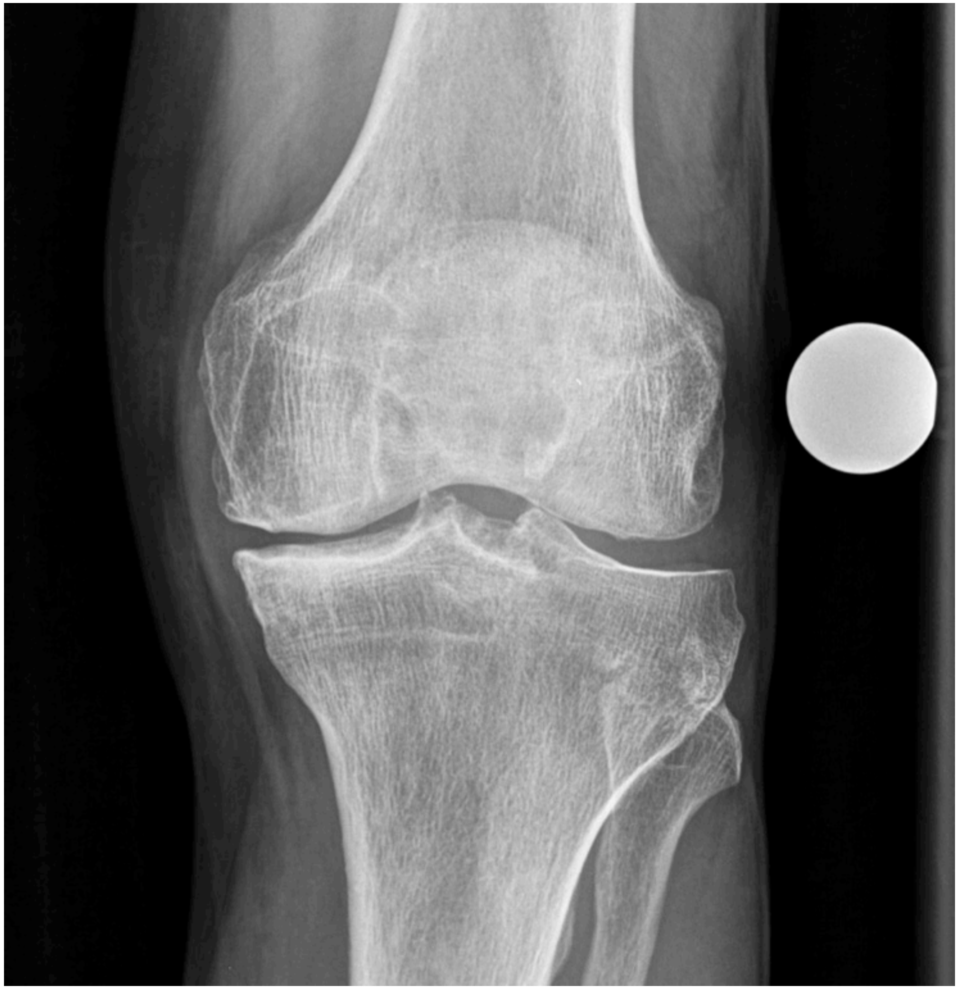

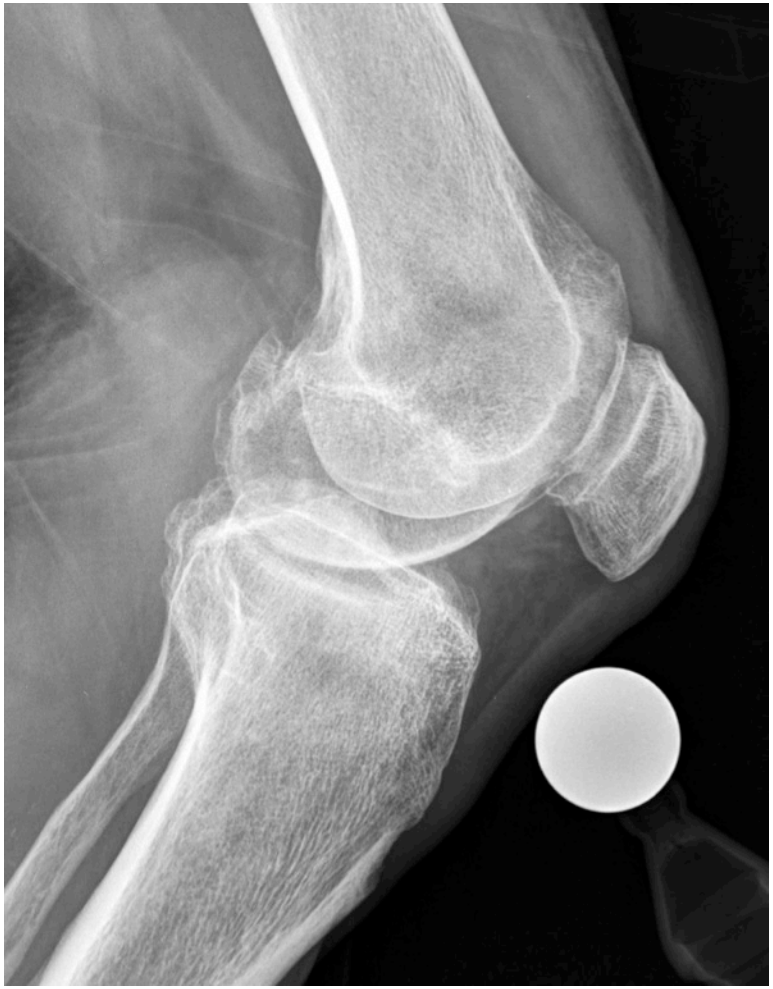

6 weeks postop

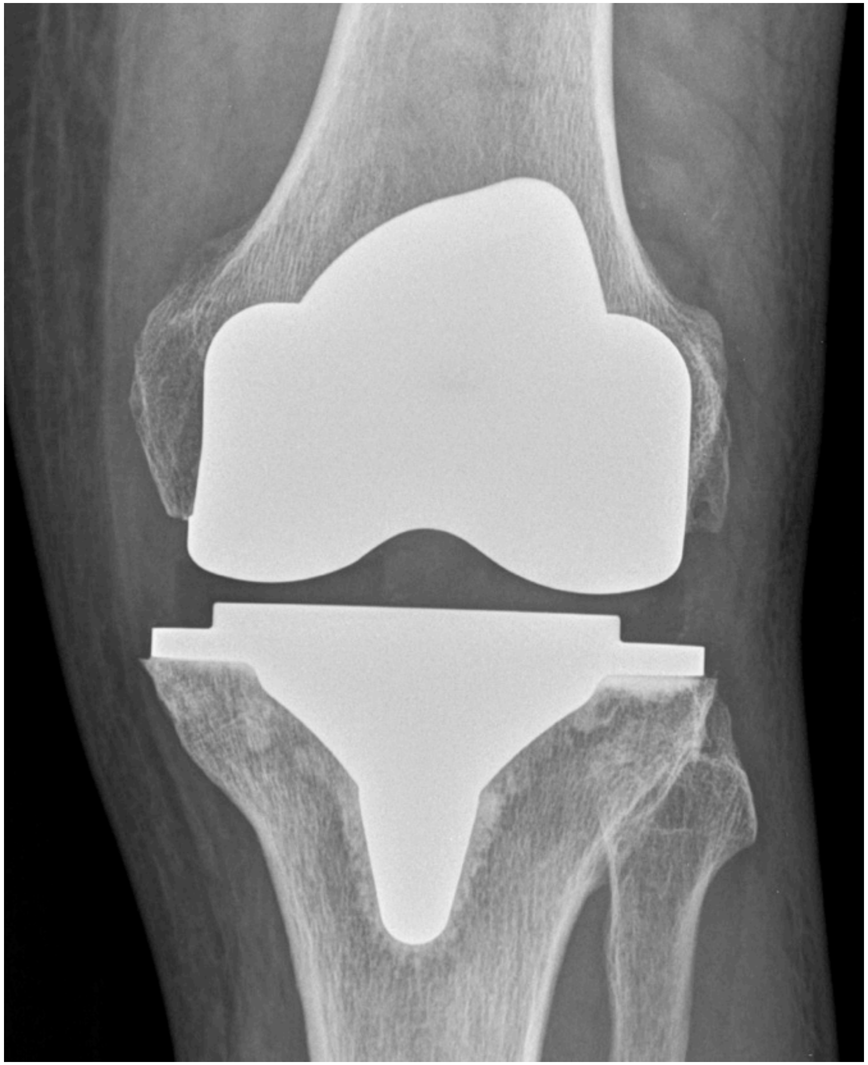

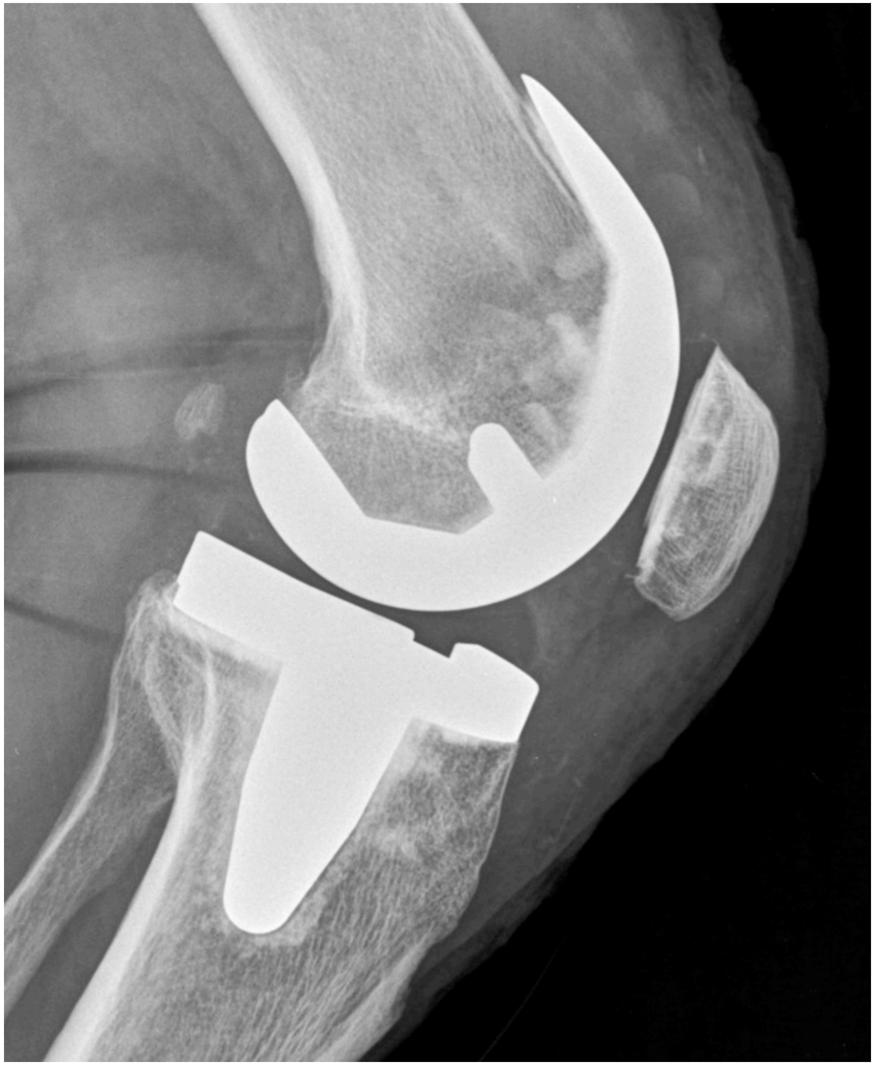

6 months postop

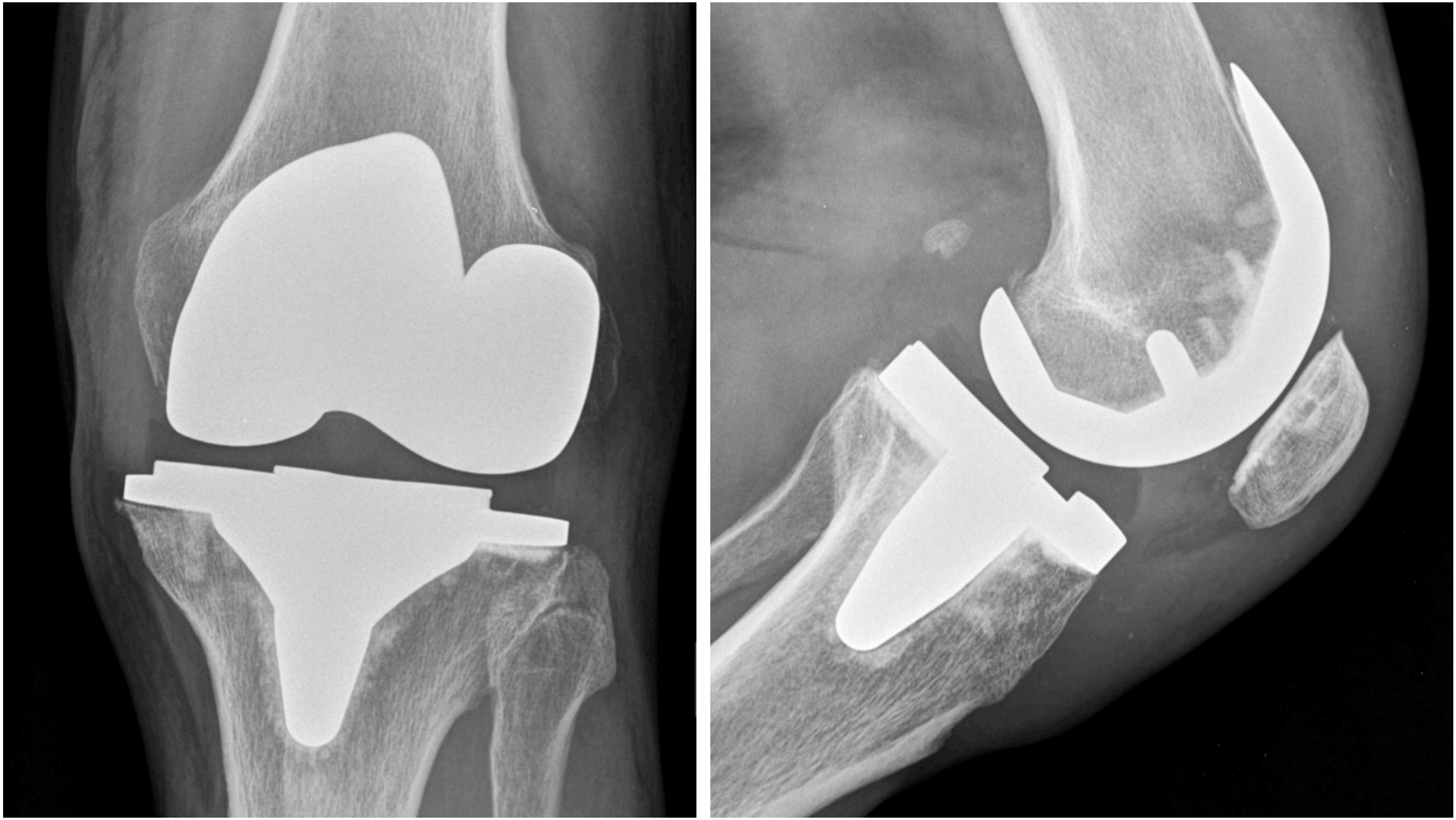

12 months postop

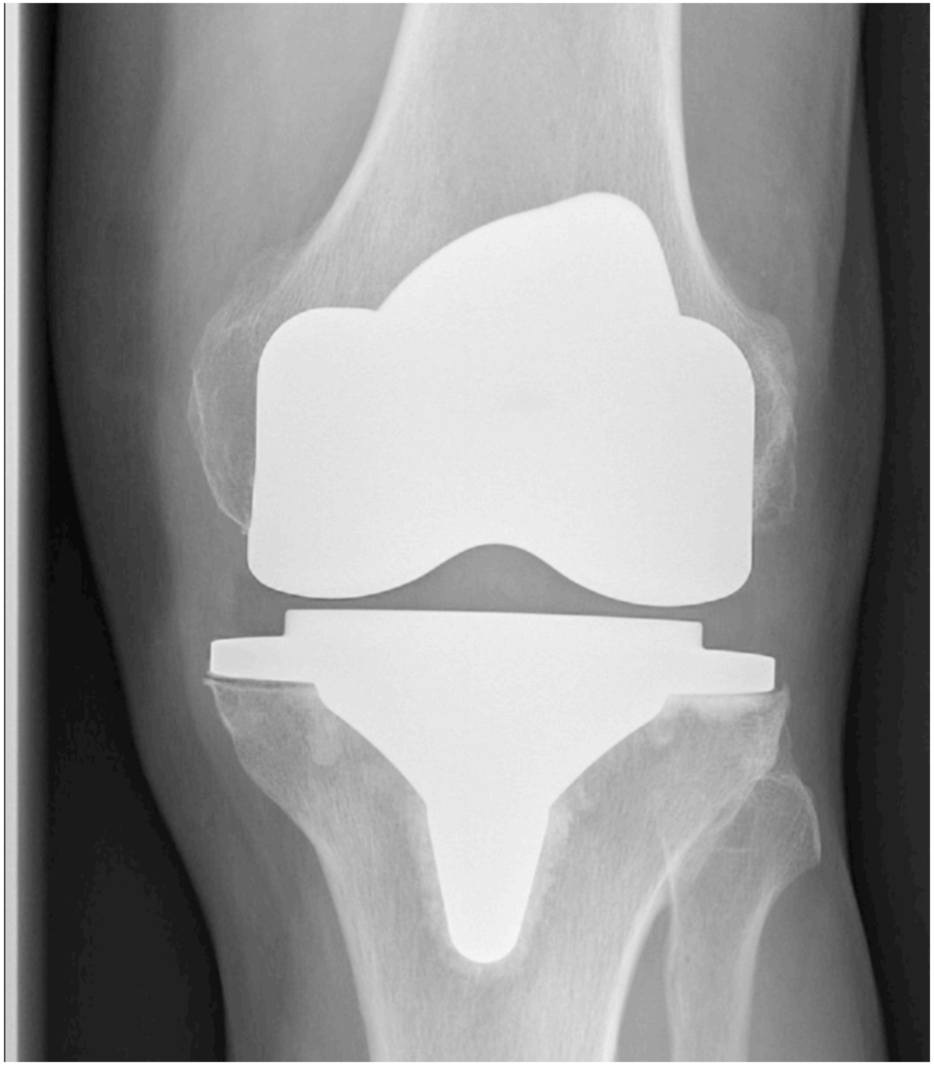

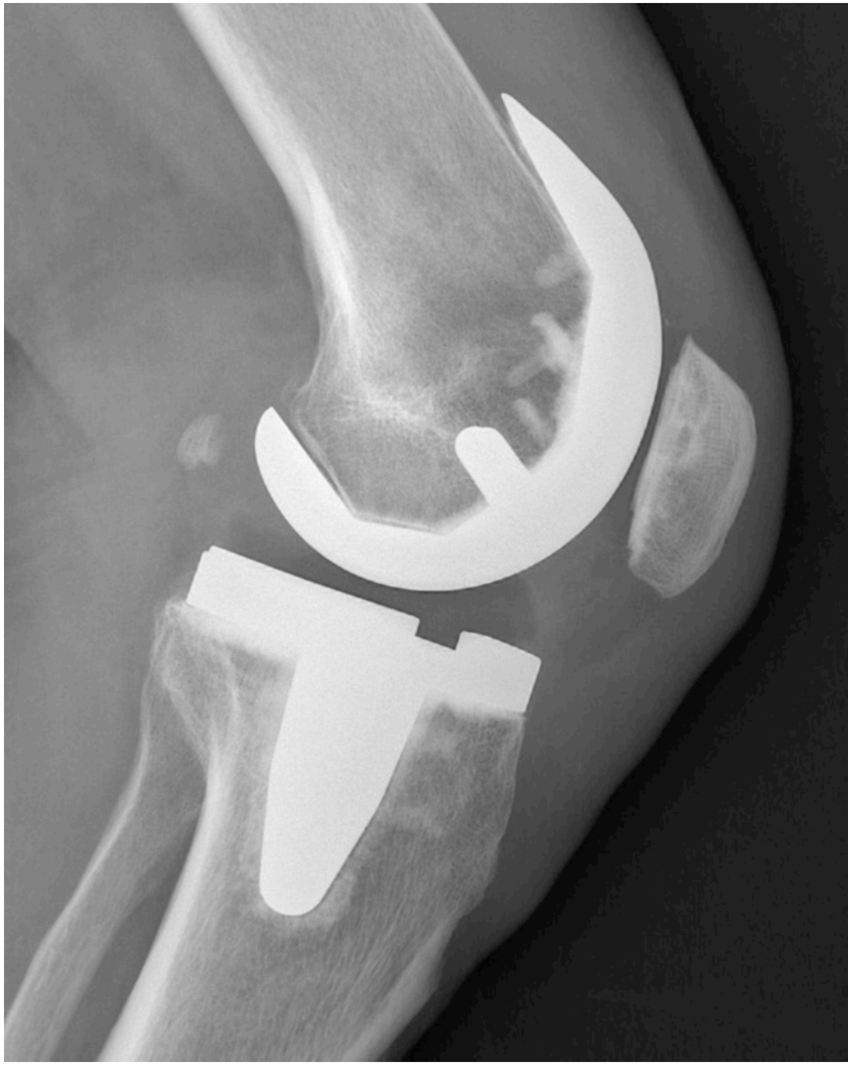

Case 10

preoperative

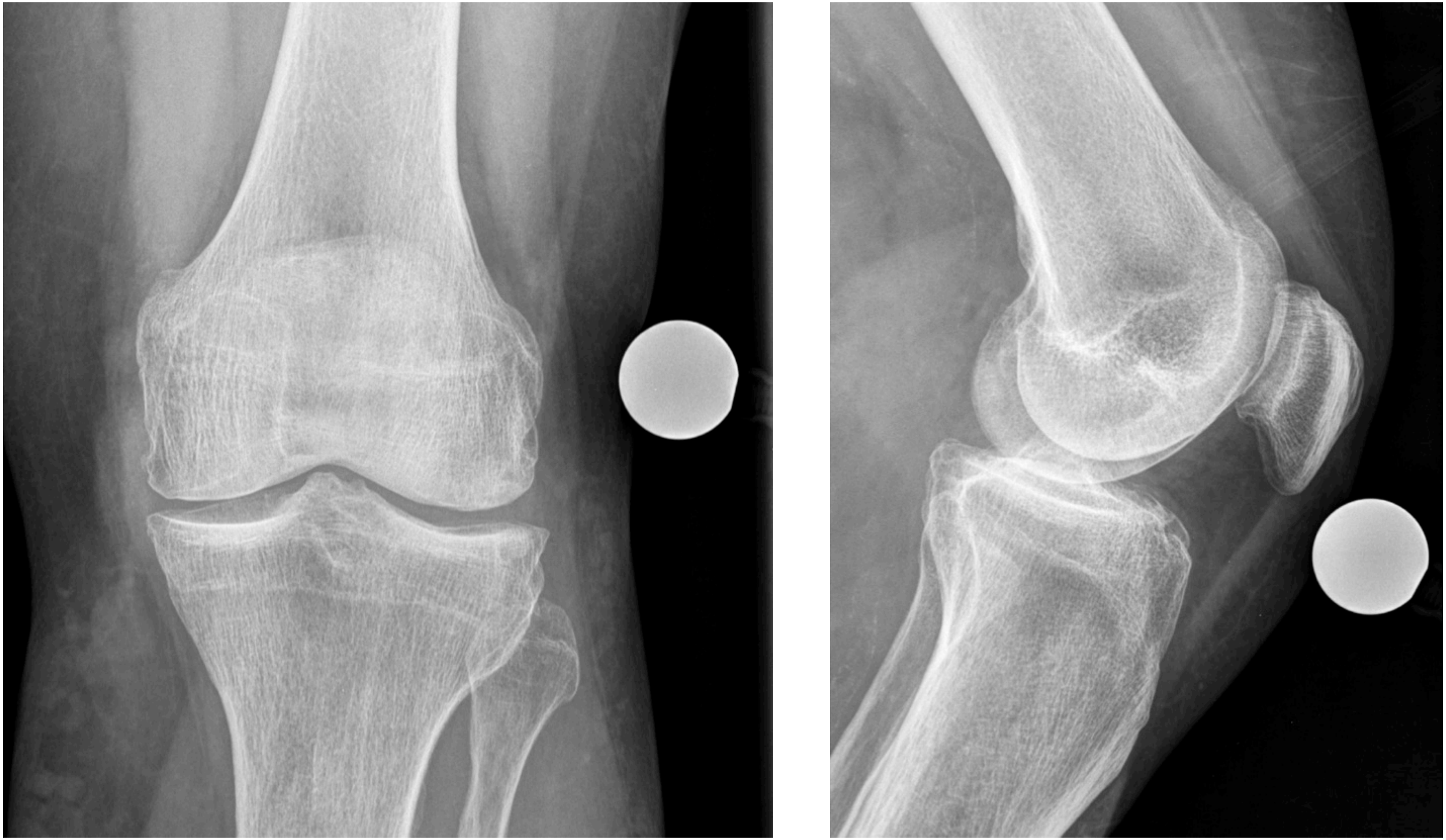

6 weeks postop

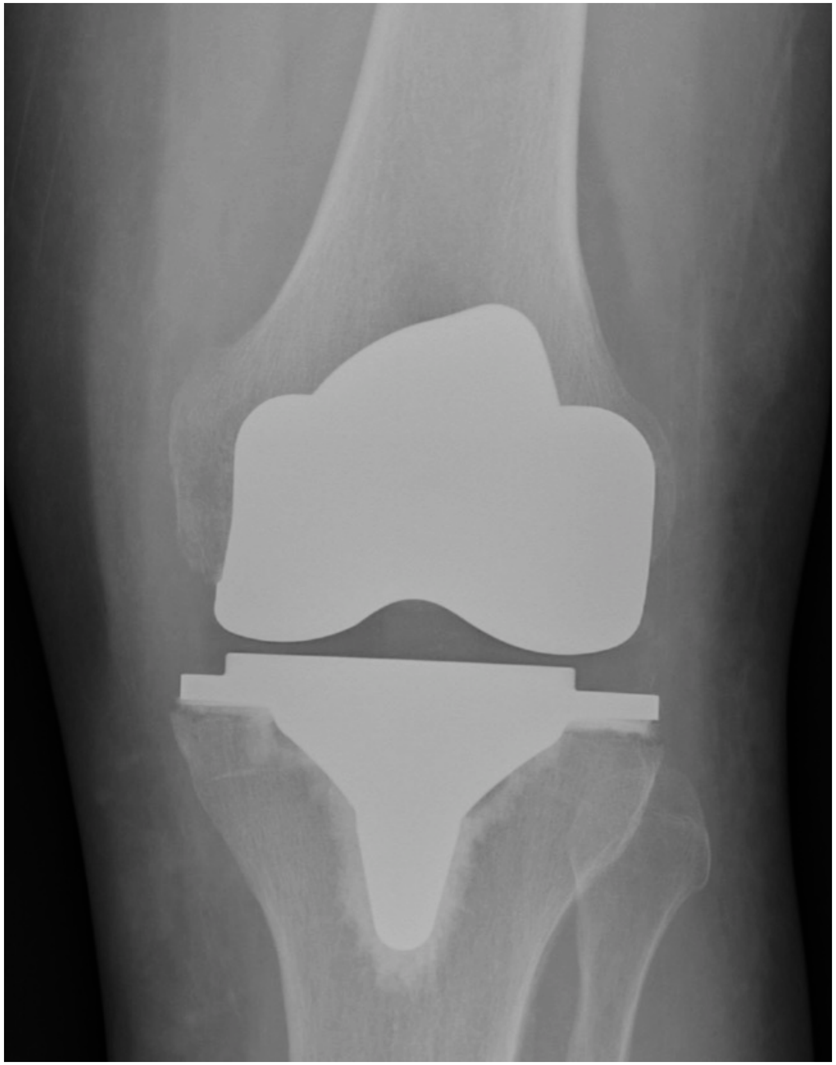

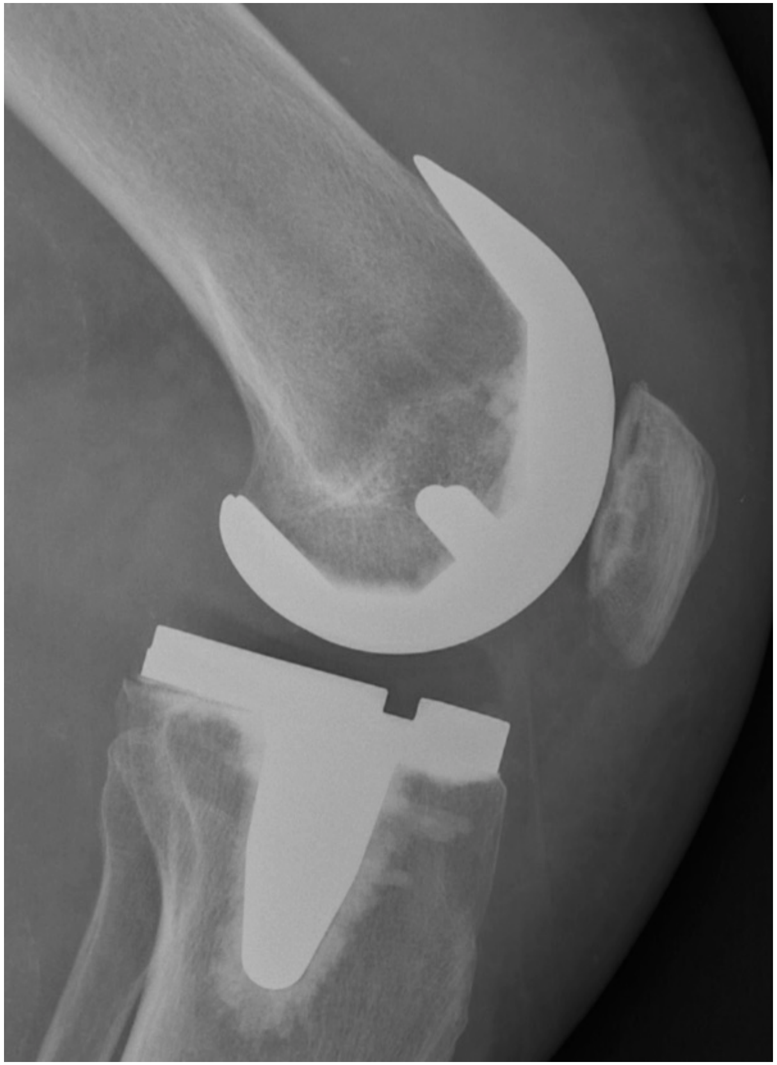

6 months postop

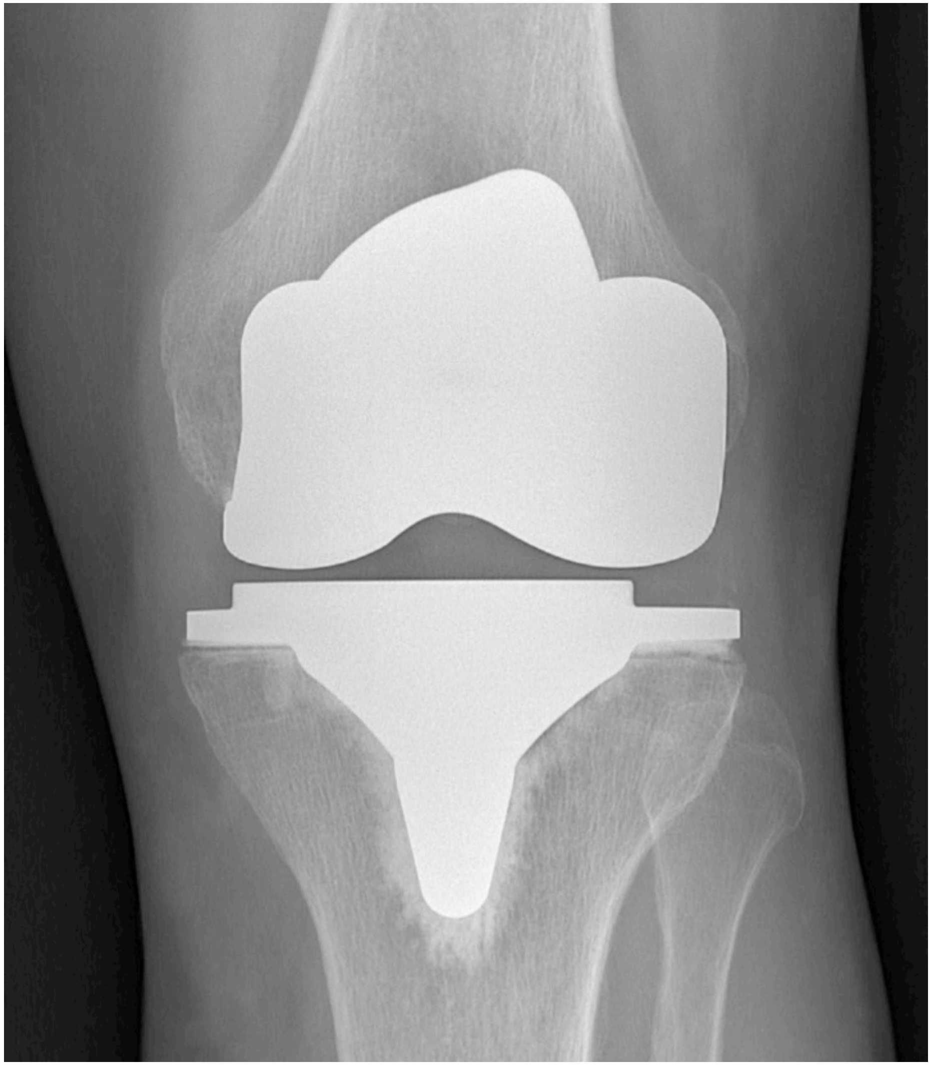

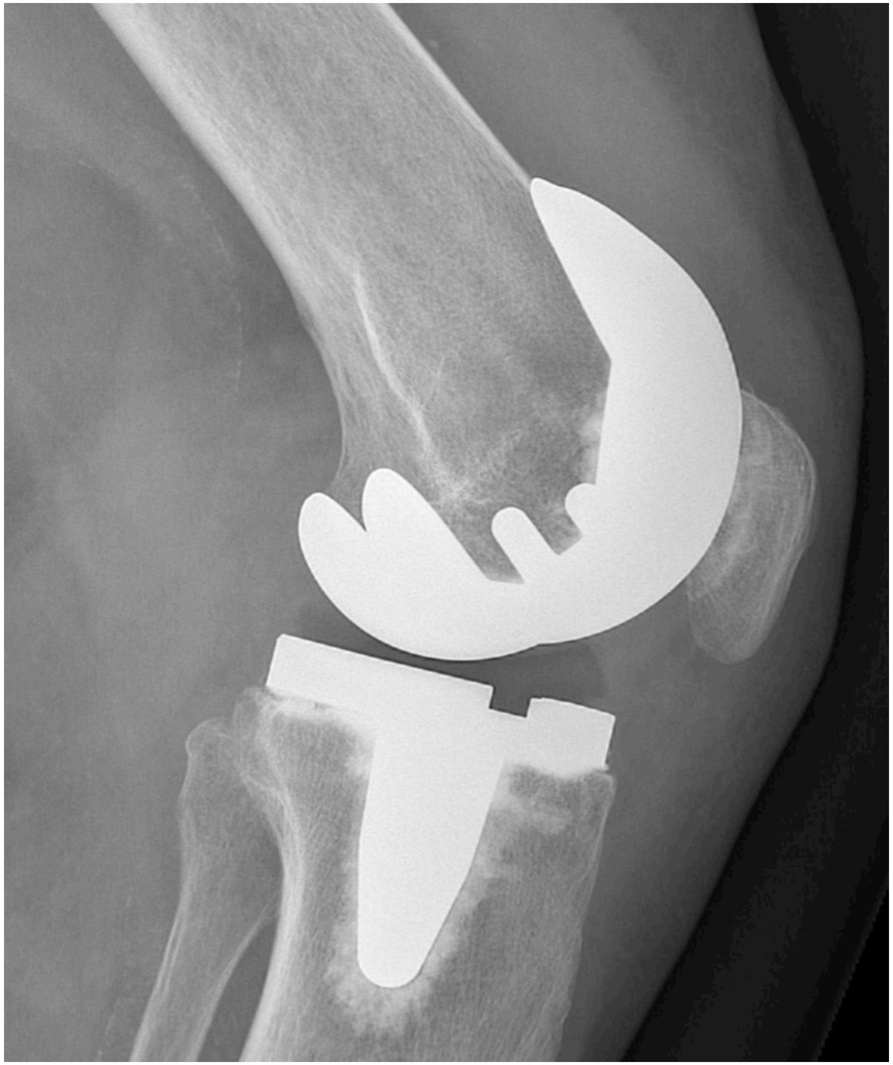

12 months postop

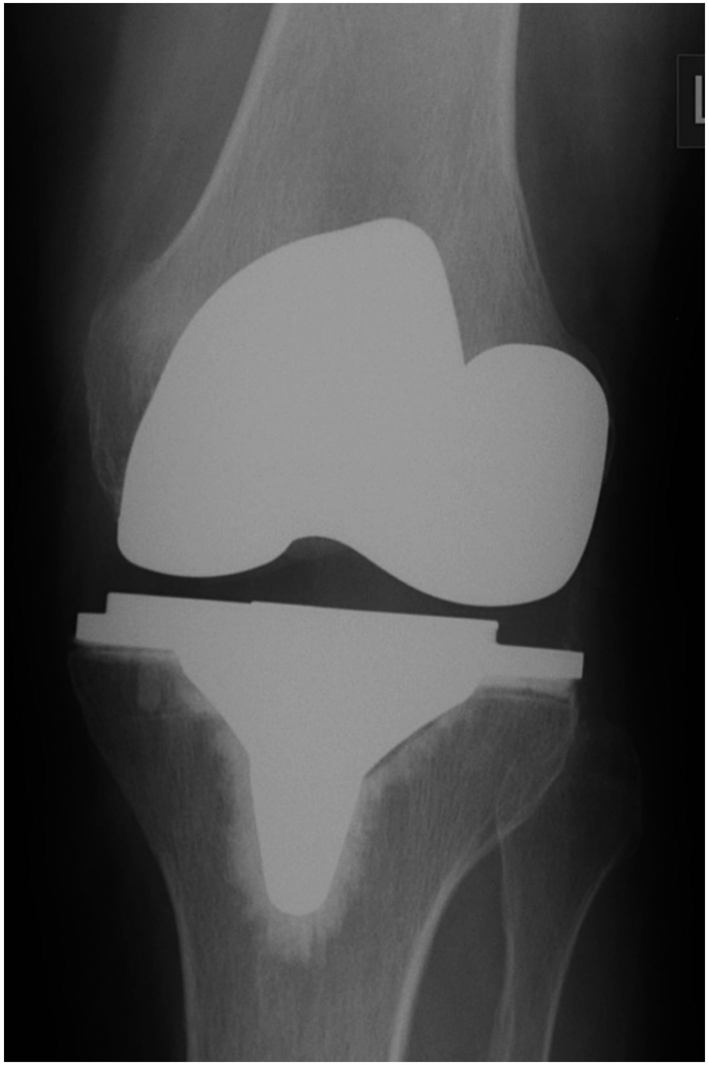

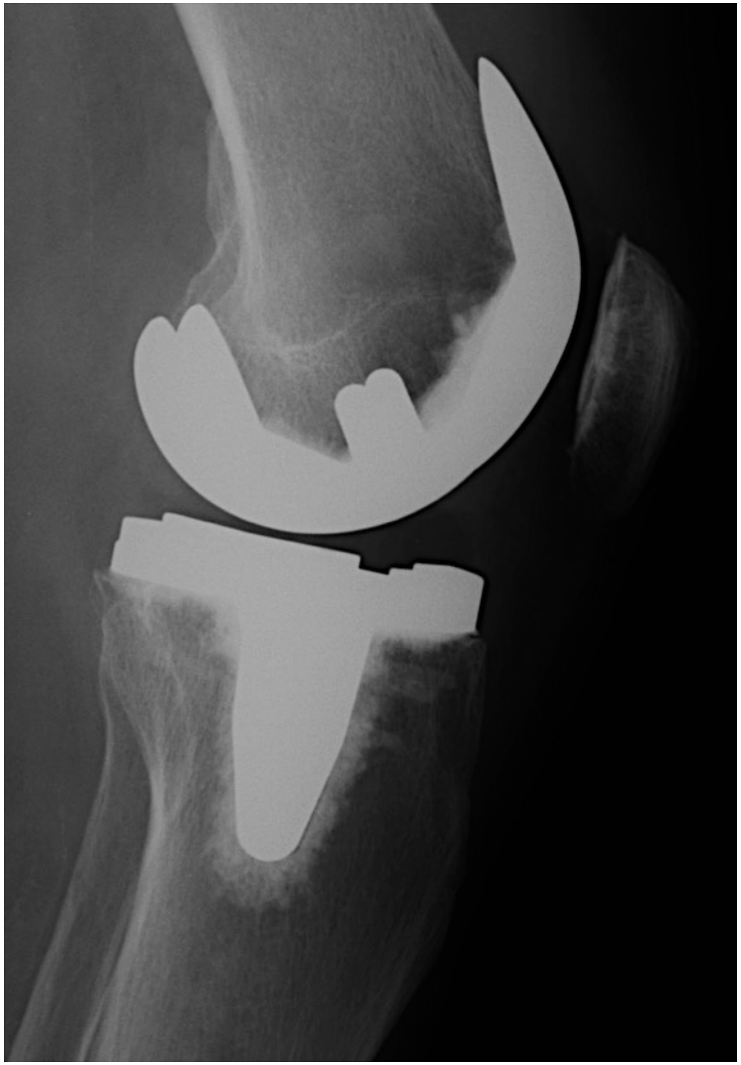

Case 11

preoperative

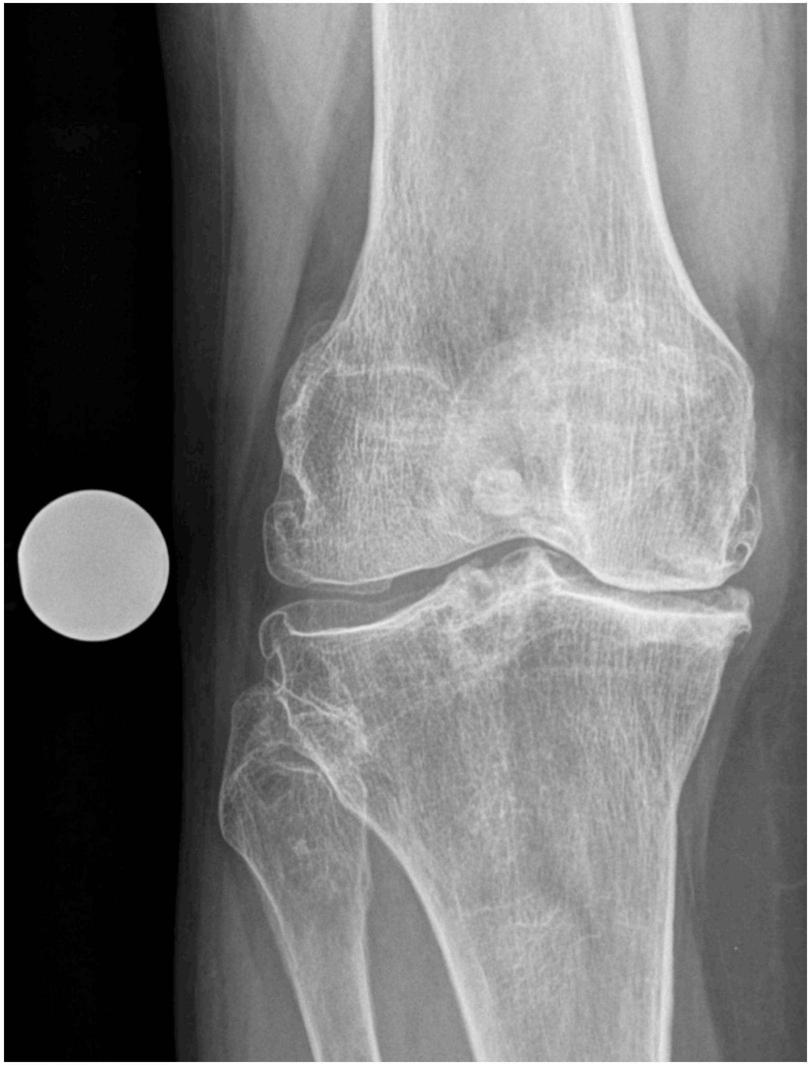

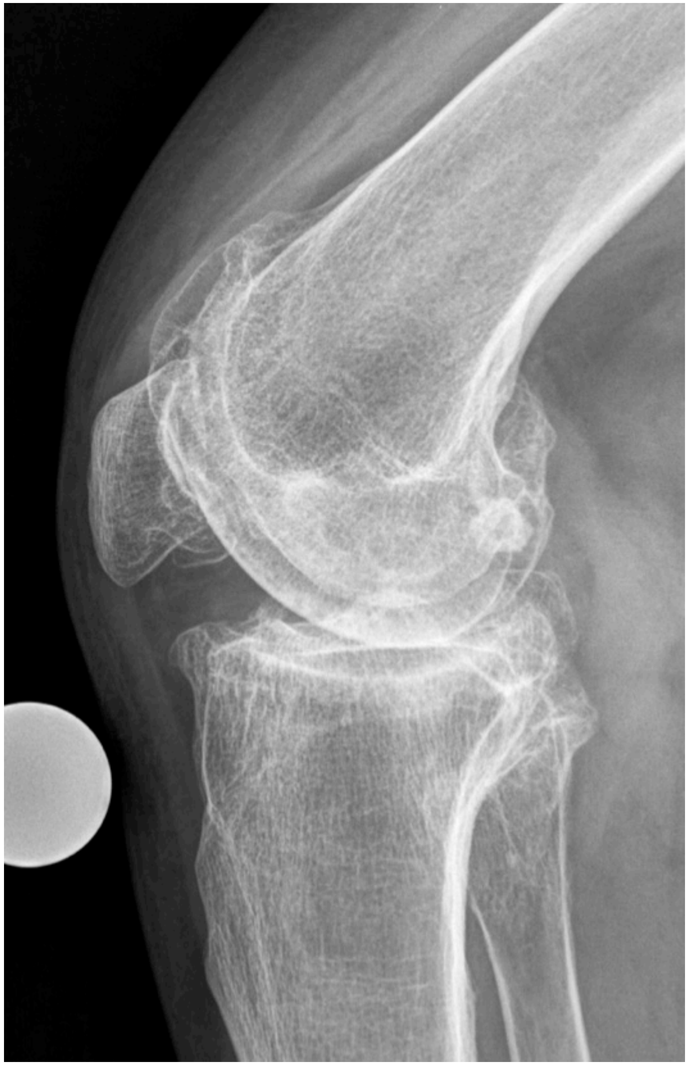

6 weeks postop

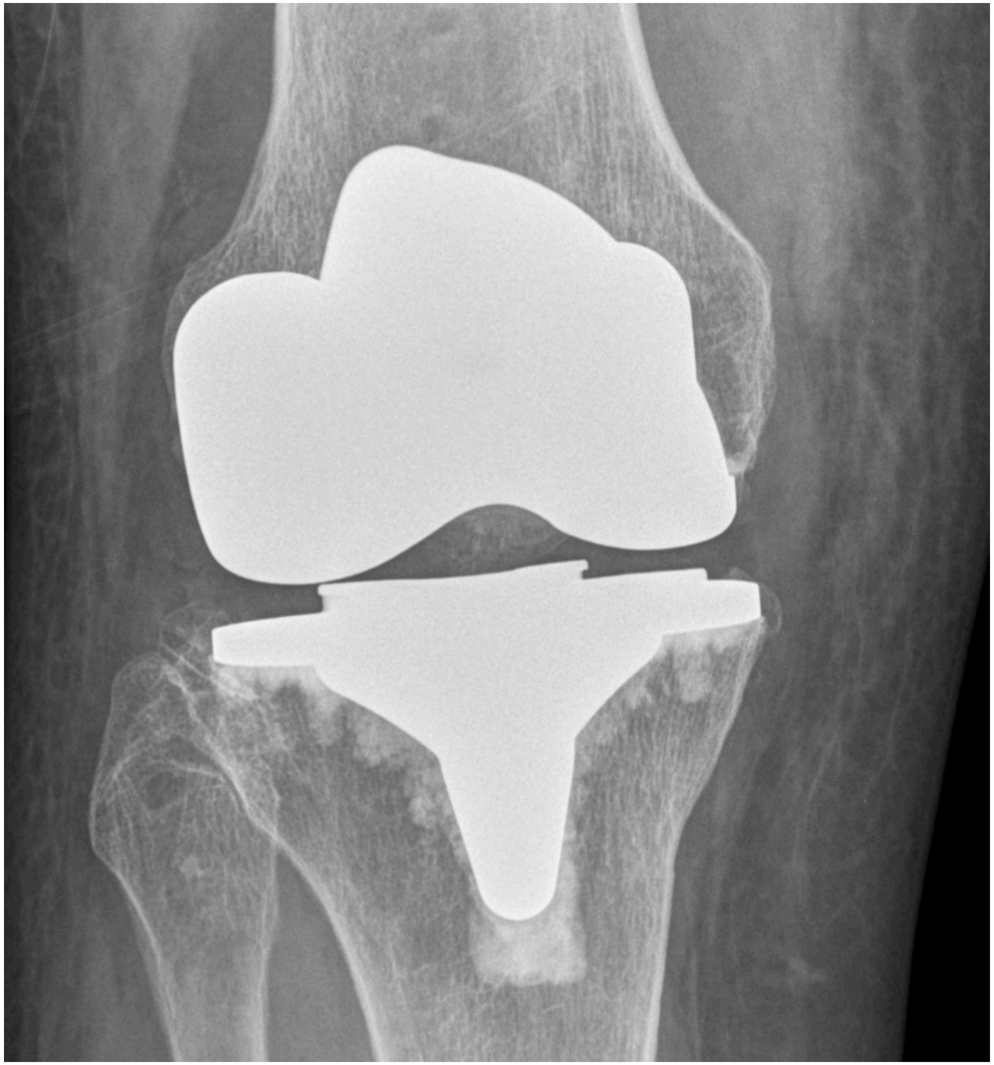

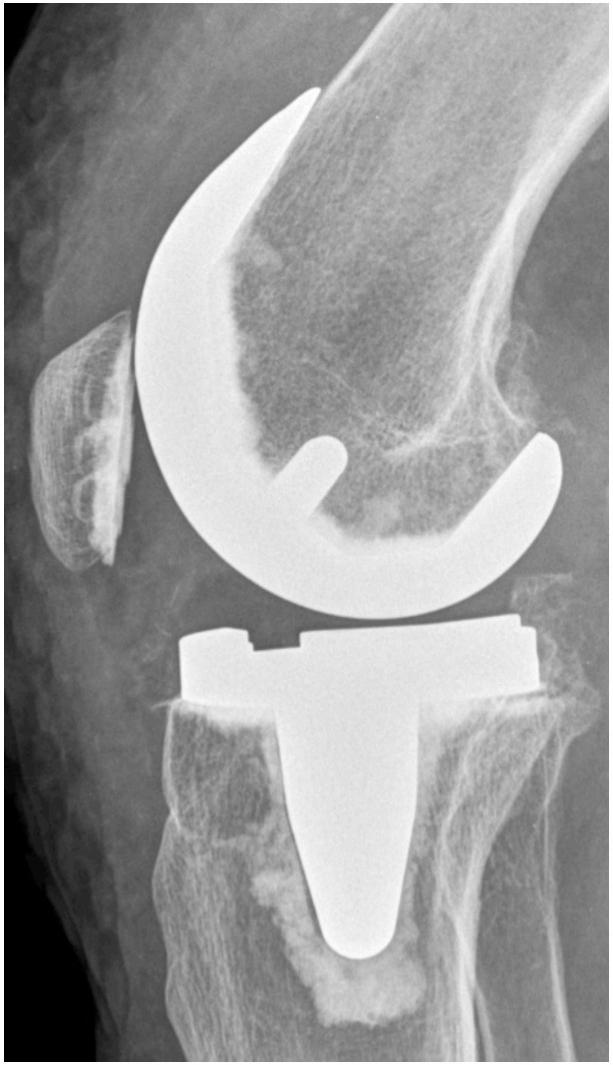

6 months postop

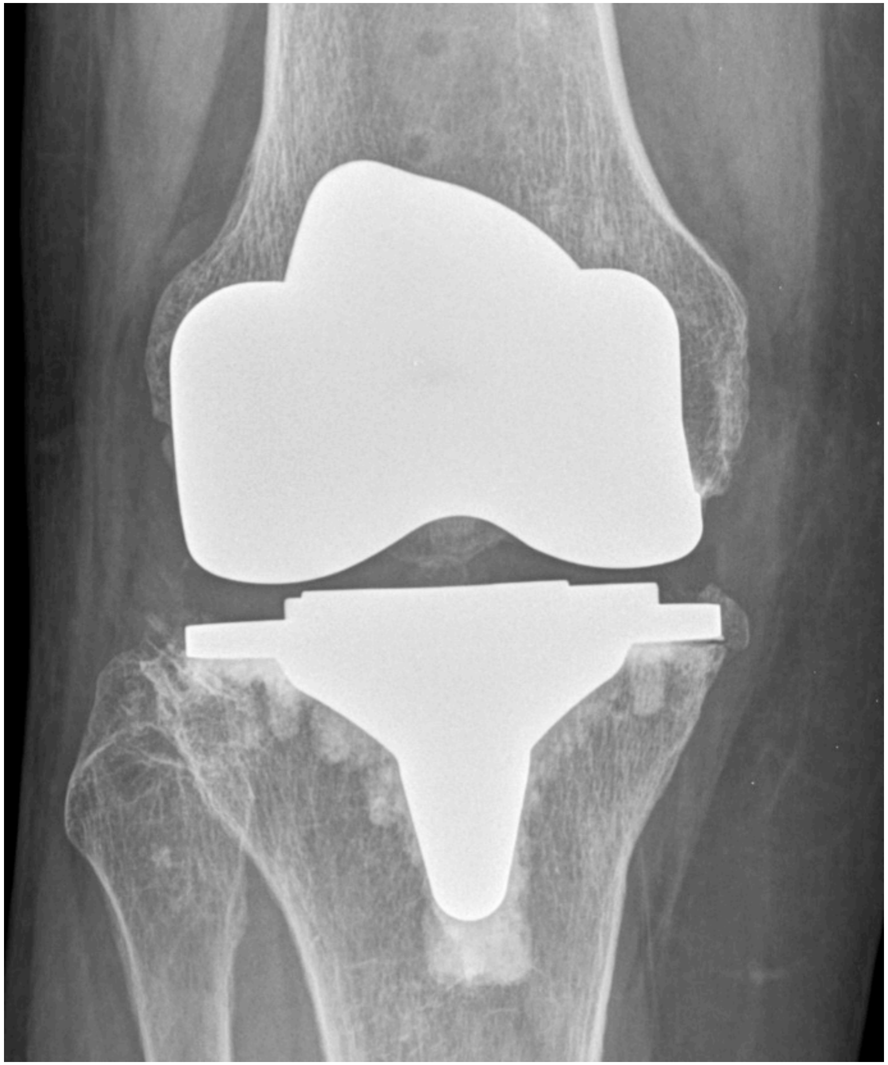

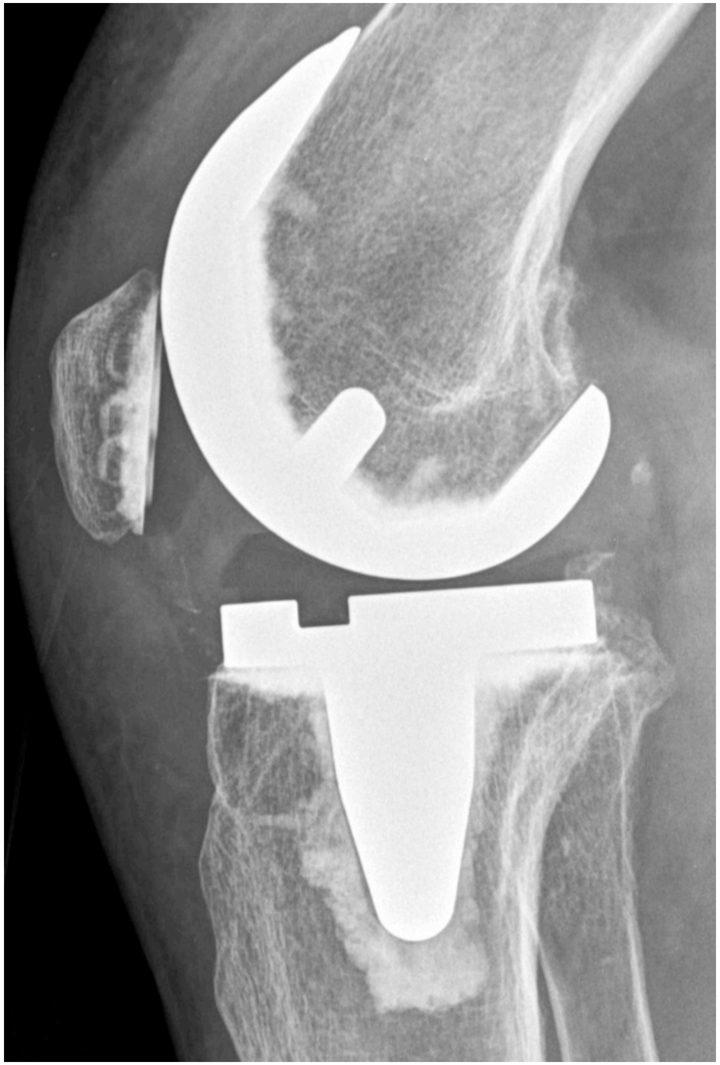

12 months postop

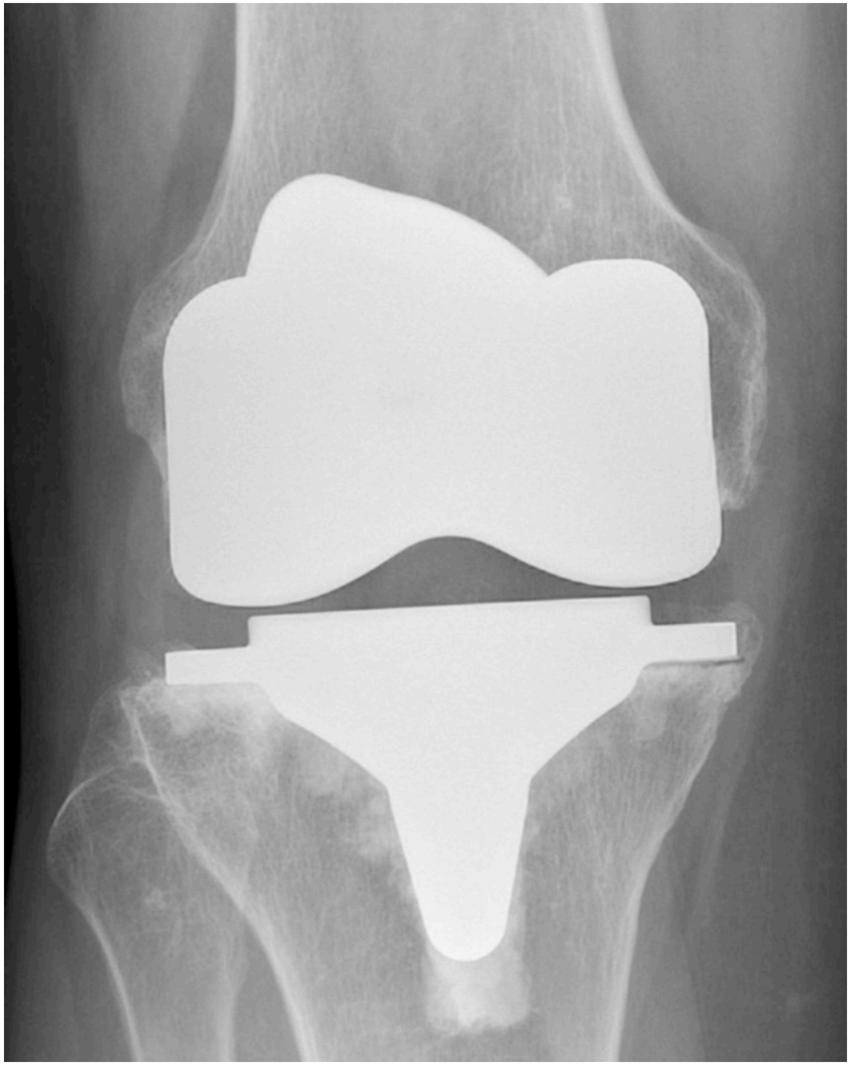

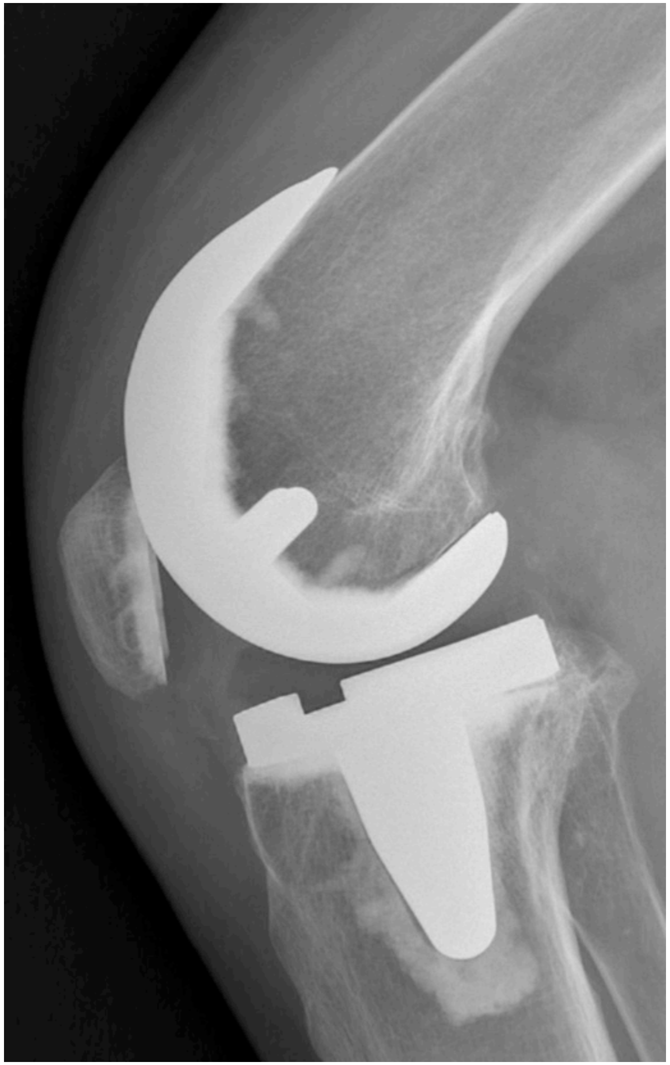

Case 12

preoperative

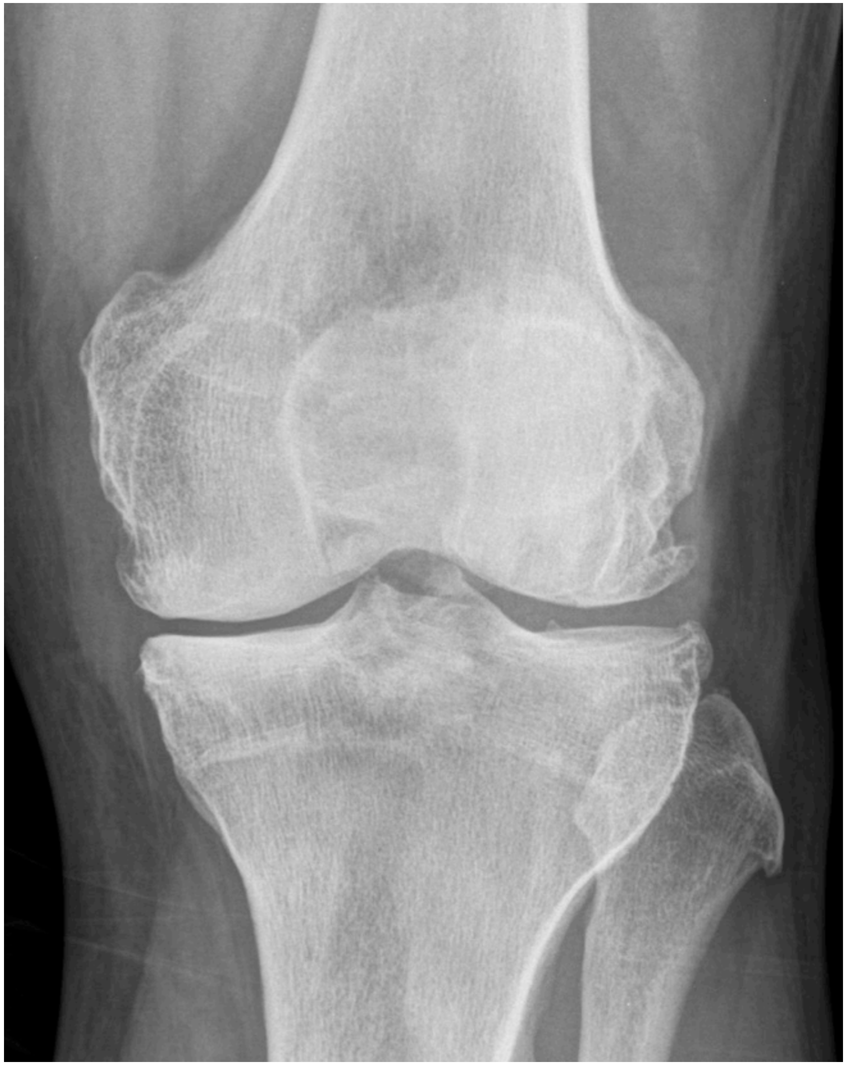

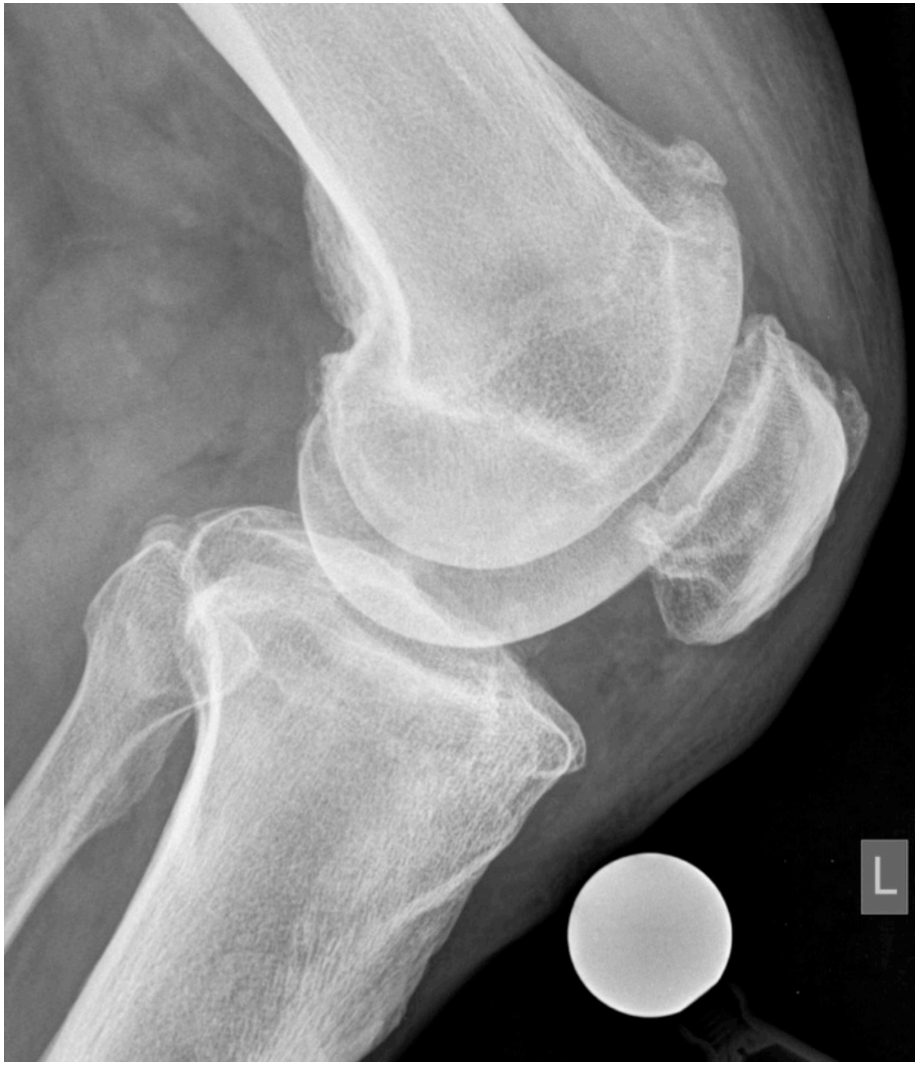

6 weeks postop

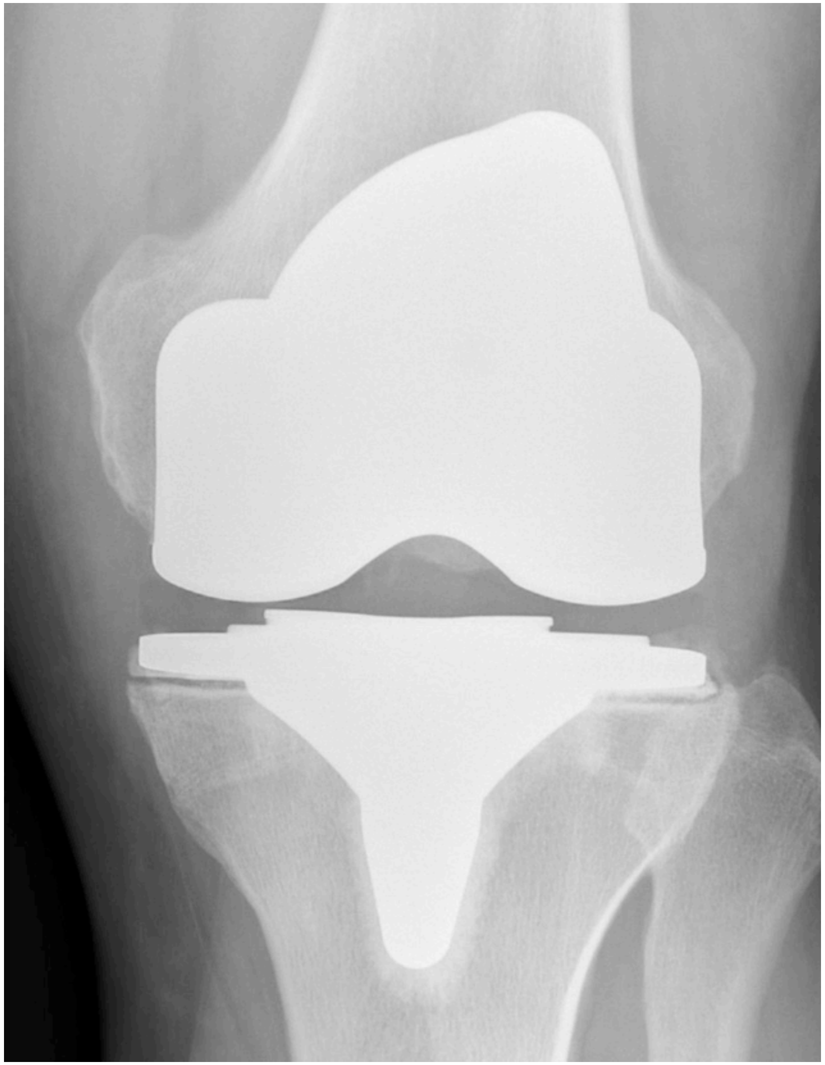

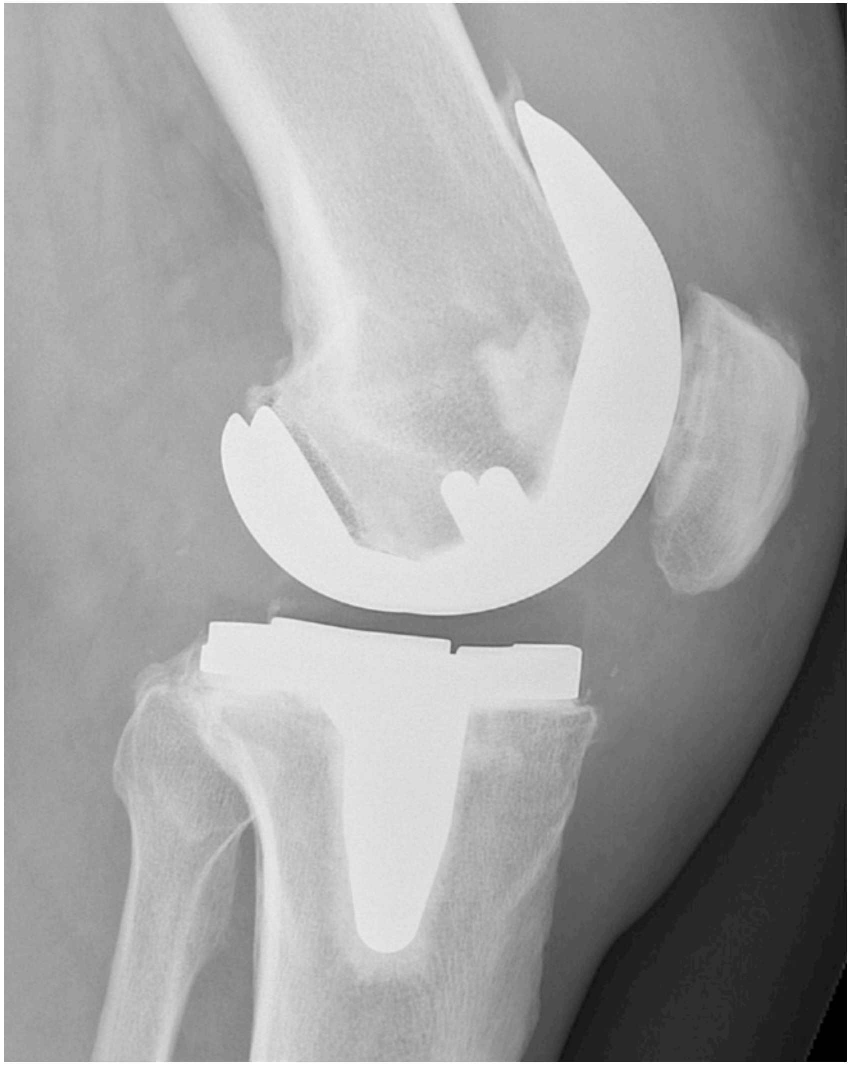

6 months postop

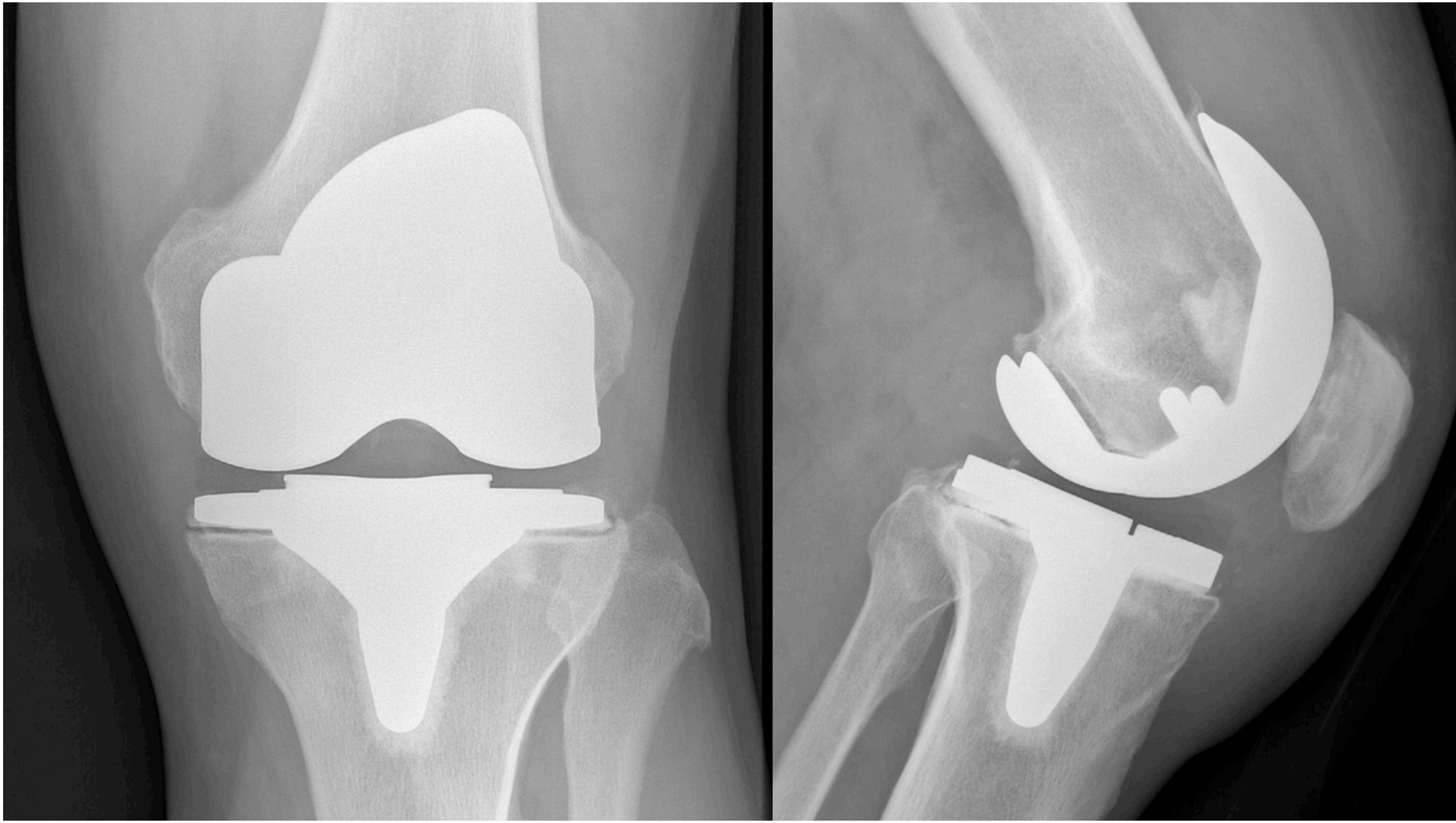

12 months postop

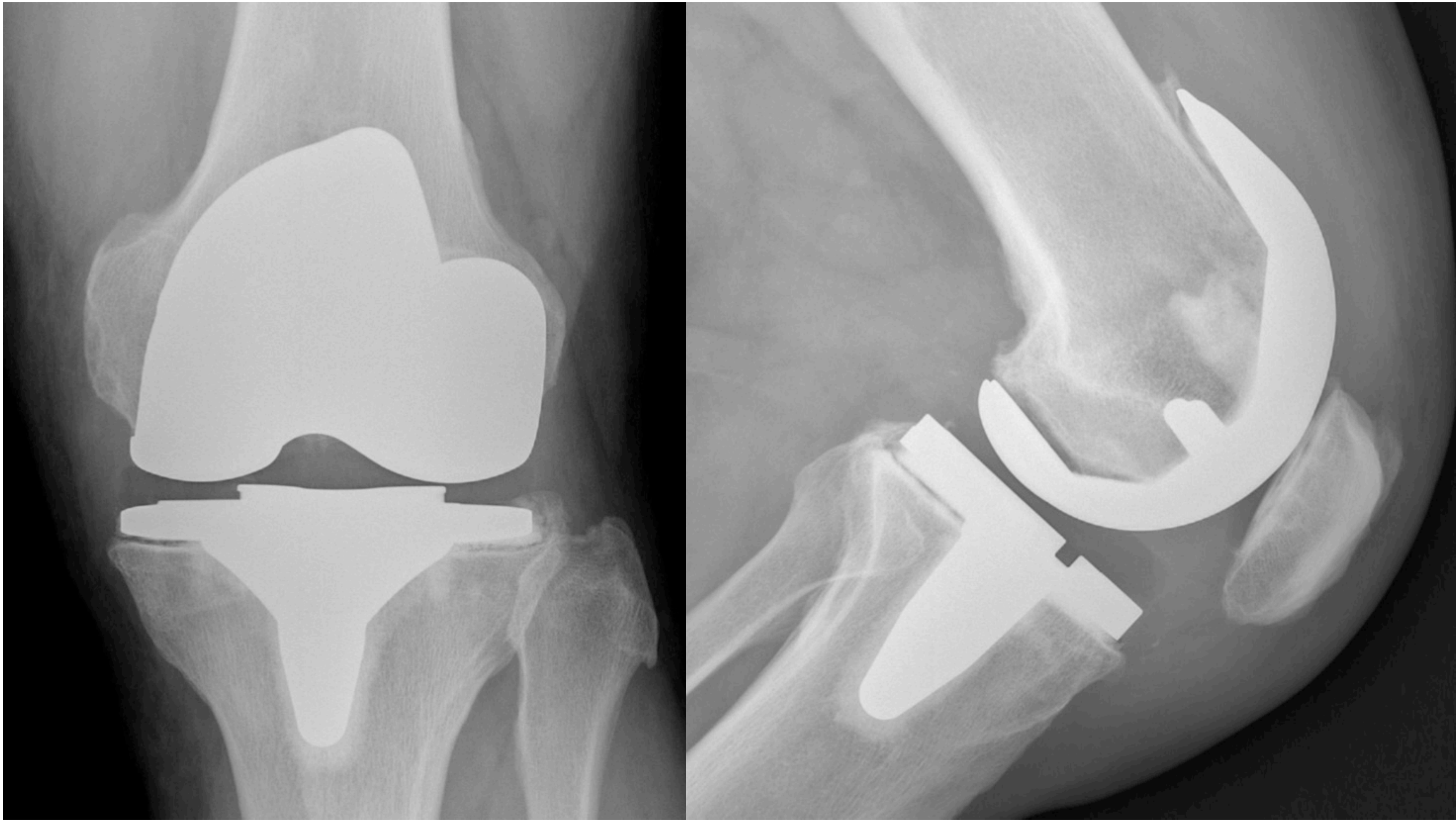

Case 13

preoperative

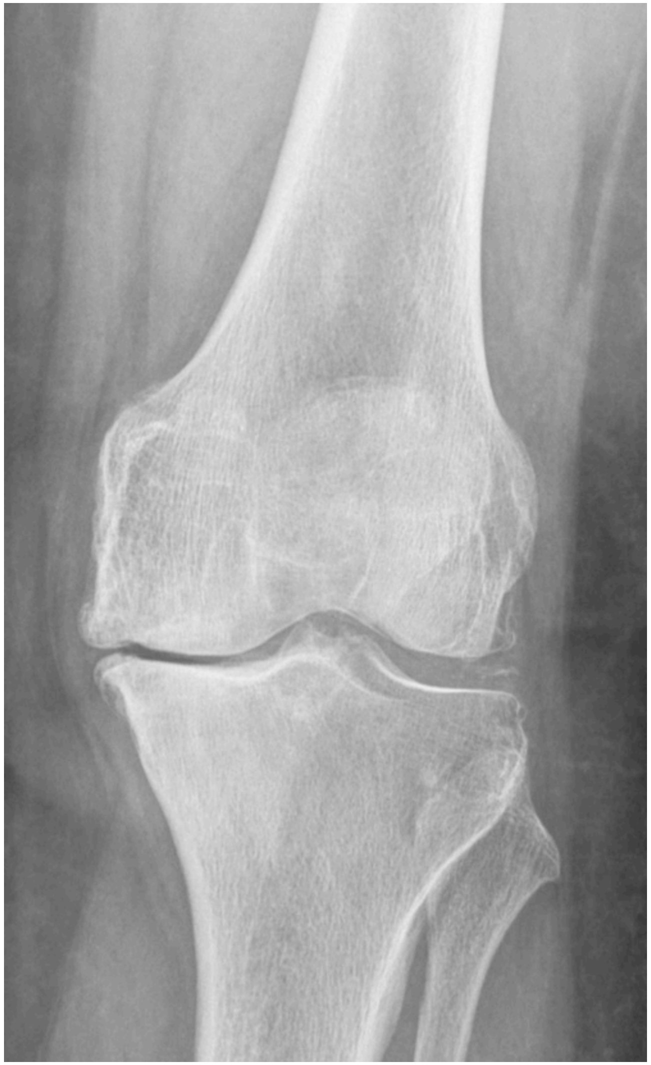

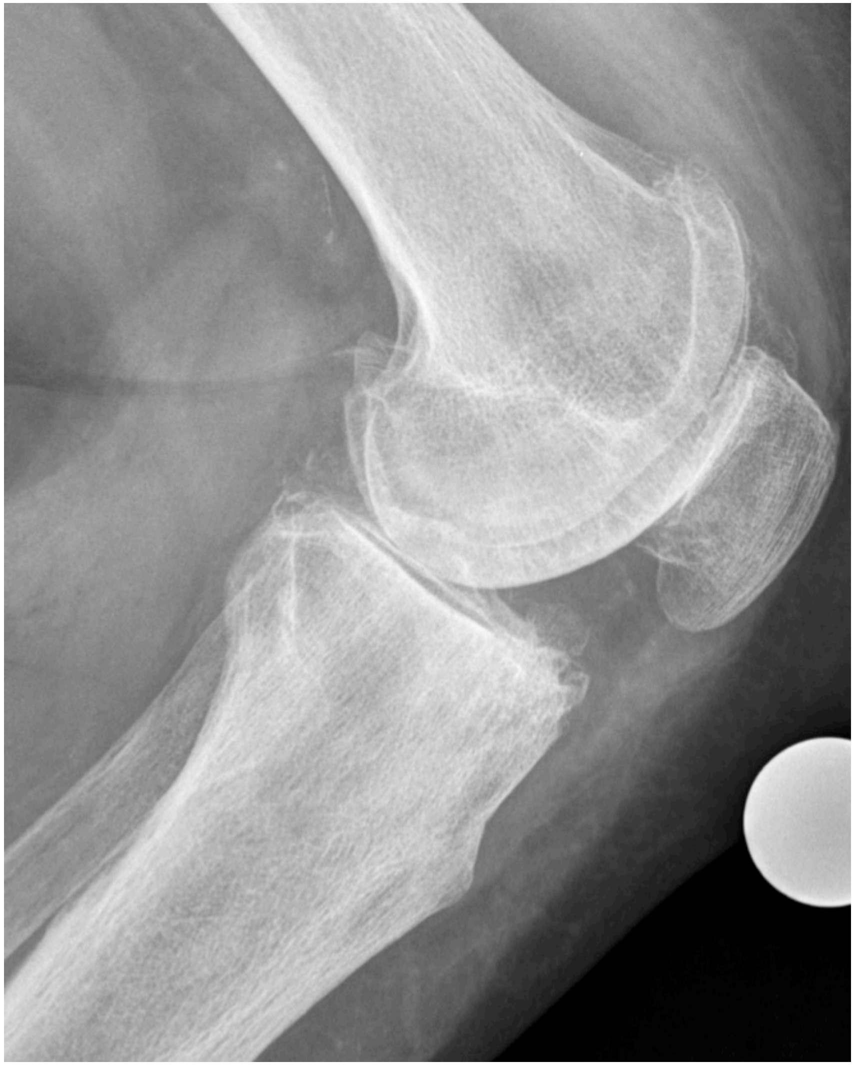

6 weeks postop

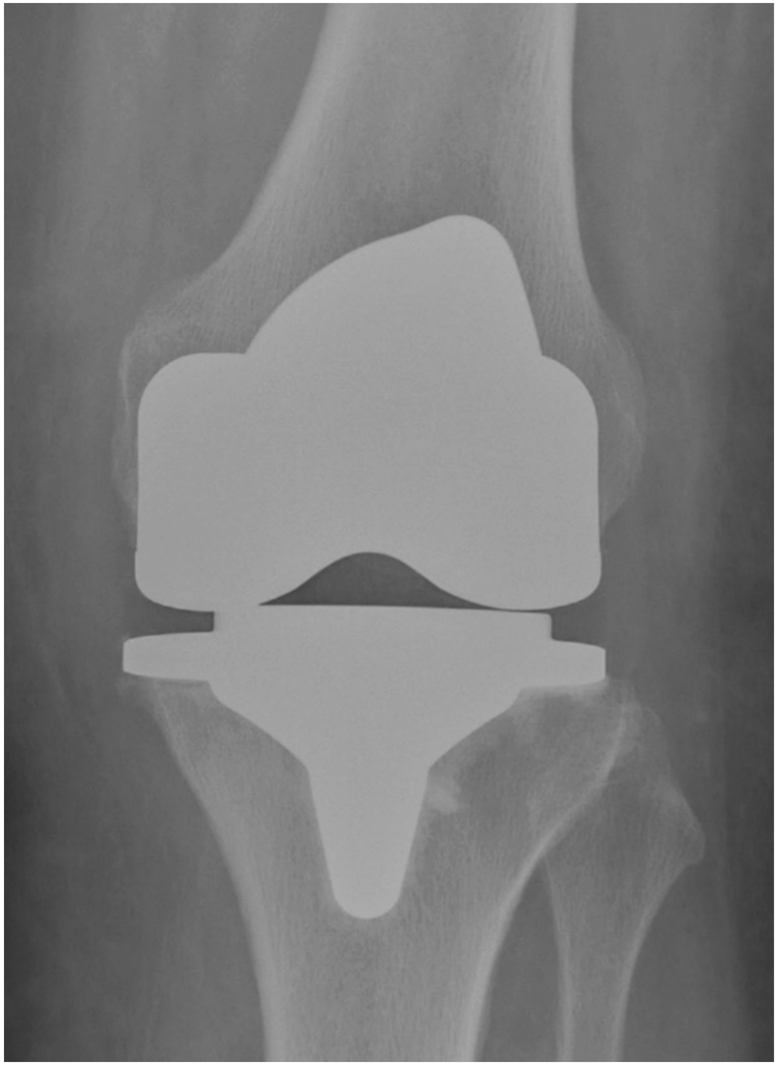

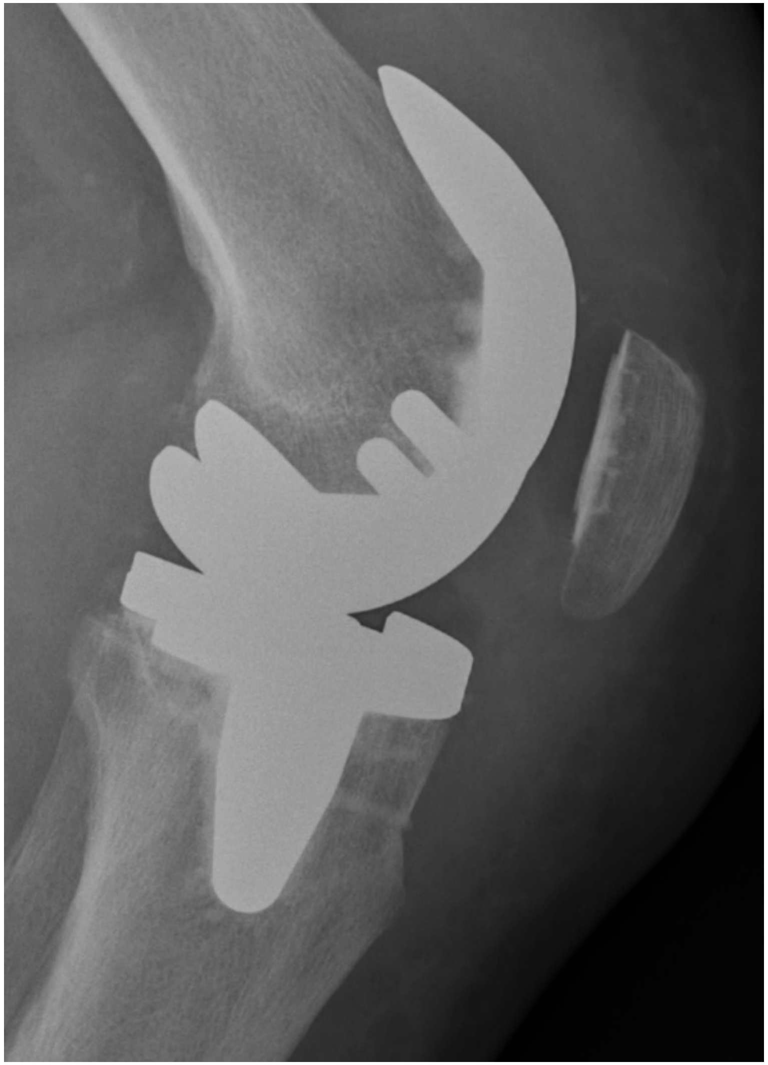

6 months postop

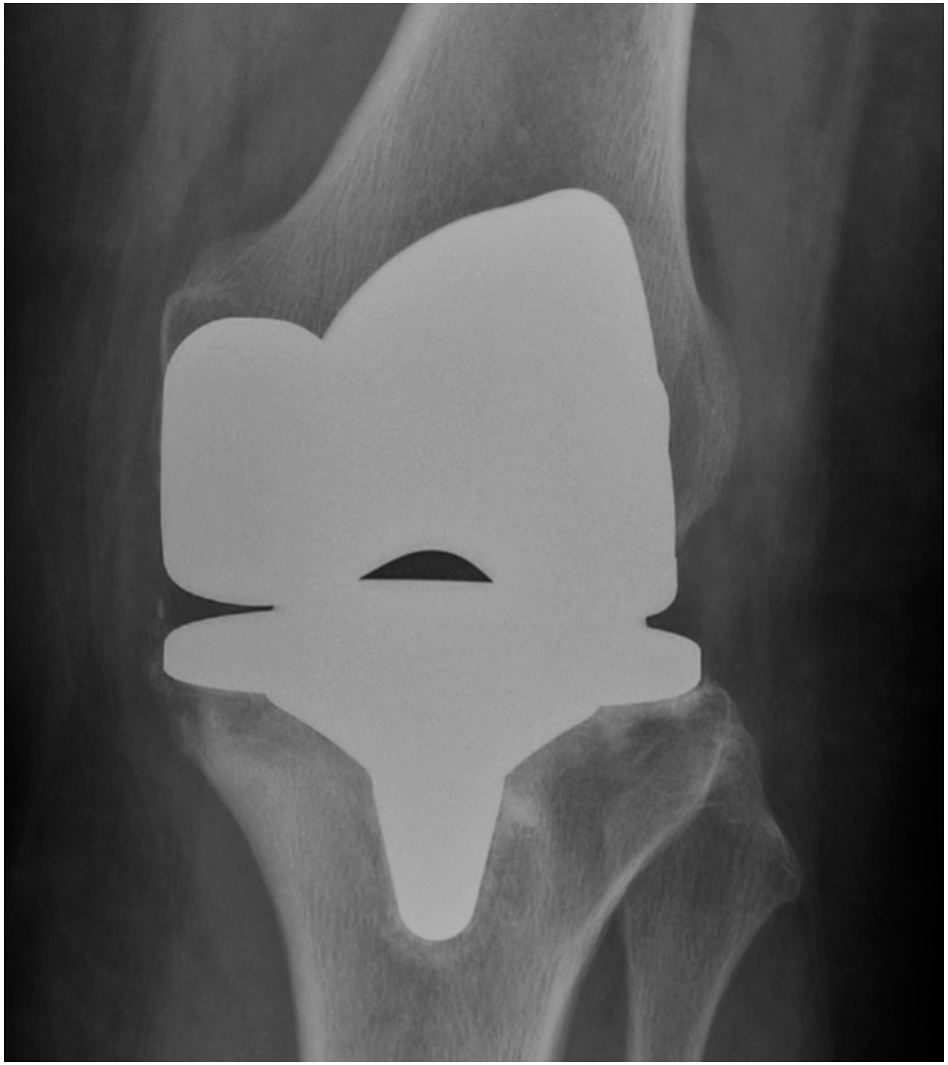

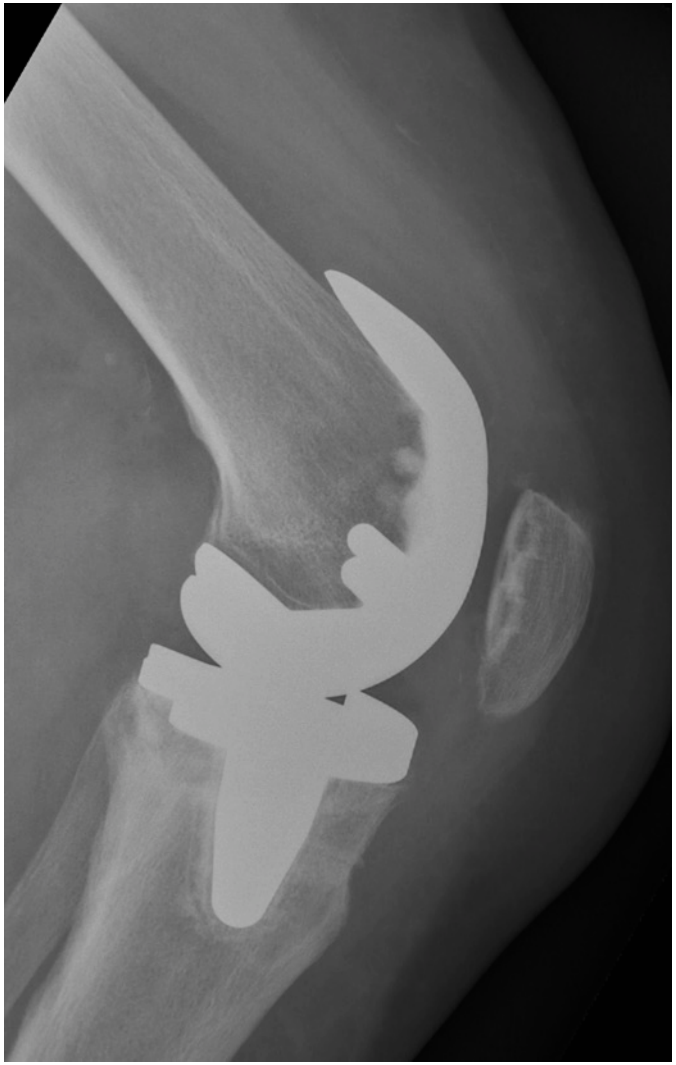

12 months postop

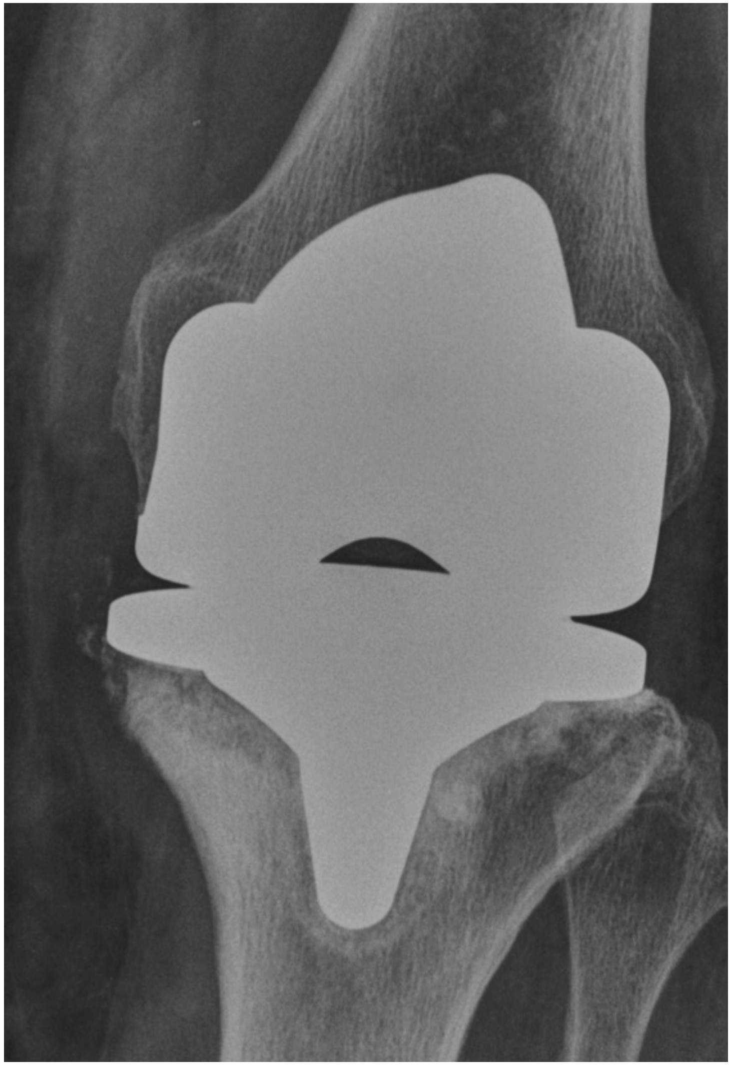

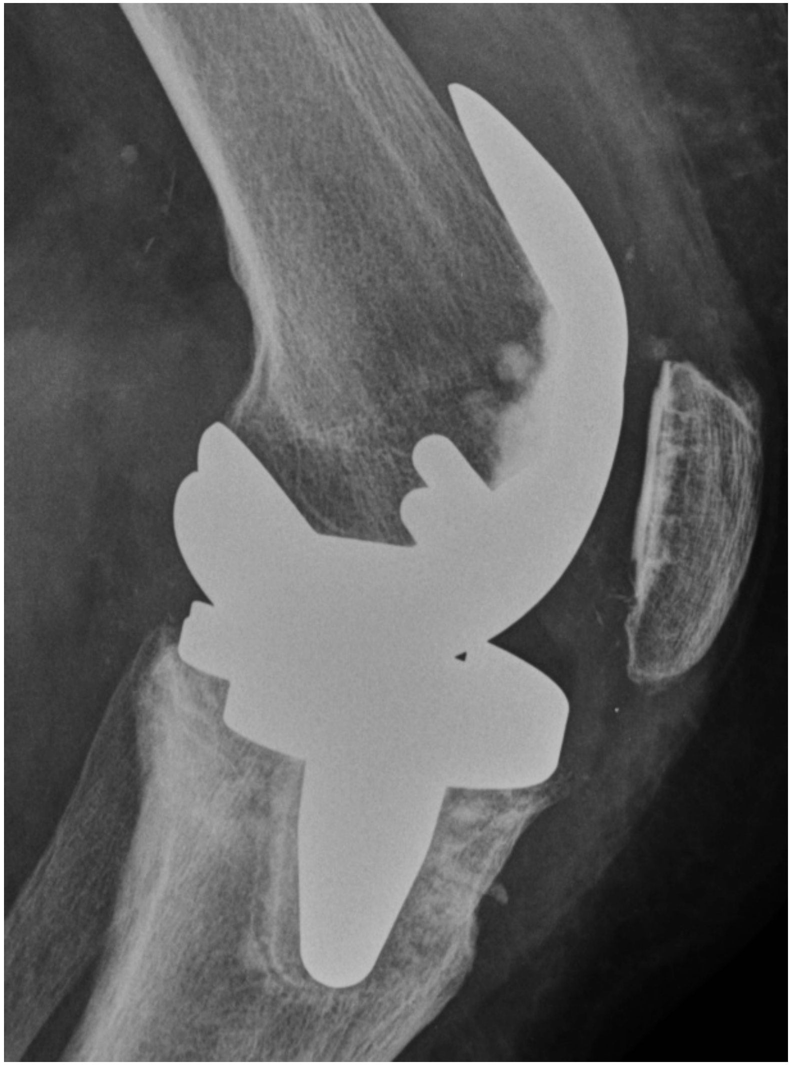

Case 14

preoperative

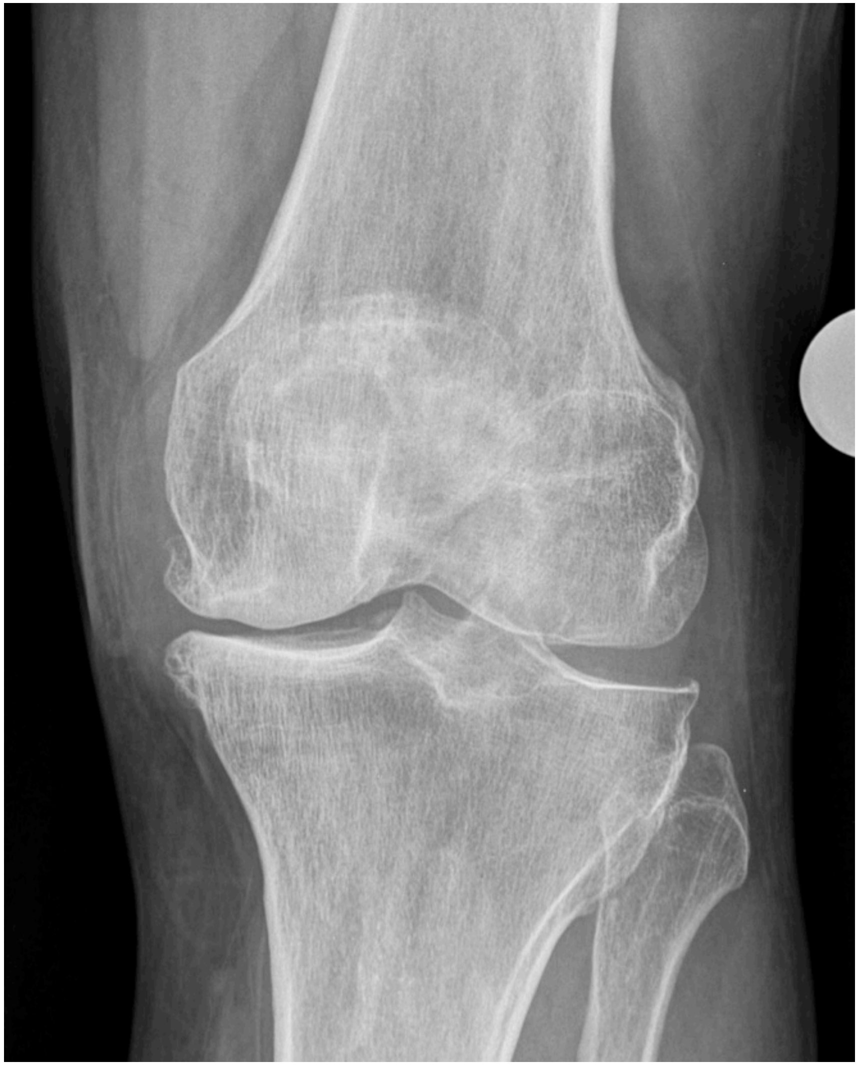
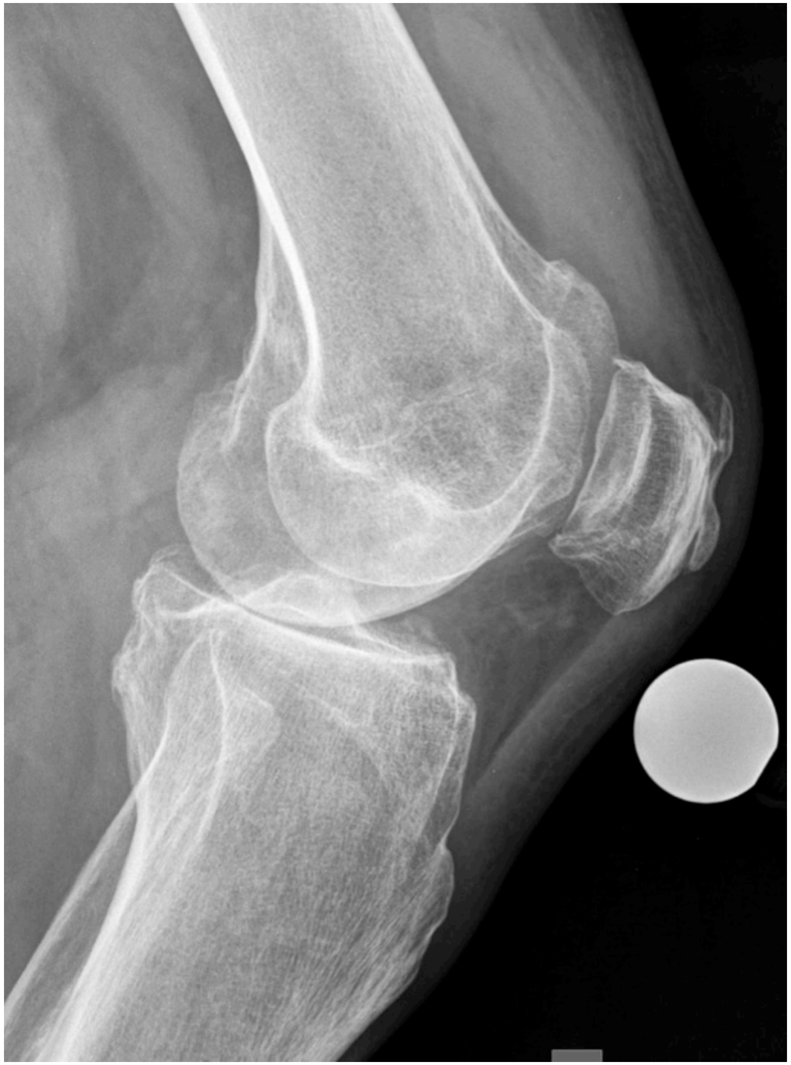

6 weeks postop

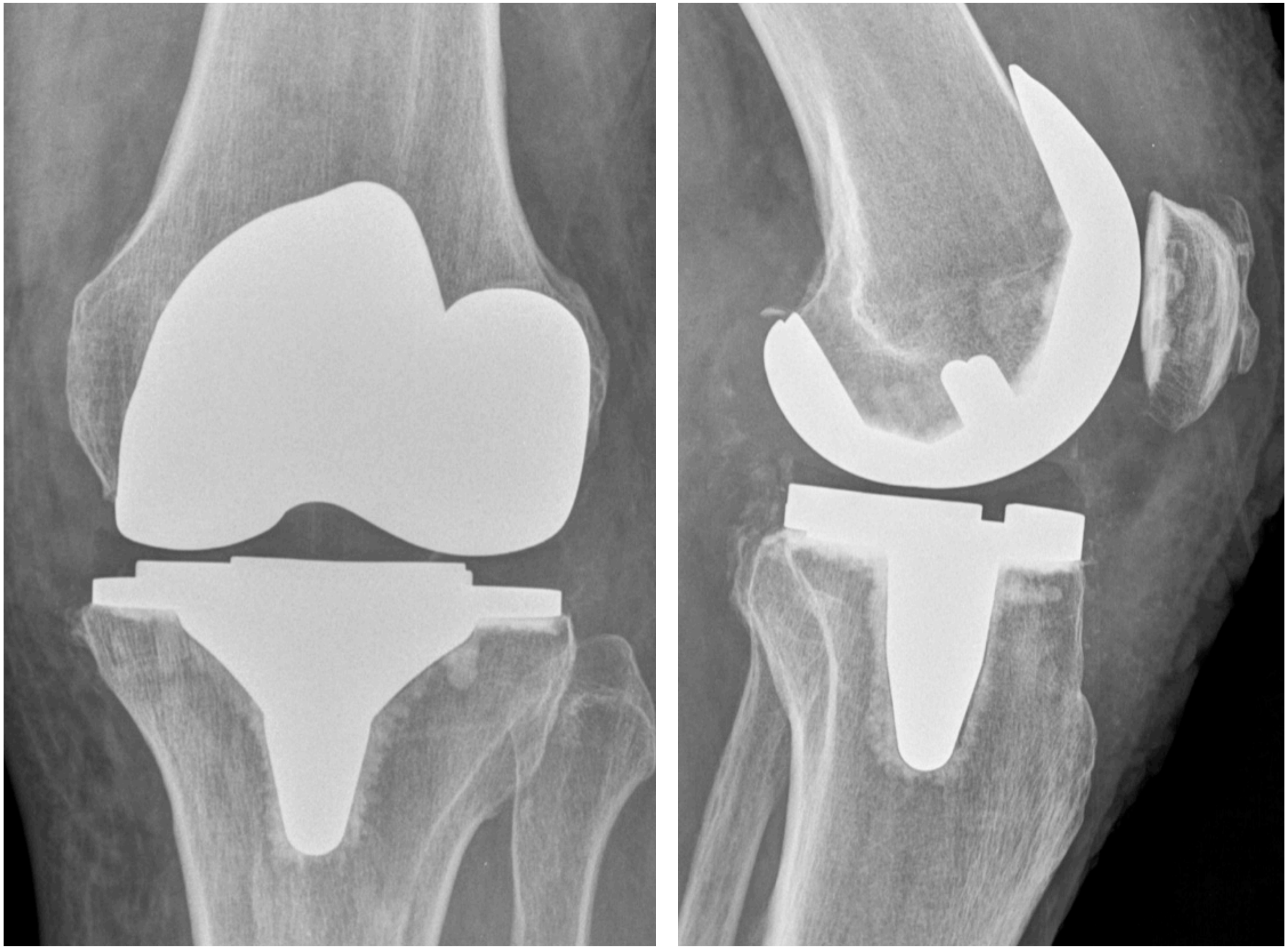

6 months postop

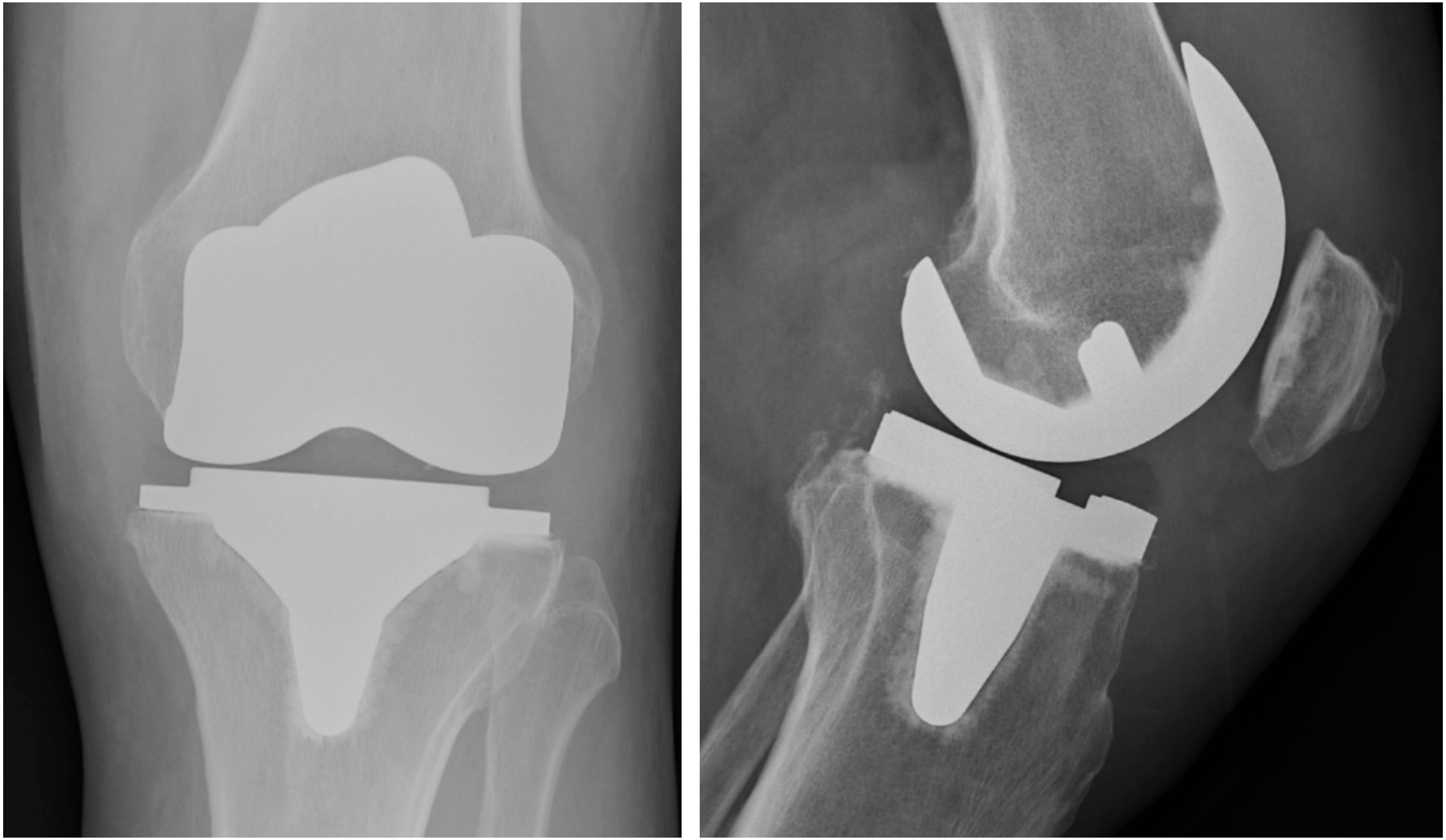

12 months postop

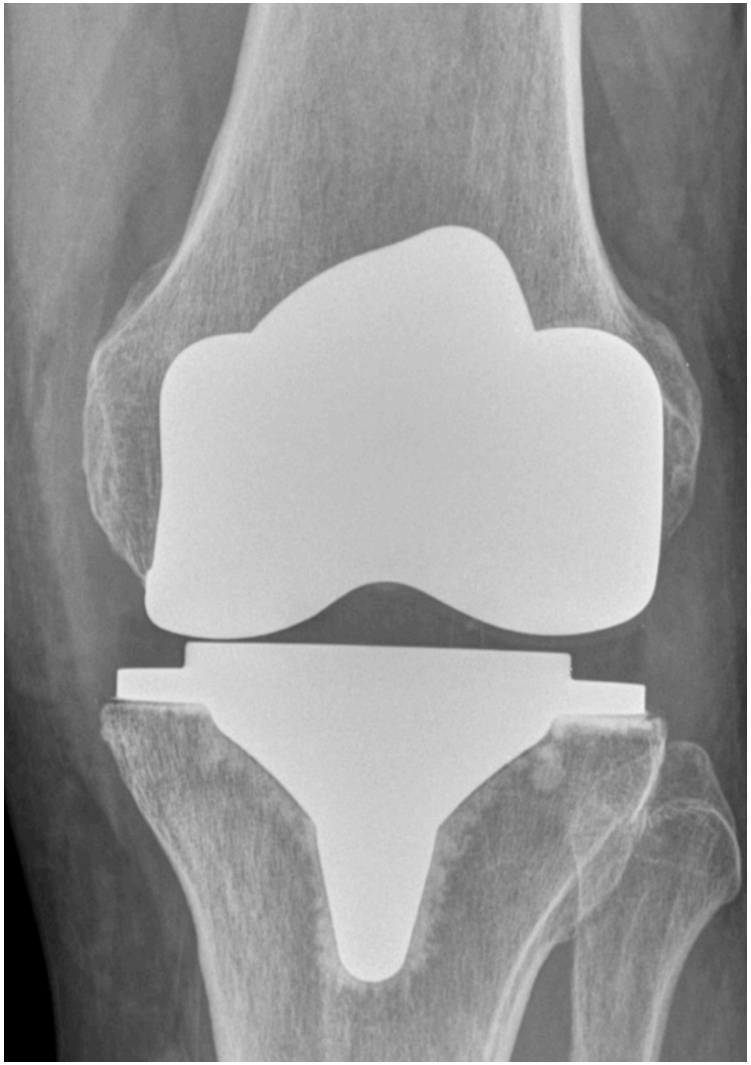

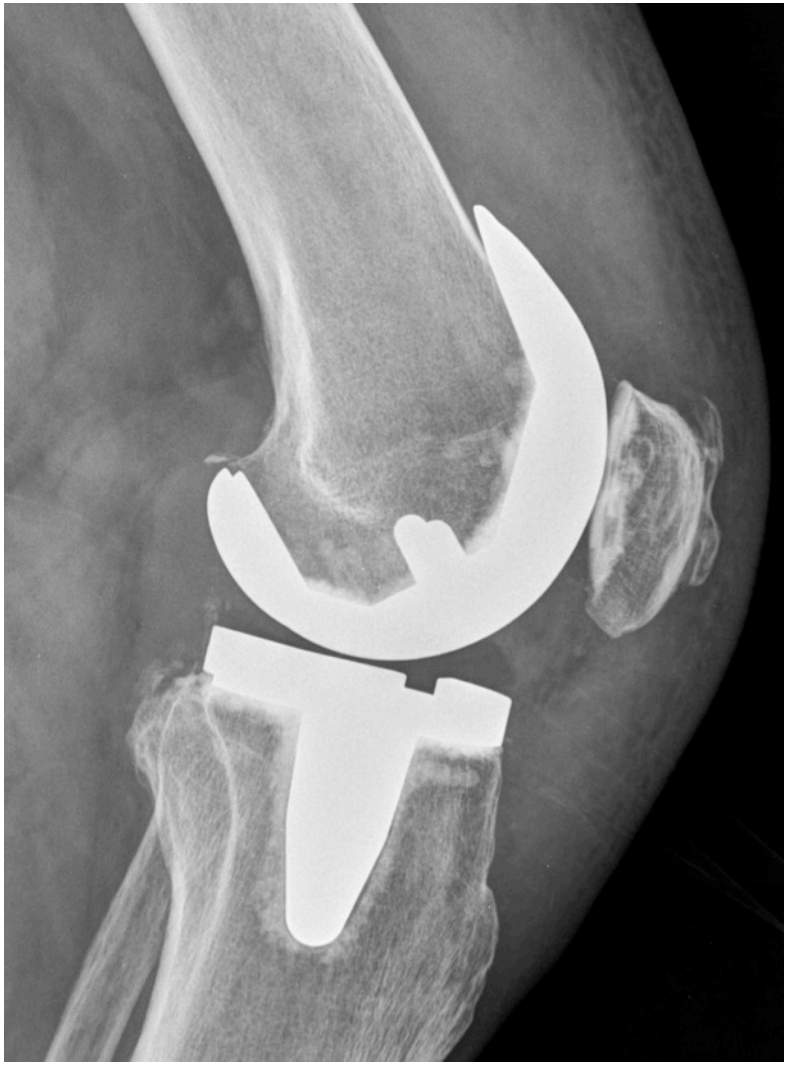

Case 15

preoperative

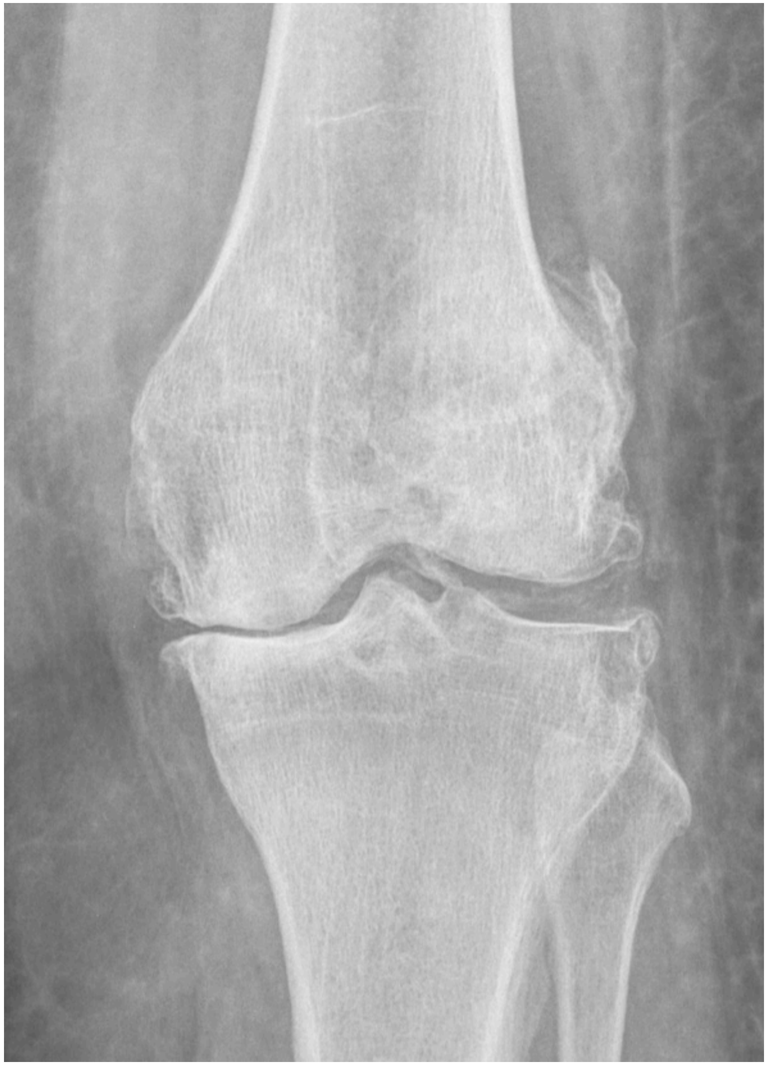

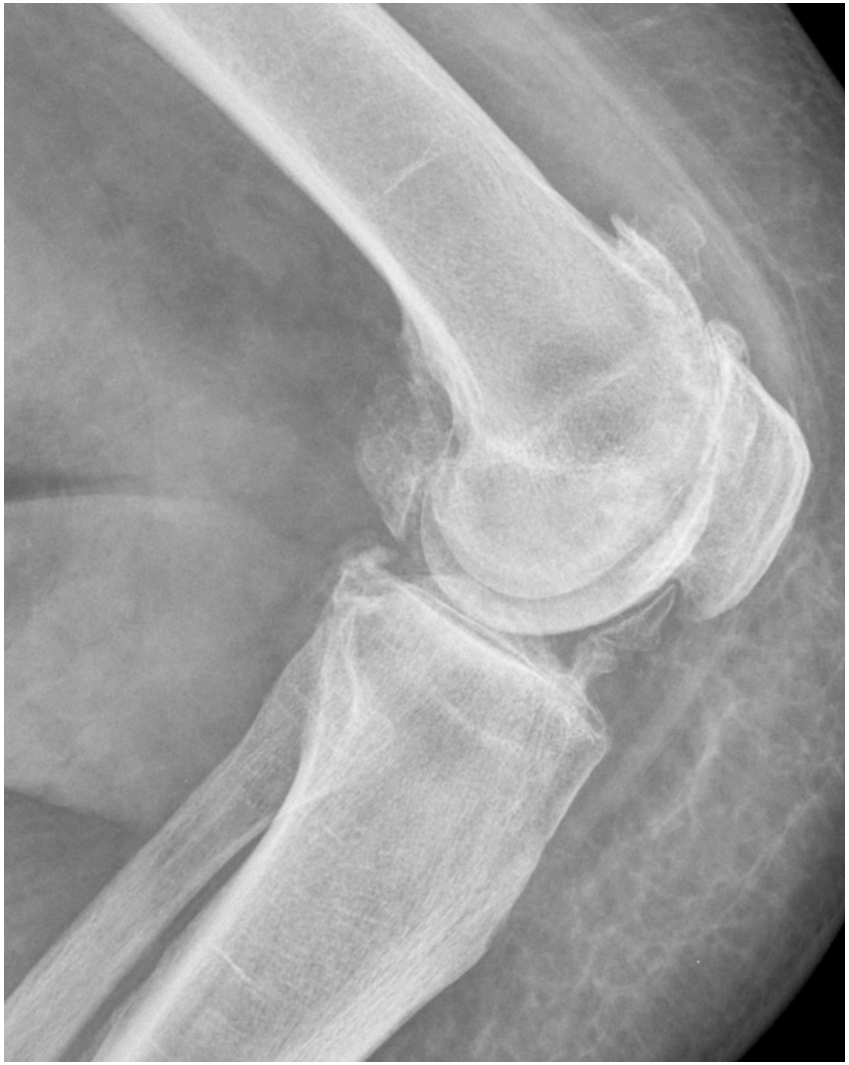

6 weeks postop

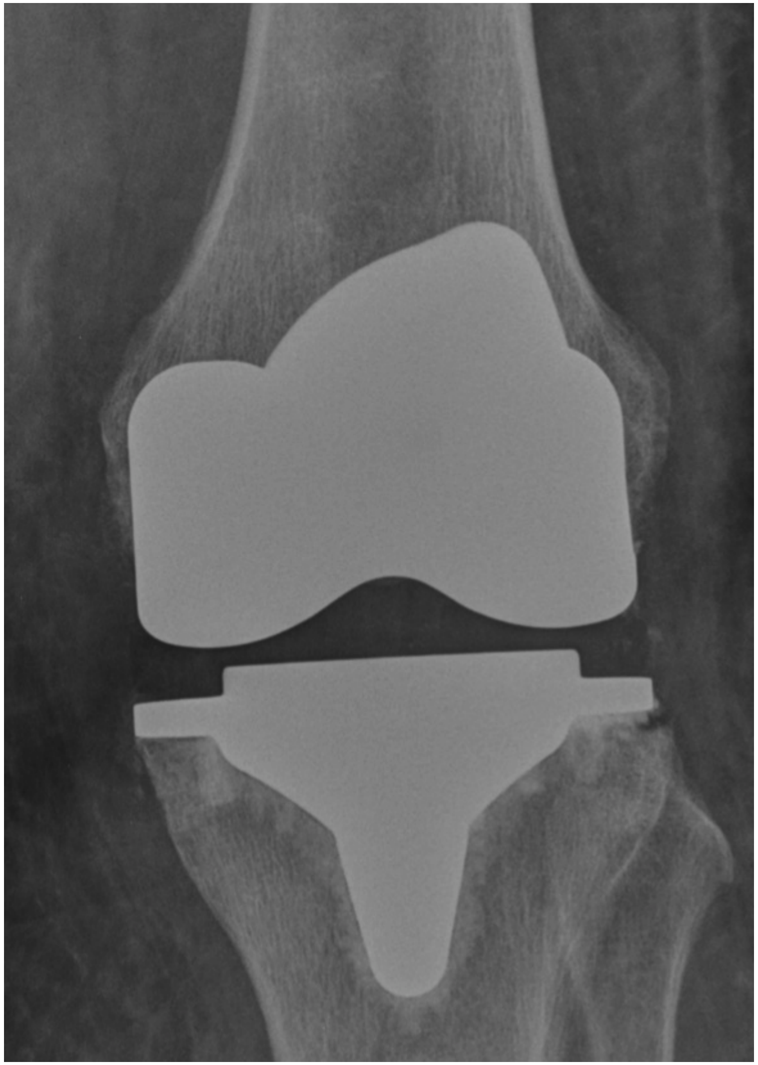

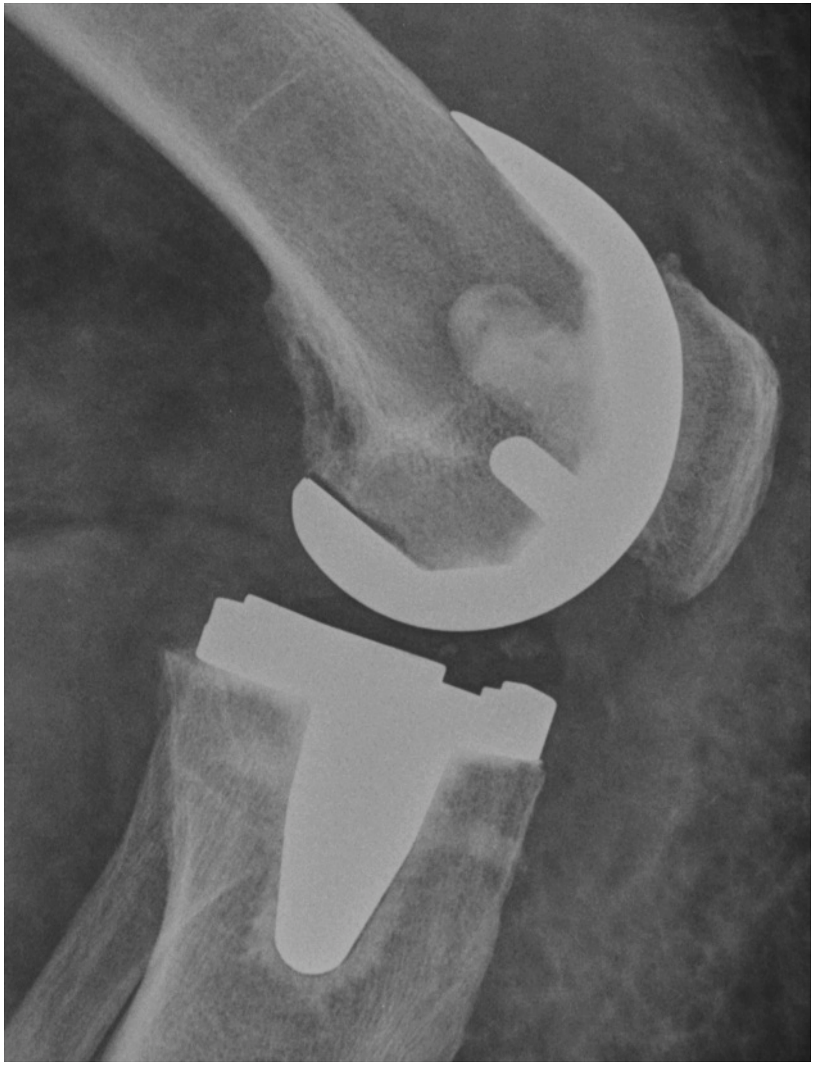

6 months postop

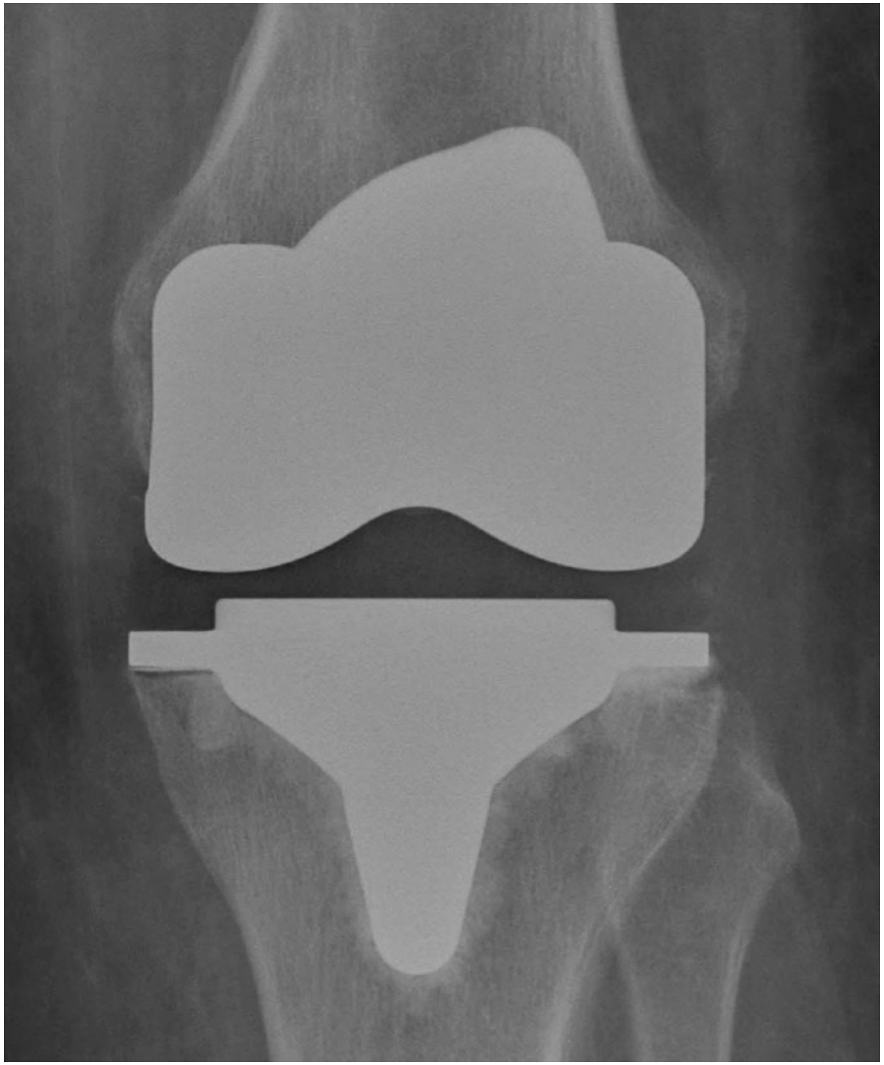
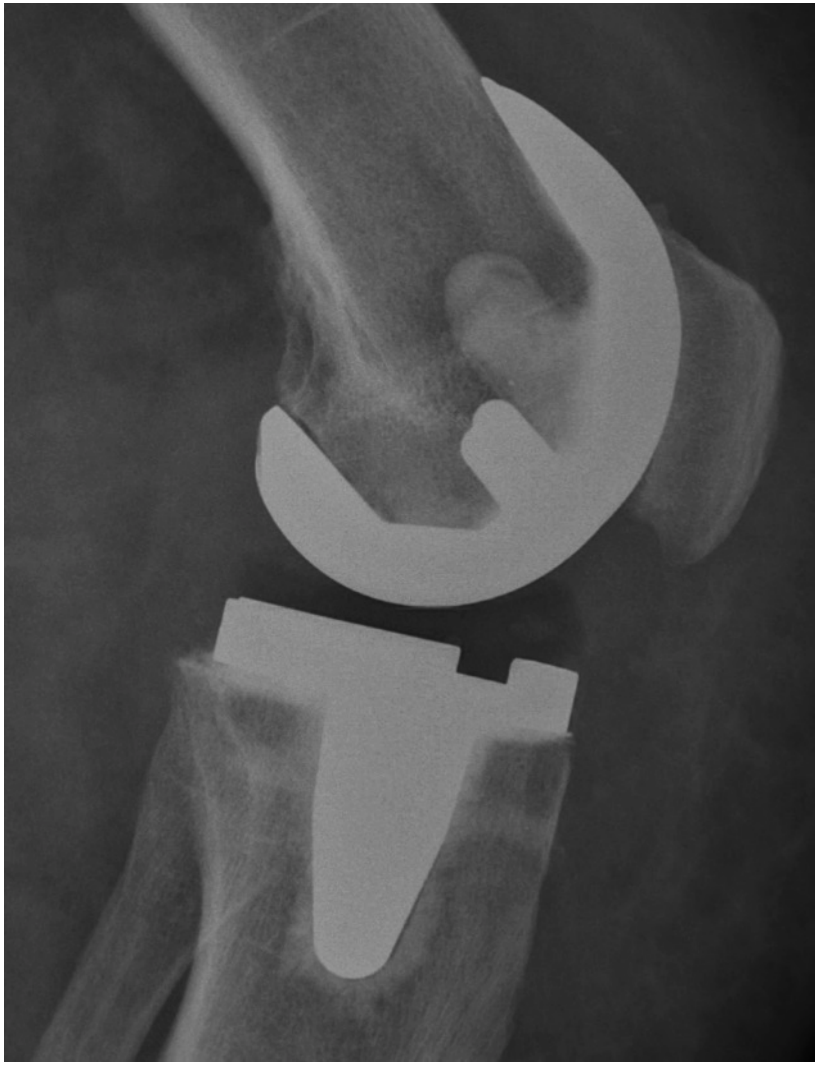

12 months postop

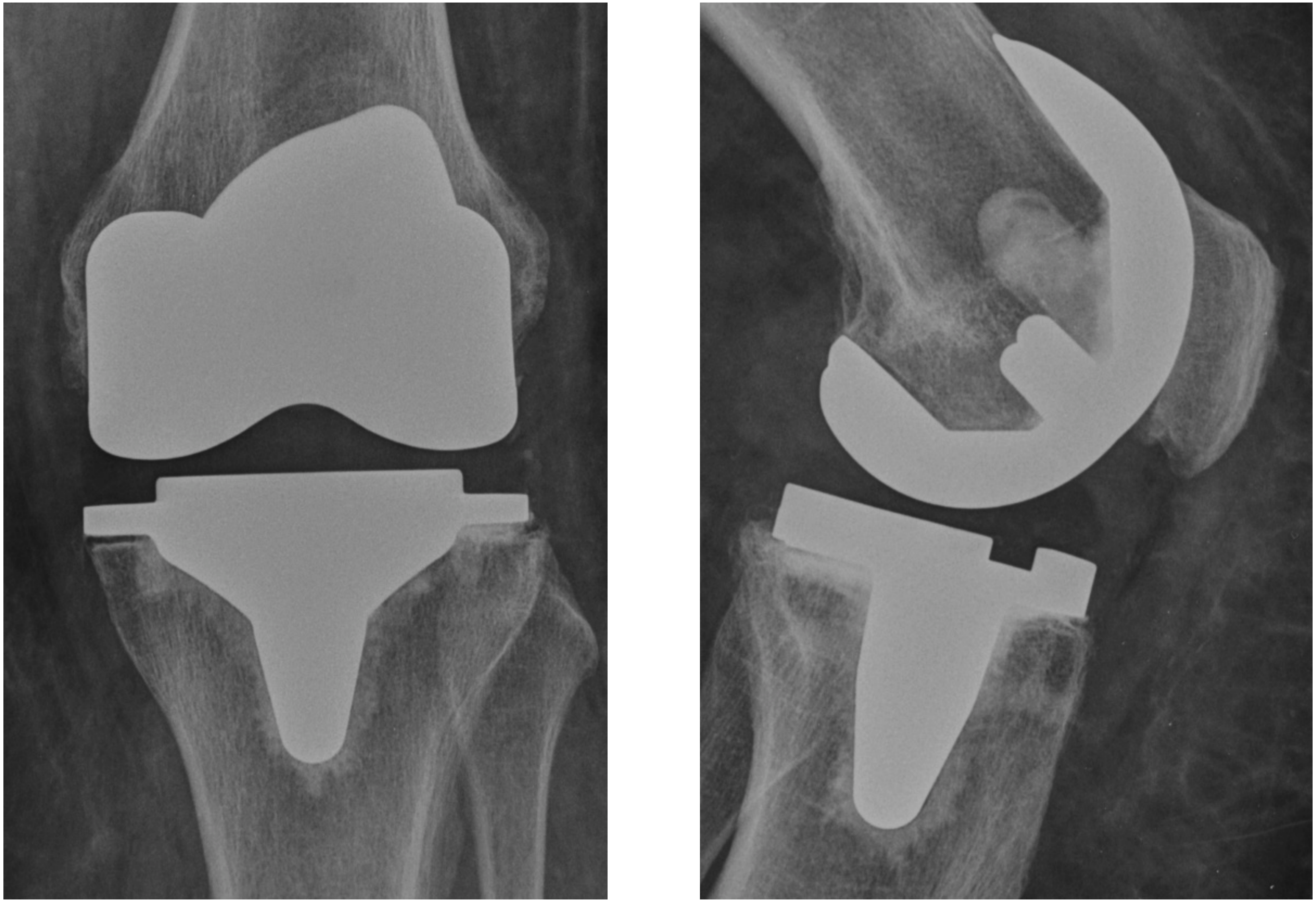

Supplement: Supplementary file 1 — Supplementary material 1 (PDF 21961 KB) [file 167_2018_5130_MOESM1_ESM.pdf]
