# Supplementary material for: Modern cemented total knee arthroplasty design shows a higher incidence of radiolucent lines compared to its predecessor
Source: Knee Surg Sports Traumatol Arthrosc. 2018 Sep 22;27(4):1148–55. doi: 10.1007/s00167-018-5130-0 (PMC6435629; doi:10.1007/s00167-018-5130-0)
Supplement: Supplementary file 2 — Supplementary material 2 (DOCX 21 KB) [file 167_2018_5130_MOESM2_ESM.docx]

**ADDENDUM – radiographic cases**

Case 1:

6 weeks postop:

- radiolucency at the medial baseplate in the ap radiograph

6 months postop and following:

- radiolucency at the medial tibial baseplate in the ap radiograph
- at the tibial keel in the ap- and lateral radiograph
- at the posterior femoral flange

Case 2:

6 months postop and following:

- radiolucency at the medial tibial baseplate in the ap radiograph
- at the anterior baseplate tibial keel lateral radiograph
- at the posterior femoral flange

Case 3:

6 weeks postop and following:

- radiolucency at the medial and lateral baseplate and at the medial side of the keel in the ap radiograph
- radiolucency at the anterior aspect of the keel

Case 4:

6 months postop and following:

- radiolucency at the lateral tibial baseplate in the ap radiograph
- at the posterior femoral flange

*The authors admit that the 6 weeks postoperative lateral radiograph is not suffieciently accurate and the radiolucencies (especially at the posterior femoral flange) may have been detectable at an earlier stage. Anyhow, the radiolucent lines are clearly visibile at the 6 months follow-up and subsequently at the annual follow-up.*

Case 5:

6 weeks postop and following:

- radiolucency at anterior aspect of the keel

6 months postop and following:

- at the posterior femoral flange

Case 6:

6 weeks postop and following:

- radiolucency at the lateral tibial baseplate in the ap radiograph
- at the lateral tibial keel in the ap radiograph
- at the anterior aspect of the keel

*There is also a radiolucent line at the medial baseplate, but since it is only seen in one radiograph, this was not declared as radiolucency*

Case 7:

6 weeks postop and following:

- at the anterior and posterior aspect of the keel in the lateral radiograph

6 months and following:

- radiolucency at the lateral tibial baseplate in the ap radiograph

Case 8:

6 weeks postop and following:

- at the anterior aspect of the keel in the lateral radiograph

Case 9:

6 weeks postop and following:

- at the anterior aspect of the keel in the lateral radiograph

6 months and following:

- radiolucency at the medial tibial baseplate in the ap radiograph

Case 10:

6 weeks and following:

- radiolucency at the medial and lateral tibial baseplate in the ap radiograph
- radiolucency at the lateral aspect of the keel in the ap radiograph

Case 11:

6 weeks postop and following:

- at the anterior aspect of the keel in the lateral radiograph

6 months and following:

- radiolucency at the medial tibial baseplate in the ap radiograph

Case 12:

6 weeks postop and following:

- radiolucency at the medial and lateral tibial baseplate in the ap radiograph
- at the posterior femoral flange in the lateral radiograph

6 months postop and following:

- at the posterior baseplate tibial keel lateral radiograph

Case 13:

6 months postop and following:

- radiolucency around the tibial keel in the lateral radiograph

Case 14:

6 weeks postop and following:

- at the anterior aspect of the keel in the lateral radiograph

Case 15:

6 weeks postop and following:

- at the anterior aspect of the keel in the lateral radiograph
- radiolucency at the medial and lateral tibial baseplate in the ap radiograph
- at the posterior femoral flange in the lateral radiograph
